# Efficacy of Two versus Three-Day Regimens of Dihydroartemisinin-Piperaquine for Uncomplicated Malaria in Military Personnel in Northern Cambodia: An Open-Label Randomized Trial

**DOI:** 10.1371/journal.pone.0093138

**Published:** 2014-03-25

**Authors:** Chanthap Lon, Jessica E. Manning, Pattaraporn Vanachayangkul, Mary So, Darapiseth Sea, Youry Se, Panita Gosi, Charlotte Lanteri, Suwanna Chaorattanakawee, Sabaithip Sriwichai, Soklyda Chann, Worachet Kuntawunginn, Nillawan Buathong, Samon Nou, Douglas S. Walsh, Stuart D. Tyner, Jonathan J. Juliano, Jessica Lin, Michele Spring, Delia Bethell, Jaranit Kaewkungwal, Douglas Tang, Char Meng Chuor, Prom Satharath, David Saunders

**Affiliations:** 1 US Army Medical Component, Armed Forces Research Institute of Medical Sciences, Department of Immunology & Medicine, Bangkok, Thailand; 2 Department of Medicine, Brigham and Women’s Hospital, Boston, Massachusetts, United States of America; 3 US Army Medical Component, Armed Forces Research Institute of Medical Sciences, Phnom Penh, Cambodia; 4 Royal Cambodian Armed Forces, Phnom Penh, Cambodia; 5 National Center for Parasitology, Entomology and Malaria Control, Phnom Penh, Cambodia; 6 Department of Medicine, University of North Carolina, Chapel Hill, North Carolina, United States of America; 7 Center of Excellence for Biomedical and Public Health Informatics (BIOPHICS), Mahidol University, Bangkok, Thailand; 8 Fast Track Biologics, Potomac, Maryland, United States of America; Mahidol-Oxford Tropical Medicine Research Unit, Thailand

## Abstract

**Introduction:**

Emerging antimalarial drug resistance in mobile populations remains a significant public health concern. We compared two regimens of dihydroartemisinin-piperaquine in military and civilians on the Thai-Cambodian border to evaluate national treatment policy.

**Methods:**

Efficacy and safety of two and three-day regimens of dihydroartemisinin-piperaquine were compared as a nested open-label evaluation within a malaria cohort study in 222 otherwise healthy volunteers (18% malaria-infected at baseline). The first 80 volunteers with slide-confirmed *Plasmodium falciparum* or *vivax* malaria were randomized 1:1 to receive either regimen (total dose 360mg dihydroartemisinin and 2880mg piperaquine) and followed weekly for up to 6 months. The primary endpoint was malaria recurrence by day 42. Volunteers with *vivax* infection received primaquine at study discharge with six months follow-up.

**Results:**

Eighty patients (60 *vivax*, 15 *falciparum*, and 5 mixed) were randomized to dihydroartemisinin-piperaquine. Intention-to-treat all-species efficacy at Day 42 was 85% for the two-day regimen (95% CI 69–94) and 90% for the three-day regimen (95% CI 75–97). PCR-adjusted *falciparum* efficacy was 75% in both groups with nearly half (45%) still parasitemic at Day 3. Plasma piperaquine levels were comparable to prior published reports, but on the day of recrudescence were below measurable *in vitro* piperaquine IC_50_ levels in all *falciparum* treatment failures.

**Conclusions:**

In the brief period since introduction of dihydroartemisinin-piperaquine, there is early evidence suggesting declining efficacy relative to previous reports. Parasite IC_50_ levels in excess of plasma piperaquine levels seen only in treatment failures raises concern for clinically significant piperaquine resistance in Cambodia. These findings warrant improved monitoring of clinical outcomes and follow-up, given few available alternative drugs.

**Trial Registration:**

ClinicalTrials.gov NCT01280162

## Introduction

Recent public health efforts have made considerable progress in reducing the morbidity and mortality of malaria in Southeast Asia. These advances are threatened by the emergence of documented artemisinin resistance in four countries including Cambodia, Myanmar, Thailand, and Vietnam [1]. Most notably, multi-drug resistant *Plasmodium falciparum* along the western Cambodian-Thai border is now resistant to both components of multiple artemisinin combination treatments (ACTs), emphasizing the importance of ongoing monitoring of antimalarial therapeutic efficacy and resistance in this region [1]–[4].

Dihydroartemisinin-piperaquine is a fast-acting, potent artemisinin derivative paired with a long-acting 4-aminoquinoline [5]. Several studies have demonstrated that dihydroartemisinin-piperaquine is safe and highly efficacious against multi-drug resistant *falciparum* although recent reports suggest declining efficacy in western Cambodia [2], [6]–[9]. National distribution as the first-line ACT for all malarial infection began in 2012, with introduction in 2006 in military personnel and in 2008 for Zone 1, a containment area of antimalarial resistance along the western Cambodian-Thai border [1], [2], [10].

At the time of this study, Cambodian national treatment guidelines recommended artesunate plus mefloquine combination therapy for *falciparum* infection and chloroquine monotherapy for *vivax* infection. However, military practice guidelines employed various two and three-day regimens of dihydroartemisinin-piperaquine under the brand names Artekin, Artequick, and Duocotexin, in order to improve compliance in austere settings. In anticipation of the Cambodian national malaria control (CNM) program’s plan to replace artesunate plus mefloquine with dihydroartemisinin-piperaquine for all species, we conducted a treatment study nested within a malaria cohort study in order to provide evidence-based treatment policy recommendations. The primary objective aimed to compare the efficacy of two and three-day regimens of dihydroartemisinin-piperaquine in a dynamic setting of emerging multi-drug resistance.

## Materials and Methods

### Ethics Statement

The study protocol was approved by specific institutional review boards at participating institution including Walter Reed Army Institute of Research (IRB#00000794), National Ethics Committee for Health Research in Cambodia (IRB#00003143), and University of North Carolina (IRB#00002074). This trial was registered prior to initiation (ClinicalTrials.gov identifier NCT01280162) and adhered to CONSORT guidelines. All participants provided written informed consent for participation, collection of samples, and subsequent analyses.

### Study Design and Participants

This was a two-arm, randomized, open-label trial nested within an active observational cohort study to compare the efficacy and safety of two versus three-day regimens of dihydroartemisinin-piperaquine in *falciparum* and *vivax* malaria. The study was conducted in part to identify potential sites to conduct antimalarial chemoprophylaxis studies. Between September 2010 and March 2011, otherwise healthy civilian and military personnel aged 18 to 65 years were enrolled into the cohort study at two sites in Oddar Meanchay province near the northern Thai border. Exclusion criteria included allergic reaction or other contraindication to dihydroartemisinin or piperaquine, pregnancy, lactation, or abnormal EKG including a QTc interval greater than 500 milliseconds. Volunteers developing slide-confirmed asexual *falciparum* and/or *vivax* parasites were randomized to receive a directly observed two or three-day course of dihydroartemisinin-piperaquine and assessed weekly for malaria for a minimum of 42 days. Volunteers with recurrent malaria received first-line therapy per national guidelines, which at the time of the study included artesunate-mefloquine for *P. falciparum* and chloroquine for *P. vivax*. Upon cohort study discharge, volunteers who had developed *vivax* infection were treated with commercially-obtained primaquine phosphate. Those with normal glucose-6-phosphate dehydrogenase (G6PD) activity received 30mg of oral primaquine daily for 14 days whereas G6PD-deficient volunteers received 45mg of oral primaquine weekly for eight weeks. To evaluate primaquine for *vivax* radical cure and relapse prevention, volunteers had passive follow-up via monthly clinic visit or telephone interview for 6 months after primaquine administration.

The primary efficacy endpoint was the first occurrence of any-species treatment failure by 42 days following dihydroartemisinin-piperaquine administration. Treatment failure was defined as any-species blood-stage recurrence before Day 42 to include mixed infection [11]. Secondary endpoints included species-specific treatment failure, piperaquine pharmacokinetics, drug resistance characterization, comparative safety outcomes including QTc prolongation, and 6-month post-primaquine recurrence rates.

### Randomization and Masking

Blocked randomization (computer-generated, fixed block size of two) assigned consecutive volunteers with positive malaria smear to receive either a two-day or three-day regimen, with treatment allocation masked from study staff and volunteers in sealed envelopes. A block size of 2 was chosen to maximize the comparability of treatment groups between enrollment sites, since malaria attack rates were not known a priori, and was variability was anticipated. Duocotecxin provided by Zhejiang Holley Nanhu Pharmaceutical Co., Ltd was procured by CNM. The two-day regimen (180mg dihydroartemisinin and 1440mg piperaquine given at time of diagnosis and then at 24 hours +/−1 hour) and three-day regimen (120mg dihydroartemisinin and 960mg piperaquine at time of diagnosis, at 24 hours and 48 hours +/−1 hour) both passed British Pharmacopeia 2004 content and uniformity standards conducted by AFRIMS Pharmacology Lab [3].

### Procedures

After enrollment, cohort volunteers had a baseline history and physical examination, 12-lead electrocardiogram (ECG), and laboratory evaluation [thick and thin smears, complete blood count (CBC), renal function, liver function, and G6PD deficiency via fluorescent spot test (SQMMR500, R&D Diagnostic). Volunteers had weekly assessment for symptoms of *Plasmodium* infection, and if symptom-free, monthly blood smears. Volunteers with clinical suspicion of malaria at routine follow-up, or at any time during the study, had blood smears. Two blinded microscopists examined Giemsa-stained thick and thin smears; a third blinded microscopist determined the final result for discordant readings. Parasite densities were calculated as a parasite count per 200 WBCs (thick smear) or per 5000 RBCs (thin smear). A total of 200 oil immersion fields were examined on the thick film before it was considered negative.

Volunteers with slide-confirmed malaria were admitted to an inpatient facility. Blood smears were made at 0 (first dose), 4, 8 hours, and then every 8 hours along with vital sign measurements. All treatment was directly observed and given with biscuits and lactasoy (320kcal, 17g fat) roughly 30–60 minutes before the first dose was administered. Patients were released to continued outpatient follow-up once afebrile with two consecutive negative smears and therapy completed. They were assessed weekly for malaria recurrence until cohort discharge, a minimum of 42 days. For volunteers developing blood stage *vivax* infection during the study, primaquine was administered upon cohort study discharge and directly observed. Pre- and post-primaquine (on day 3 up to day 28) hemoglobin levels were assessed for safety in patients with G6PD deficiency, with primaquine halted if greater than 25% hemoglobin drop was observed after the first dose at day 3.

Plasma piperaquine level sampling at 0, 4, 24, 48, and 72 hours after the first dose and weekly until day 42 or day of recurrence was performed using ultra-high performance liquid chromatography with tandem mass spectrometry analysis adapted from previous methods [5]. Briefly, 2 ml of whole blood was collected into chilled sodium heparin tubes, immediately centrifuged to separate plasma, frozen at approximately −20°C or below, and then transferred to Bangkok for analysis by UPLC/MS/MS using a Waters Acquity ™ Ultra Performance LC coupled with a Xevo TQ-S (Waters, Milford, MA, USA). Chromatographic separation was performed on an Acquity UPLC® BEH C18 1.7um, 2.1x50 mm analytical column with the same material used for the guard column at 40°C. The gradient mobile phase was comprised of 5% 200 mM Ammonium acetate pH 9.8, 28% ACN and 50% MeOH at a flow rate of 0.6 ml/min, using a sample injection volume of 2 μl with 5-minute run time. The Xevo TQ-S was equipped with an electrospray ionization source and operated in the positive ion mode. Data was acquired and processed using MassLynx version 4.1. Quantification was carried out using multi reaction mode (MRM) evaluating the transition of m/z from 535.22 to 261.17 and 288.20 (major) for piperaquine and 541.29 to 262.20 and 294.19 (major) SIL-PIP which was used as an internal standard. A two-compartment model with 1^st^ order absorption and elimination was developed using Phoenix WinNonlin version 1.3 (Pharsight, USA) to estimate pharmacokinetic parameters including the maximum concentration by interpolation (C_max_), time to maximum concentration (T_max_), area under the curve from time 0 to ∞ (AUC_0-∞_), α t_1/2_ (alpha – distribution half life) and β t_1/2_ (beta – elimination half life). Additional calculated parameter estimates included clearance divided by fraction absorbed from the central compartment (Cl/F), intercompartmental clearance (ClD2/F), absorption rate constant (k_01_), volume of distribution over fraction absorbed from central compartment (V1/F), and volume of distribution over fraction absorbed from the peripheral compartment (V2/F).

Fresh *falciparum* isolates obtained prior to drug administration were tested at 0, 4, 24, 48, and 72 hours in histidine-rich protein-2 enzyme-linked immunosorbent assays (HRP-2 ELISA) for susceptibility to common antimalarials [12]. Recurrent cases were distinguished as recrudescence or reinfection by genotyping for MSP-1 (merozoite specific protein), MSP-2, and GLURP (glutamate-rich protein) allelic variants [13]. Evaluation of *pfmdr1* copy number, *pfmdr1* single nucleotide polymorphisms (N86Y, Y184F, S1034C, N1042D, D1246Y), *pvmdr1* copy number, and *pfcrt* haplotyping of amino acid positions 72–76 were performed as previously described [14]–[20].

### Statistical Analysis

Region-specific preliminary data estimated a monthly incidence of 5–10% during peak malaria season. For the nested treatment study, sample size estimates were based on published dihydroartemisinin-piperaquine *falciparum* cure rates of approximately 75% and 98% for the two and three-day regimens respectively [21]. A total sample size of 76 was required to detect a 25% difference in efficacy with nominal 80% power using Fisher’s exact test with α = 0.05 (two-sided) (PASS, 2005).

Three analysis populations were defined: (1) intention-to treat (ITT) were all volunteers who received at least one treatment dose with those missing end-point data treated as failures, (2) per-protocol (PP) were all volunteers who completed treatment and who had at least 42 days of follow-up, and (3) modified intent-to-treat (mITT) was the ITT population; and, for mITT efficacy, persons withdrawn, lost-to-follow-up, or protocol violations were considered censored at the last study visit. For primaquine given at study discharge, ad hoc mITT and ITT analyses were conducted to estimate 6-month relapse prevention.

Descriptive statistics were used to summarize results. Mean values were compared using t-tests. Geometric means were compared using log-transformed values. The Wilcoxon rank sum test was used for non-parametric comparisons. Fisher’s exact test was used to compare two independent proportions. Confidence intervals (95%) were computed for all estimates of treatment effect measures with two-sided P-values reported.

The mITT population was used for primary efficacy analysis (all-species recurrence by Day 42) and for species-specific recurrence. Day 42 recurrence rates (95% CI) were estimated using the Kaplan-Meier method. Cumulative risk curves were compared using the Cox-Mantel (log-rank) test. *Falciparum*-specific analysis was PCR-adjusted to regard true *falciparum* recrudescence (alone or mixed infection in both initial and recurrent parasitemia) as failures and censored new *falciparum* or *vivax* infections as non-failures [22]. Dihydroartemisinin-piperaquine efficacy was also assessed in both the PP and ITT populations as the proportion of volunteers with PCR-corrected adequate clinical and parasitological response (ACPR) at Day 42. The ITT analysis treated those with missing endpoint data as failures.

Adverse events (AEs) were monitored from the time of antimalarial administration until study discharge and assessed for causality [23]. Given many symptoms attributable to acute malaria, we compared only symptoms that developed after dihydroartemisinin-piperaquine administration in the ITT population. A 12-lead ECG was performed at the time of infection and at 24 hour-intervals until treatment completion. QT intervals were manually measured and corrected using Bazett’s (QTcB = QT/√RR) and Fridericia’s (QTcF = QT/^3^√RR) formulae. If electronic QTcB reading was prolonged ≥500ms after dosing, study drug was halted, and an alternative agent was used for treatment. All statistical analyses were done using SAS® software, version 9.3 (SAS Corporation, Carey, NC).

## Results

**Figure 1:**
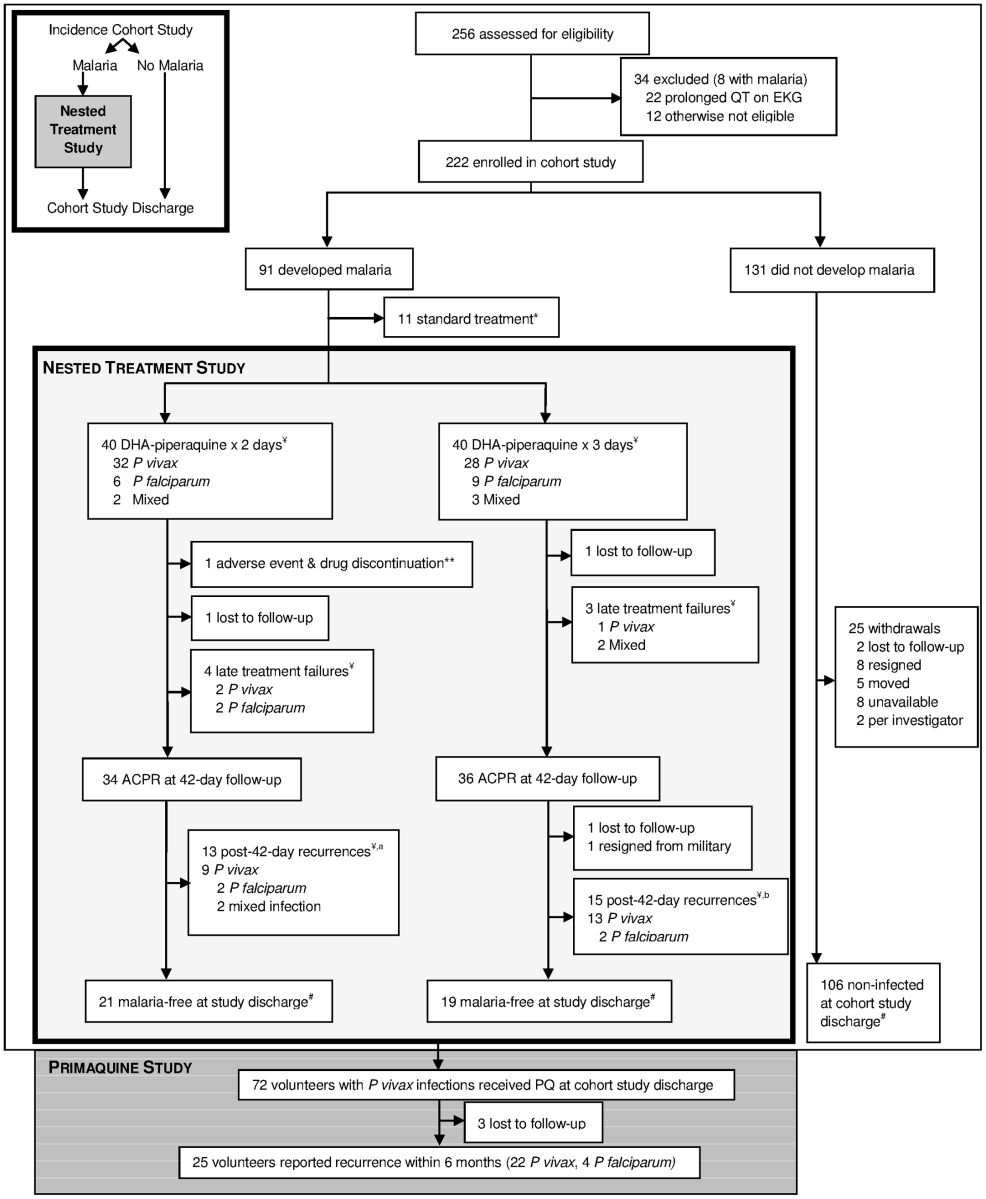
Study Schematic Inset and Trial Profile. *Randomization goal met, 2 withdrew. **Prolonged QTc. ^¥^PCR-corrected speciation. ^a^Not included are 2^nd^ recurrences (1 Pf, 1 Pv, 1 mixed). ^b^Not included are 2nd recurrences (2 Pf, 5 Pv) and a 3^rd^ recurrence (1 Pv). ^#^Study discharge varied, median 115 days follow-up.

Between September and December 2010, 256 asymptomatic volunteers screened for the cohort study, 222 enrolled, and 20% (51/256) were malaria-infected at baseline (39 *vivax*, 11 *falciparum*, 1 mixed) of whom 41 enrolled. Of the 91 developing uncomplicated malaria (Figure 1), the first 80 were randomized to dihydroartemisinin-piperaquine in a nested treatment study (Table 1). Of the 80, 75% had *vivax*, 19% had *falciparum*, and 6% had both species, with three lost to follow-up and one withdrawal. Gametocytemia was present at diagnosis in 56% (45/80) overall, and in 65% (39/60) of those with initial *vivax* infection.

**Table 1:** Baseline characteristics of enrolled patients according to treatment arm.

	2-day DHA-Piperaquine(n = 40)	3-day DHA-Piperaquine(n = 40)
Male patients	39 (97.5)	38 (95)
Weight (kg) (mean, SD)	57.5 (7.2)	57.6 (7.8)
Age (years) (mean, SD)	35.6 (8.3)	32.0 (8.9)
Military personnel	39 (97.5)	38 (95)
History of malaria in previous year	26 (65)	18 (45)
Temperature >37.5°C	11 (28)	17 (43)
G6PD deficiency by fluorescent spot test	8 (20)	5 (13)
Hematocrit (%) (mean, SD)	39.4 (4.3)	40.2 (5.4)
Species at primary infection*		
* P. falciparum*	6 (15)	10 (25)
Geometric mean parasites/μL (95% CI)	994 (211–4686)	2357 (596–9323)
* P vivax*	33 (80)	28 (70)
Geometric mean parasites/μL (95% CI)	499 (224–1112)	390 (177–858)
Mixed infections	1 (3)	2 (5)
Geometric mean parasites/μL (95% CI)	6130**	3284 (1197–9011)
Presence of *vivax* gametocytes	21 (53)	18 (45)
Presence of *falciparum* gametocytes	1 (3)	5 (13)

All data are number (%) unless otherwise indicated. There were no statistical or clinically significant differences between groups. *Non-PCR adjusted. **Only 1 sample in this subgroup so actual parasitemia reported.

Parasite and gametocyte clearance times were similar in each group (Table 2). Among 20 volunteers with *falciparum*, 75% were parasitemic at 48 hours and 45% at 72 hours. Median *falciparum* clearance time was 80 hours (IQR, 48, 88) in the two-day group and 68 hours (IQR, 44,104) in the three-day group (*P* = 0.84), while median asexual *vivax* parasitemia clearance was 8 hours in both groups.

**Table 2:** Therapeutic responses and PCR-corrected outcomes by treatment regimen.

		2-day DHA-Piperaquine(n = 40)	3-day DHA-Piperaquine(n = 40)	P-value
**Therapeutic responses**				
72-hour positivity, *(pos/n)*				
* P. vivax*		0/32	0/28	∼
* P. falciparum+*mixed		4/8	5/12	0.72
		[50 (22–78)]	[42 (19–68)]	
Parasite clearance time, *h,* median (IQR)		8 (8,24)	16 (8,40)	0.40*
* P. vivax*		8 (8, 16)	8 (6, 16)	0.96
* P. falciparum+*mixed		80 (48,88)	68 (44, 104)	0.96
Fever clearance time, *h*, median (IQR)		16 (10,17)	16 (8, 24)	0.88
Gametocyte clearance time, *h*, median (IQR)		16 (12,24)	20 (8,40)	0.86
**Efficacy outcomes**				
Early treatment failure, *n*		0	0	
Late treatment failure, *n*		4	3	
ACPR, PCR-adjusted, Day 42				
Per-protocol, all-species*		34/38	36/39	0.71
		[89 (75–97)]	[92 (79–98)]	
* P. vivax* **^¥^**		29/30	27/28	0.74
		[97 (83–99)]	[96 (82–100)]	
* P. falciparum* ^€^		6/8	9/11	0.73
		[75 (35–97)]	[82 (48–98)]	
ITT, all-species**		34/40	36/40	0.74
		[85 (70–94)]	[90 (76–97)]	
* P. vivax* **^¥^**		29/32	27/28	0.62
		[91 (75–98)]	[96 (82–100)]	
* P. falciparum* ^€^		6/8	9/12	1.00
		[75 (35–99)]	[75 (43–95)]	
Malaria-free at study discharge***				
Per-protocol, all-species		21/38	19/37	0.82
		[55 (38–71)]	[51 (34–68)]	
ITT, all-species		21/40	19/40	0.82
		[53 (36–69)]	[48 (32–64)]	

All data are number [%(95% CI)] unless otherwise indicated; CIs are based on binomial (exact) calculations; p-values are for Fisher’s exact test. *Wilcoxon test was used for comparing parasite and fever clearance times. ^¥^ *Vivax* recurrence in those with initial *vivax* infection. **^€^** *Falciparum* recurrences in volunteers with initial *falciparum* or mixed infections (n = 2 in each arm) were all true recrudescences by PCR. One subject with initial *falciparum* infection developed *vivax* infection at Day 42 and was counted as failure in the all-species analysis only. *PP analysis excludes subjects withdrawn or lost-to-follow-up. **ITT analysis treats subjects withdrawn or lost-to-follow-up as treatment failures. ***Mean (median) follow-up duration 102 days (115 days).

**Figure 2:**
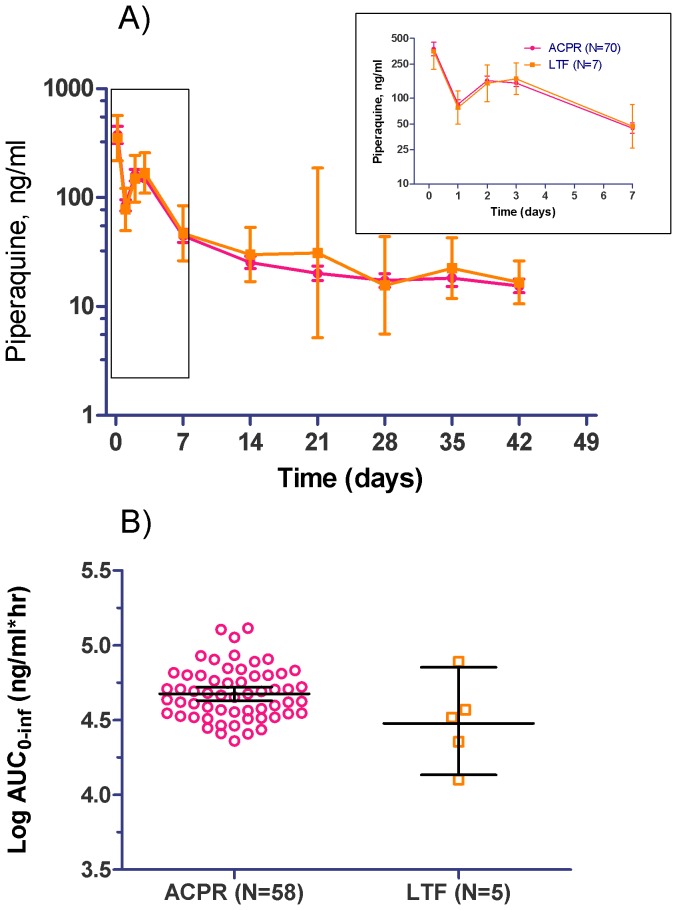
Comparison of plasma piperaquine levels between ACPR and recrudescent patients. (A) Concentration–time profiles (geometric mean concentrations with 95% CI) for ACPR and recrudescent groups. There were no statistically significant differences beween groups at any timepoint. (B) AUC_0-∞_ (2 compartment analysis) in ACPR and LTF groups. Black lines represent geomean with 95%CI. Note that AUC could not be calculated in 4 and 2 patients in the ACPR and LTF groups, respectively due to limited timepoints (only 3) in the terminal phase.

There were no early treatment failures (ETF). One volunteer with *vivax* infection had therapy switched after a QTc interval increase >100 milliseconds following the first dose of dihydroartemisinin-piperaquine and was censored as a treatment failure. There were seven late treatment failures (LTF) with the earliest (*P. vivax*) occurring at Day 14 in the two-day group. Plasma piperaquine concentration-time profiles were similar for LTFs and ACPR (Figure 2A). Geometric mean log AUC0-∞ where it could be calculated was not significantly lower in late treatment failures than ACPR using an unpaired T-test (Figure 2B).

**Figure 3:**
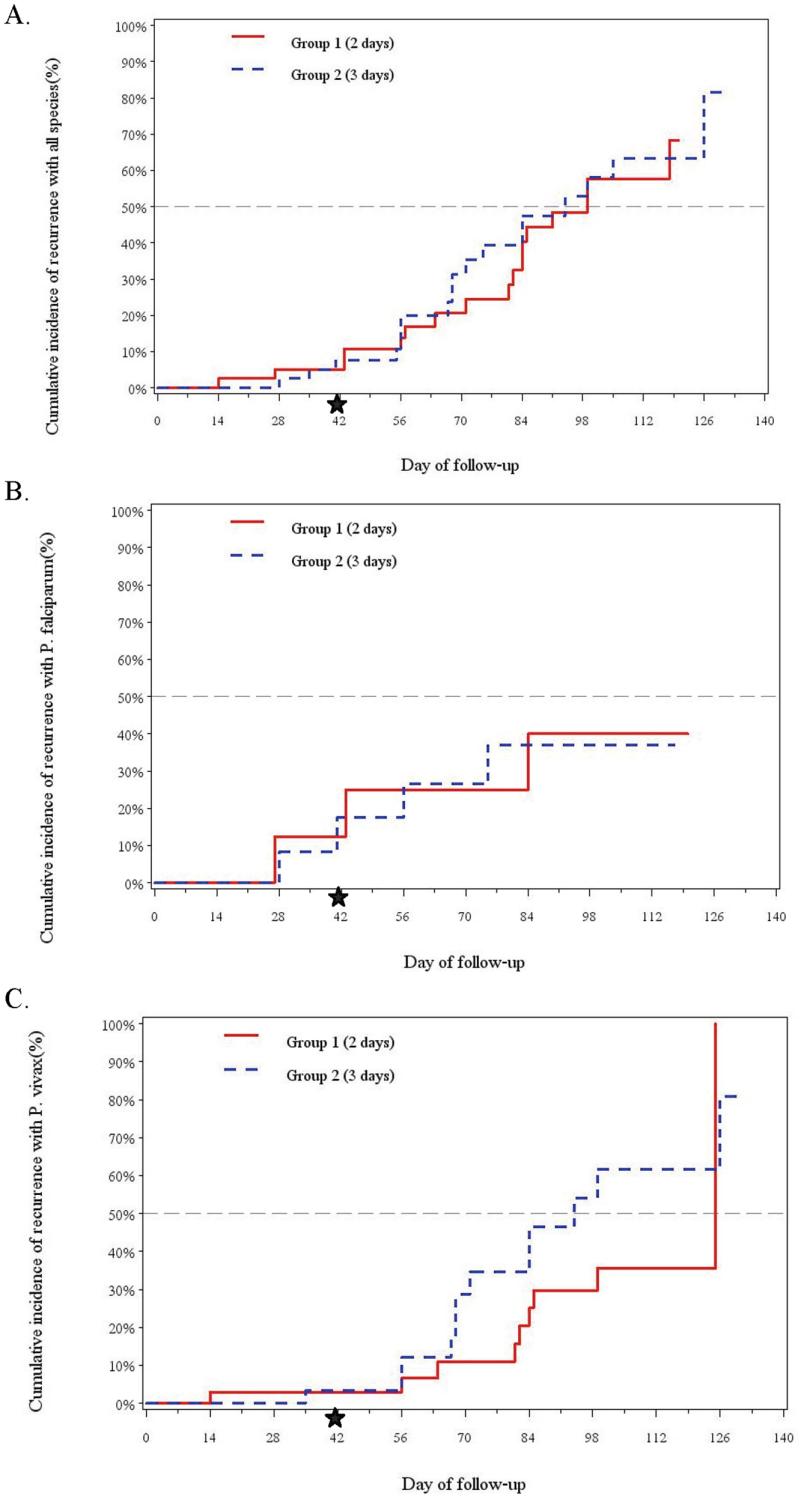
Cumulative incidence of recurrence by modified intention-to-treat analysis at Day 42 and study discharge. (A) No statistical difference between regimens for all-species recurrence at primary endpoint Day 42 (p = 0.63) and at day of discharge (p = 0.84). (B) In persons with an initial *falciparum* parasitemia (alone or mixed), there was no difference in true *falciparum* recrudescence at Day 42 (p = 0.73) and at day of study discharge (p = 0.91). (C) In persons with an initial *vivax* parasitemia (alone or mixed), there was no difference in *vivax* recurrence at Day 42 (0.94) and at day of study discharge (p = 0.22).

All-species efficacy by mITT analysis at Day 42, the primary endpoint, was 90% (95% CI, 7–19) for two-day and 92.5% (95% CI, 0–16) for three-day regimens (*P* = 0.63) (Figure 3A). All species intention-to-treat efficacy by day 42 was 85% (95% CI 70–94) in the two-day group versus 90% (95% CI 76–97) in the three-day group *P* = 0·74) (Table 2), with *vivax*-specific ITT efficacy 91% (95% CI 75–98) and 96% (95% CI 82–100) respectively (P = 0.62) (Table 2). Incidence of PCR-corrected *falciparum* recrudescence by Day 42 was 25% (95% CI, 0–55) and 17% (95% CI, 0–38) for two and three-day regimens respectively (P = 0.72), but 38% (95% CI 4–71) and 33% (95% CI 7–60) respectively by study discharge (P = 0.91) (Figure 3B), with ITT efficacy at Day 42 only 75% in both groups (95% CI 35–99 two-day versus 43–95 three-day) (Table 2). For those with initial *vivax* parasitemia, cumulative incidence of recurrence was 3% in each group at Day 42 (two-day 95% CI, 0–9; three-day 95% CI, 0–10) (Figure 3C). Regimens were comparable in the proportion remaining *vivax* malaria-free at study discharge with 42.5% for two-day (95% CI, 27–58) versus 45% for three-days (95% CI, 30–60) (*P* = 0.84).

**Figure 4:**
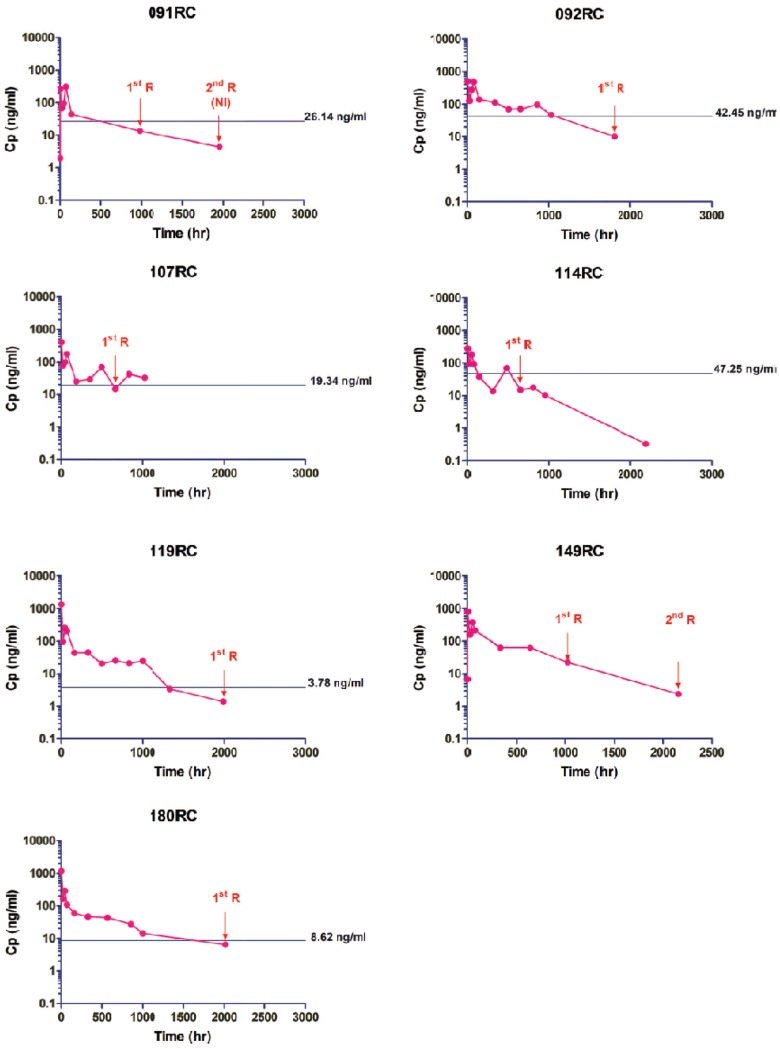
Piperaquine plasma concentration-time profiles in relation to IC_50_ for cases of *P. falciparum* recrudescence. The pink line represents the plasma piperaquine concentration, and the green line represents Day 0 IC_50_, with time of recurrence noted by arrows. Plasma piperaquine is shown as piperaquine phosphate salt concentration (MW 999·56) to match IC_50_ calculation method. NI = new infection in case 091. The IC_50_ for 149RC could not be assessed due to non-recovery of parasites from the clinical sample.

While largely indistinguishable, pharmacokinetic parameters for piperaquine calculated using a slow and fast clearing 2-compartment model revealed significantly higher C_max_, AUC_0-∞_ and k_01_ with the 2 day course but lower terminal elimination half-life (β t_1/2_) compared to the 3 day course (Table 3). *Ex vivo* drug susceptibility was assessed in 12/16 (75%) isolates from *falciparum* cases at Day 0 and compared to 50% inhibitory concentration (IC_50_) values at recurrence (Figure S4). Average IC_50_s at day 0 were higher in recrudescent than non-recrudescent cases, though the limited number of paired samples prevented assessment of statistical significance. In all evaluable cases of *falciparum* recrudescence, plasma piperaquine levels on day of recurrence were below Day 0 IC_50_ whereas levels in all ACPR cases were above Day 0 IC_50_ (Figure 4).

**Table 3:** Pharmacokinetic parameters calculated from a 2-compartment model comparing two versus three-day courses of DHA-piperaquine.

Pharmacokinetic parameter	All Subjects	Treatment Group		
		2 day course	3 day course	P-Value*
N	63**	30**	33**	
C_max_ 1^st^ dose (ng/ml)	287 (188–282)	**391** (228–699)	**245** (153–313)	**0.003**
T_max_ 1^st^ dose (hr)	3.87 (3.44–4.51)	3.69 (3.23–4.42)	3.94 (3.67–4.73)	0.084
AUC_0-∞_ (hr **·** μg/ml)	23.3 (17.7–33.8)	**29.1 (20.9–36.6)**	**19.8 (17.4–26.1)**	**0.004**
α t_1/2_ (hr)	13.9 (10.4–25.3)	13.9 (10.2–25.6)	14.2 (10.6–25.2)	0.896
β t_1/2_ (days)	24.7 (18.2–33.3)	**21.0** (16.0–28.1)	**27.8** (19.7–34.1)	**0.029**
Cl/F (L/hr)	25.9 (20.2–33.0)	26.5 (21.1–36.9)	25.9 (19.7–29.7)	0.390
ClD2/F (L/hr)	44.3 (30.0–69.6)	39.5 (25.4–55.6)	53.7 (31.7–77.6)	0.077
K_01_ (L/hr)	0.802 (0.677–0.932)	**0.850** (0.748–0.975)	**0.767** (0.628–0.900)	**0.047**
V1/F (L)	1734 (957–2958)	1639 (916–2915)	1778 (1243–2991)	0.549
V2/F (L)	13008 (9302–19519)	12159 (8773–17155)	15555 (9733–19856)	0.106

*Mann-Whitney U test comparing 2 and 3 day treatment groups. **PK analysis could not be performed in 10 and 7 subjects from Group 1 and 2, respectively due to insufficient time points. PK Parameters are expressed as medians and *(25–75% percentiles)*, with significantly different values in **bold**.

Though multiple *pfmdr1* copy number at baseline (>1.5) was more common in recrudescent (3/7), than non-recrudescent cases (0/13), there was no difference in median Day 0 *pfmdr1* copy number between the 7 recrudescent and 13 non-recrudescent *falciparum* cases [1.22 (IQR 1.00, 1.16) versus 1.12 (IQR 1.14,1.61); p = 0.104]. Copy number increased relative to Day 0 in all three cases of secondary recurrence (Figure S1). All *falciparum* isolates except one contained the184F*pfmdr1* mutation, but wild-type at codons 86N, 1034S, 1042N, 1246D, and all had the *pfcrt* CVIET mutant haplotype associated with chloroquine resistance. Increased *pvmdr1*copy number in *vivax* isolates was uncommon (7% of all isolates) and did not increase significantly with recurrent parasitemia (Table S1) [20].

**Figure 5:**
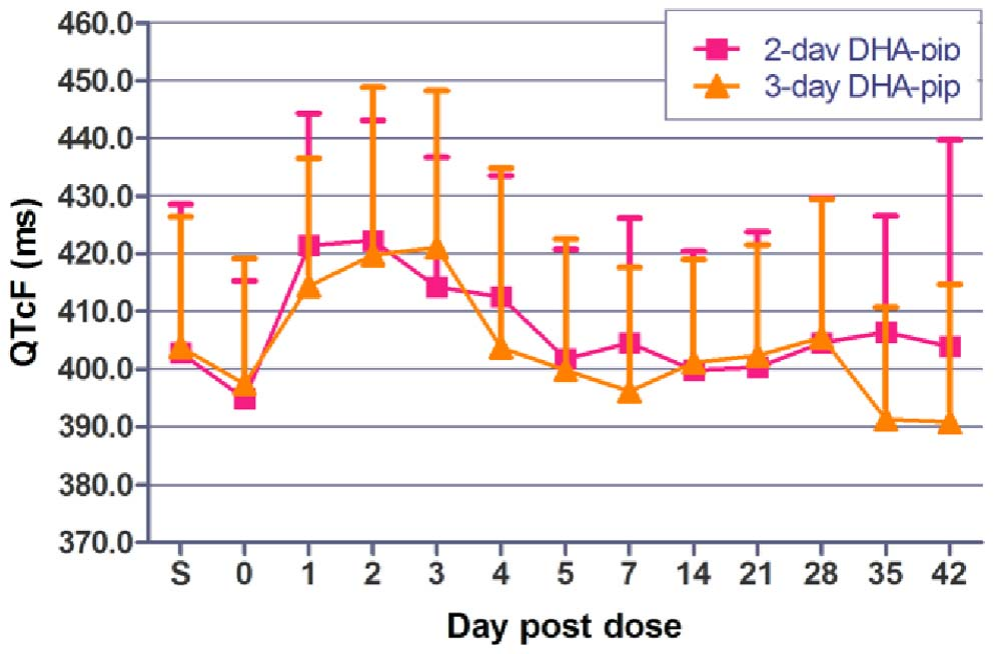
Mean trough QTcF intervals for patients treated daily with DHA-piperaquine (360/2880 mg cumulative) divided over 2 or 3 days (n = 40 in each group). Error bars indicate +1 standard deviation.

The most commonly reported adverse events were upper respiratory infection, headache, and musculoskeletal pain. Only 10% of volunteers developed new complaints not present at baseline following dihydroartemisinin-piperaquine treatment (Figure S2). The only serious adverse event (SAE) reported was an unrelated hospitalization for motorcycle accident six weeks after dihydroartemisinin-piperaquine administration. There was a mean QT prolongation of 20–30ms (Figure 5) between pre-dose and trough drug piperaquine levels, and roughly 18.5% of the 80 subjects dosed had a grade 1 cardiac adverse event due to QT prolongation during the 3 day period following dosing (Table 4). There were no significant differences in adverse cardiac events between the regimens on any of the days measured (Table 4).

**Table 4:** QTcB and QTcF interval changes according to treatment arm.

Changes in mean QTc intervals(msec) from screening					Adverse Events(based on QTc prolongation in msec)				
Group	n	Mean at screening	Mean maximum	Mean % increase	Grade 1(450–480)	Grade 2(480–500)	Grade 3 (>500)	Total AEs	% with AE
**QTcB**									
2-day	40	419.5	444.1	6.1	11	3	–	14	35
3-day	40	421.2	442.5	5.1	10	–	1	11	27.5
**QTcF**									
2-day	40	403	433	7.8	4	3	–	7	17.5
3-day	40	404	429	6.3	7	–	1	8	20

Mean changes from baseline in QTcB and QTcF following daily treatment with DHA-piperaquine (360/2880 mg cumulative dose) divided over 2 versus 3 days.

**Figure 6:**
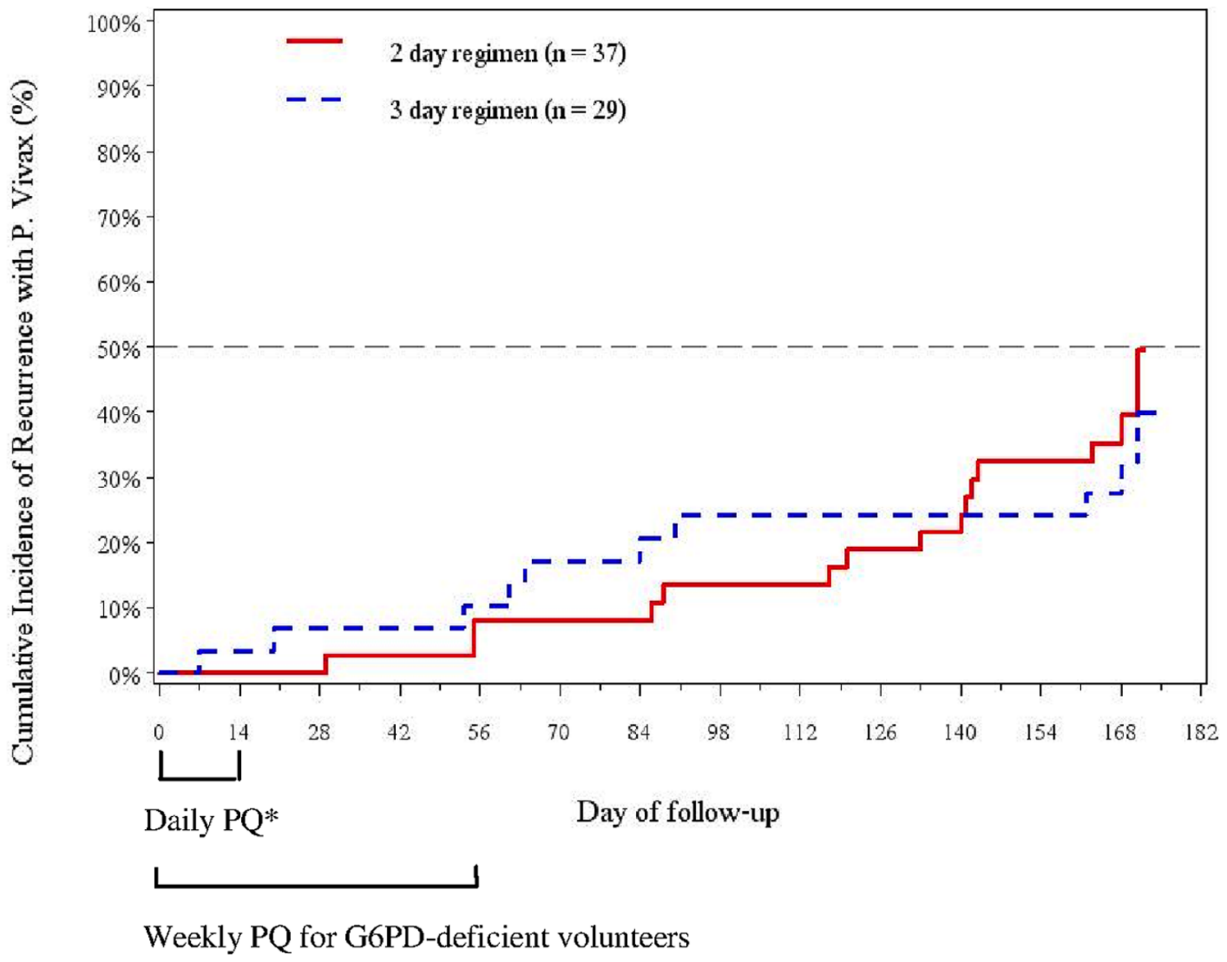
Six-month cumulative incidence of reported vivax recurrence following primaquine administration. Cumulative incidence (Kaplan-Meier) of reported *vivax* recurrence in 2-day (n = 37) versus 3-day (n = 29) regimens following primaquine (PQ) administration at study discharge by monthly follow-up in 66 volunteers over 6 months (P = 0.62). Six cohort volunteers who were not randomized to dihydroartemisinin-piperaquine treatment also developed vivax infection and received primaquine, but are not included in analysis above. *G6PD-normal volunteers received daily primaquine30mg for 14 days whereas G6PD-deficient volunteers received a weekly regimen of 45mg for 8 weeks.

At cohort study discharge, 72 volunteers developing *vivax* infection received directly observed primaquine treatment. Thirteen volunteers were qualitatively G6PD-deficient and received a weekly 45mg primaquine dose for eight weeks. Prior to starting primaquine, the mean (SD) baseline hematocrit for the G6PD-deficient group was 41.2 (3.7) mg/dL (95% CI, 39.0–43.4), similar to the mean follow-up hematocrit within 1 month of initiating therapy [40.4 (4.9) mg/dL (95% CI, 38.5–42.3; P = 0.60)]. Only two G6PD-deficient volunteers had>10% reduction from baseline hematocrit, dropping 18% and 14% below baseline at 19 and nine days, respectively, and both resolved within one month with iron and multivitamin supplementation. Six months of passive follow-up was completed in person or by phone for 96% (69/72) of volunteers, the majority of whom remained in the transmission area. Twenty-six volunteers had recurrences (25 *vivax*, 4 *falciparum*) with no difference between standard and weekly primaquine regimens (p = 0.19) (Figure S3), with a six-month ITT efficacy for *vivax* recurrence of 61% (44/72; 95% CI, 50–72) (Figure 6).

## Discussion

In an open-label, randomized trial of two and three-day dihydroartemisinin-piperaquine regimens, we could not distinguish a difference in efficacy for treatment of all-species malaria. We did find that 42-day treatment failure rates (7.5% and 10% respectively) were higher than previously reported; however, the small numbers of treatment failures limits the ability to draw conclusions on emerging *falciparum* resistance patterns. The majority of published studies demonstrated dihydroartemisinin-piperaquine treatment failure rates of 0–5% in both Africa and Southeast Asia [6]–[8]. This is particularly concerning for a combined malaria endpoint, selected to provide a ‘real world’ assessment of efficacy prior to introduction as first-line therapy for all malaria in Cambodia, given the poor *falciparum* efficacy seen (as low as 75% at Day 42). Prolonged parasite clearance times with 45% Day 3 positivity were observed despite relatively low initial parasitemia and DOT. Dihydroartemisinin-piperaquine was efficacious against blood-stage *vivax* with treatment failure as low as 3% at Day 42. A recent report indicated *falciparum* treatment failure rates as high as 25% in western Cambodia, worsening over three years (2008–2010), but 100% efficacy in Preah Vihear province [9]. Despite small numbers of *falciparum* malaria here, our findings suggest that the decline in efficacy of dihydroartemisinin-piperaquine may have already spread to northern Cambodia as early as 2010.

More importantly, treatment failures had Day 0 parasite *ex vivo* IC_50_ levels in excess of plasma piperaquine levels at the time of recrudescence, providing evidence for clinical piperaquine resistance. This is concerning given dihydroartemisinin-piperaquine had been regularly used in this population for only three years prior. Possible reasons for the rapid decline in dihydroartemisinin-piperaquine efficacy include private sector availability, self-treatment, poor compliance, and the long terminal half-life of piperaquine potentially exposing surviving parasites to subtherapeutic drug levels [5], [24]. In theory, the latter could create a higher risk of resistance due to the shorter terminal elimination half-life seen with the 2 day course, but there was otherwise little pharmacokinetic explanation to favor greater efficacy or risk for resistance for the 2 or 3 day regimen. Piperaquine pharmacokinetic parameters were similar to those reported recently in Southeast Asian adults, with a long terminal elimination half-life and similar C_max_ for the 3-day course in the fed state, and a higher C_max_ as expected for the 2-day course. While calculated AUC was larger for the 2-day course, the AUC in the 3-day course was likely underestimated due to the limited time points available. Short courses of artemisinins alone as part of an ACT regimen are not intended by themselves to be curative, but to rapidly clear initial parasite burden and shorten the febrile period. Because clear pharmacokinetic-pharmacodynamic relationships have not been established for either *P. falciparum* or *P. vivax*, the relative contributions of artemisinin levels to therapeutic outcome of ACTs have yet to be established, and were not measured [25].

Limited sample size, particularly for *falciparum*, permits neither definitive conclusions regarding dihydroartemisinin-piperaquine efficacy nor detailed associations between molecular markers of drug resistance and treatment failures. However, the high proportion of *falciparum* failures, and association with drug levels below parasite piperaquine IC_50_s is concerning. Increased *Pfmdr1* copy number was not associated with first recurrence, supporting prior correlations between *in vitro* piperaquine and chloroquine sensitivity and *Pfmdr1* copy number amplification in Thai-Burmese *falciparum* isolates [26]. Nearly all isolates had the *Pfmdr1* 184F mutation, associated with mefloquine selective pressure in western Cambodia [27], and all isolates had mutant *pfcrt* CVIET haplotype associated with reduced susceptibility to chloroquine and piperaquine [28], [29]. Lack of *pvmdr1* amplification in *vivax* infections, previously associated with decreased chloroquine susceptibility, also suggests possible chloroquine resistance [30].

While piperaquine was relatively well tolerated with few treatment-emergent adverse events beyond those associated with malaria itself, there was a safety signal suggested by the degree of QT interval prolongation. This signal may have been underestimated as EKGs in our study were obtained at 24-hour trough but not peak piperaquine concentrations, which typically occur at 4–6 hours post-dose. A food effect cannot be ruled out as a contributing factor, given that DHA-piperaquine was administered with a low-fat (17g) snack. While substantial cardiac safety signals from DHA-piperaquine were not reported in a prior regulated multi-center clinical trial [6], and there have been no case reports of piperaquine-induced Torsades-de-Pointes, nearly 20% of the 80 subjects dosed had at least a grade 1 adverse event from QT prolongation. Piperaquine has been used in millions of people without reports of life-threatening cardiotoxicity to our knowledge. Prior studies have suggested that it may be difficult to distinguish true drug-induced QT prolongation from the effects of the underlying illness, fever and tachycardia [31] [32]. However the mean prolongations seen here of 20–30 milliseconds over baseline at trough rather than peak post-dose levels were considerably higher than those previously reported, and warrant further investigation. We are currently conducting further detailed investigations regarding this finding (ClinicalTrials.gov - NCT01624337; NCT01849640).

This is the first study to be carried out in a predominantly military population in Cambodia since 1977 [7], [10], [33]. Residing along endemic borders, often in difficult terrain, mobile populations represent a critically important group for malaria containment and elimination efforts. A recent survey of the malaria elimination program in Sri Lanka highlighted the need to target mobile gem miners and military personnel [34]. Similarly in Cambodia, where *Plasmodium* transmission is geographically variable, military personnel may act as a reservoir for malaria transmission to the general population [7], [10], [33]. The high proportion of asymptomatic infections at enrollment and low rates of malaria-free survival over the four months of the cohort study may pose greater challenges for malaria elimination efforts than the evidence for piperaquine resistance. The study highlights limitations of an approach to malaria containment limited to blood stage agents. There is an unmet need in this setting for both transmission-blocking and radical treatment of vivax malaria with primaquine, without which the elimination of *vivax* may be impossible. A key positive finding was that primaquine administered at cohort discharge was safe and well-tolerated even in G6PD deficient individuals, despite a 39% *vivax* six-month recurrence rate. Despite this, the very small number of G6PD-deficient patients treated with primaquine, and diagnostics limited to qualitative fluorescent spot testing does not warrant widespread use of primaquine without testing in this population. Further investigations into primaquine safety in G6PD-deficient individuals are currently underway.

Evidence of reduced dihydroartemisinin-piperaquine efficacy shortly after introduction in Cambodia along with clinical evidence for piperaquine resistance are concerning. Frequent recurrences following effective therapy and a potentially large asymptomatic carrier pool will pose substantial challenges to the possibility of malaria elimination in Cambodia. Novel antimalarial development and improved elimination strategies are urgently needed. In the interim, improvements in case management and patient follow-up offer the best hope for containing drug-resistant malaria.

## Supporting Information

Figure S1 **Pfmdr1 copy number from baseline infection, 1^st^ recurrence and 2^nd^ recurrence.** Pfmdr1 copy number for initial falciparum cases (n = 20 along x-axis) at baseline infection (blue), 1^st^ recurrence (red) and 2^nd^ recurrence (green).
(TIF)

Figure S2 **Symptoms on Day 1 or 2 not reported at baseline according to treatment arm.** Symptoms reported on Day 1 or 2 in patients without symptoms on admission according to treatment arm (top bar, red, 3-day DP regimen; bottom bar, blue, 2-day DP regimen).
(TIF)

Figure S3 **Reported malaria episodes after administration of primaquine in G6PD-deficient versus G6PD-normal individuals.** Over 6-month follow-up in person or by telephone, there was no difference in malaria recurrence between G6PD-deficient (n = 6) and G6PD-normal volunteers (n = 20) (p = 0·19). One vivax recurrence in Month 4 occurred in the same individual who relapsed in Month 1. During Months 5 and 6, there were 3 volunteers with previous vivax recurrences who also had falciparum episodes, and 1 volunteer had falciparum infection in Month 6 with no vivax recurrence after primaquine administration.
(TIF)

Figure S4 **Ex vivo drug susceptibility at day 0 and day of recurrence for falciparum infection.** Black symbols represent IC_50_ of parasites from ACPR patients with mean values displayed below in black, while blue symbols/text represent IC_50_ and mean IC_50_ at baseline respectively of parasites from recurrences. Mean differences in IC_50_ between paired samples are displayed as “Δ = ”.
(TIF)

Table S1 **Pvmdr1 copy number in initial and recurrent vivax parasitemia.**
(DOCX)

Checklist S1 **CONSORT Checklist.**
(DOC)

Protocol S1 **Trial Protocol.**
(PDF)
