# Supplementary figures and images for: Efficacy of Two versus Three-Day Regimens of Dihydroartemisinin-Piperaquine for Uncomplicated Malaria in Military Personnel in Northern Cambodia: An Open-Label Randomized Trial
Source: PLoS One. 2014 Mar 25;9(3):e93138. doi: 10.1371/journal.pone.0093138 (PMC3965521; doi:10.1371/journal.pone.0093138)

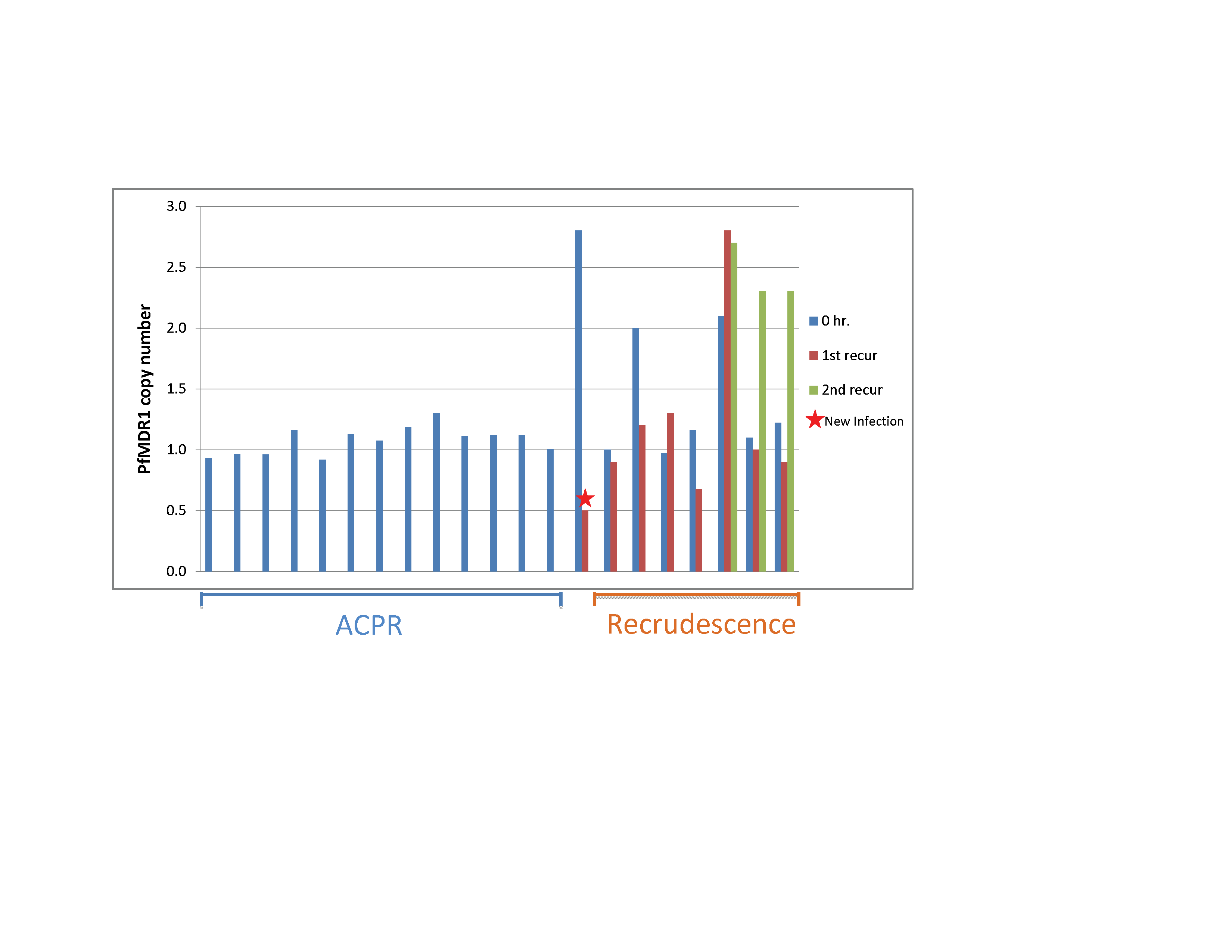

Supplement: Figure S1 — Pfmdr1 copy number from baseline infection, 1st recurrence and 2nd recurrence. Pfmdr1 copy number for initial falciparum cases (n = 20 along x-axis) at baseline infection (blue), 1st recurrence (red) and 2nd recurrence (green). (TIF) [file pone.0093138.s001.tif]

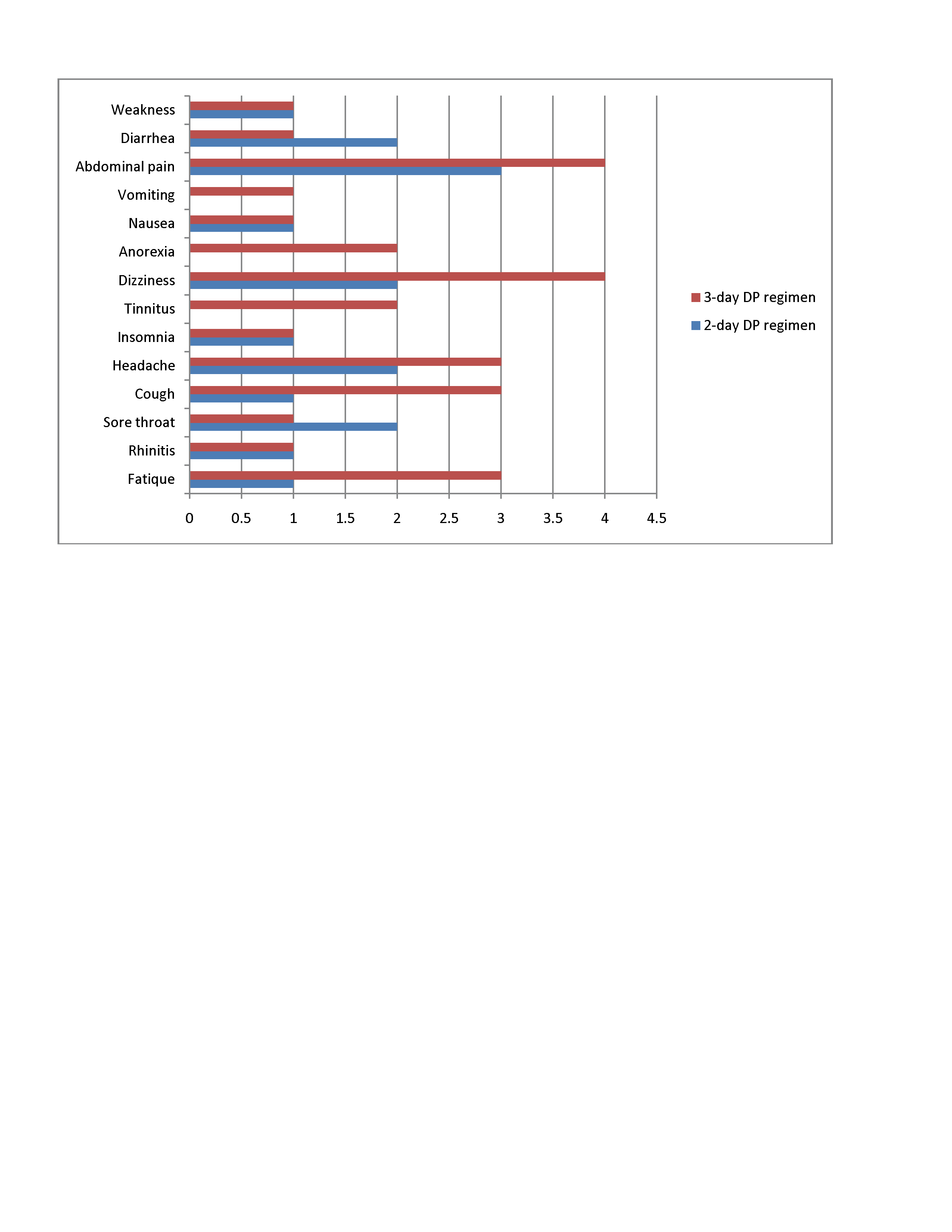

Supplement: Figure S2 — Symptoms on Day 1 or 2 not reported at baseline according to treatment arm. Symptoms reported on Day 1 or 2 in patients without symptoms on admission according to treatment arm (top bar, red, 3-day DP regimen; bottom bar, blue, 2-day DP regimen). (TIF) [file pone.0093138.s002.tif]

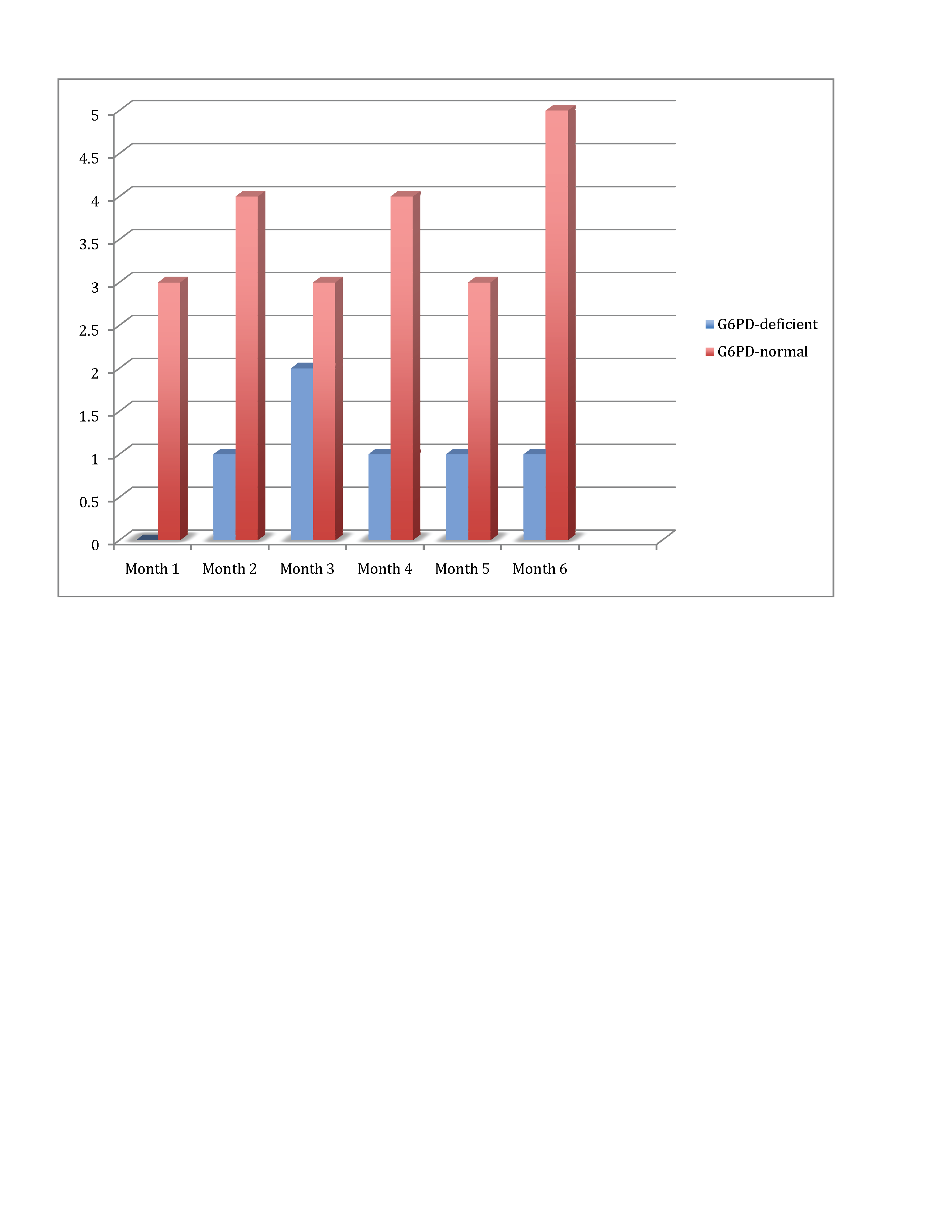

Supplement: Figure S3 — Reported malaria episodes after administration of primaquine in G6PD-deficient versus G6PD-normal individuals. Over 6-month follow-up in person or by telephone, there was no difference in malaria recurrence between G6PD-deficient (n = 6) and G6PD-normal volunteers (n = 20) (p = 0·19). One vivax recurrence in Month 4 occurred in the same individual who relapsed in Month 1. During Months 5 and 6, there were 3 volunteers with previous vivax recurrences who also had falciparum episodes, and 1 volunteer had falciparum infection in Month 6 with no vivax recurrence after primaquine administration. (TIF) [file pone.0093138.s003.tif]

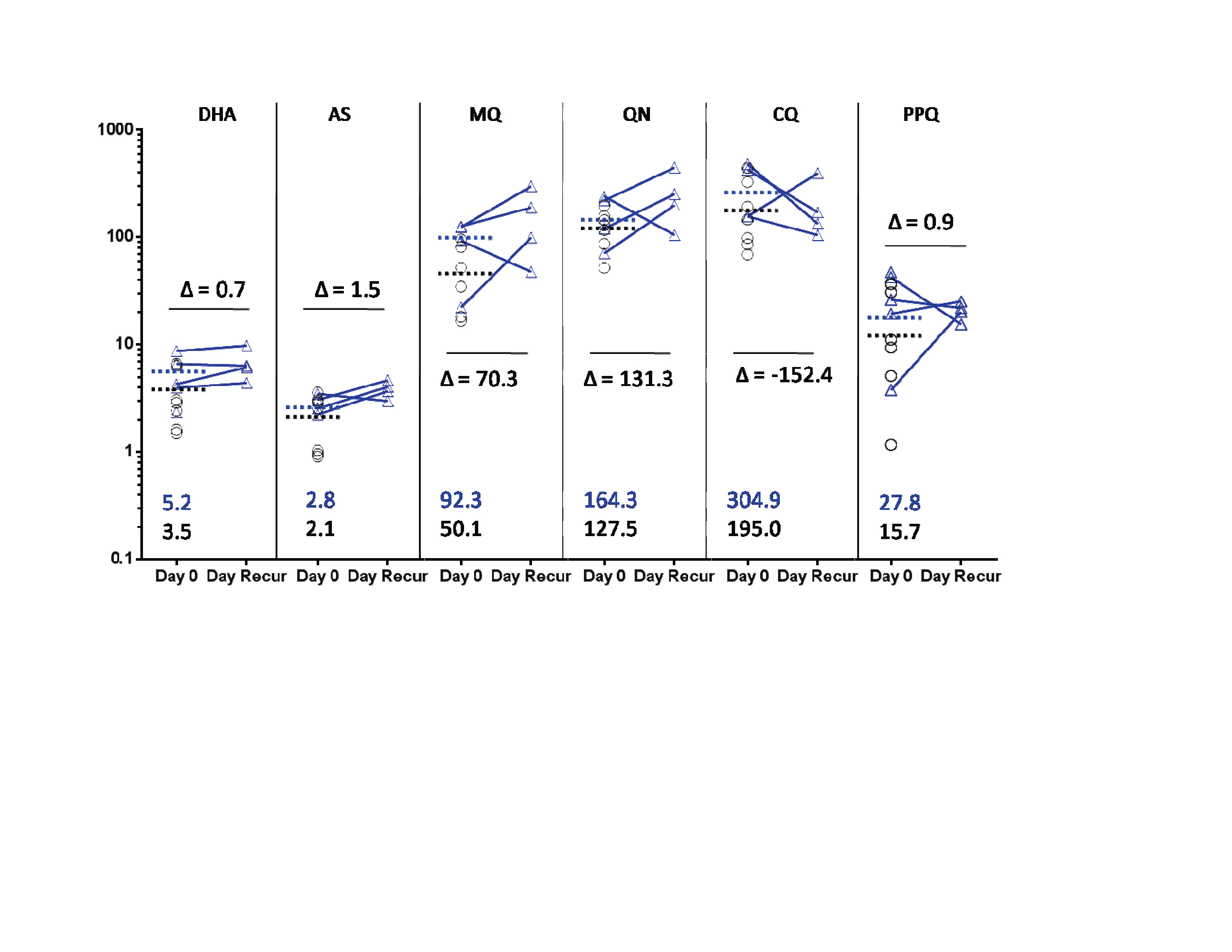

Supplement: Figure S4 — Ex vivo drug susceptibility at day 0 and day of recurrence for falciparum infection. Black symbols represent IC50 of parasites from ACPR patients with mean values displayed below in black, while blue symbols/text represent IC50 and mean IC50 at baseline respectively of parasites from recurrences. Mean differences in IC50 between paired samples are displayed as “Δ = ”. (TIF) [file pone.0093138.s004.tif]
