# Supplementary material for: Efficacy of Two versus Three-Day Regimens of Dihydroartemisinin-Piperaquine for Uncomplicated Malaria in Military Personnel in Northern Cambodia: An Open-Label Randomized Trial
Source: PLoS One. 2014 Mar 25;9(3):e93138. doi: 10.1371/journal.pone.0093138 (PMC3965521; doi:10.1371/journal.pone.0093138)
Supplement: Table S1 — Pvmdr1 copy number in initial and recurrent vivax parasitemia. (DOCX) [file pone.0093138.s005.docx]

**Table S1: *Pvmdr1* copy number in initial and recurrent *vivax*parasitemia**

|  | Initial Parasitemia | First Recurrence | Second Recurrence | Third Recurrence |
| --- | --- | --- | --- | --- |
| Number of Samples^1^ | 49 | 18 | 5 | 1 |
| Number with 2 copies (%)^2^ | 3 (6.1%) | 2 (11.1%) | 0 (0%) | 0 (0%) |
| Average copy number | 1.06 | 1.11 | 1.00 | 1.00 |

^1^: Total samples with genotype determined = 73

^2^: No samples were detected with 3 or more copies of *pvmdr1*
