# Supplementary material for: Efficacy of Two versus Three-Day Regimens of Dihydroartemisinin-Piperaquine for Uncomplicated Malaria in Military Personnel in Northern Cambodia: An Open-Label Randomized Trial
Source: PLoS One. 2014 Mar 25;9(3):e93138. doi: 10.1371/journal.pone.0093138 (PMC3965521; doi:10.1371/journal.pone.0093138)
Supplement: Protocol S1 — Trial Protocol. (PDF) [file pone.0093138.s007.pdf]

An active malaria epidemiology cohort study with evaluation of a 2 day versus 3 day treatment regimen of DHA-Piperaquine for patients with uncomplicated malaria

Short Title: Malaria active epidemiology and treatment study

WRAIR Protocol Number:
WRAIR # 1737
HRPO Log Number A-16251

Principal Investigators:
Chanthap Lon, M.D.
David Saunders, M.D., M.P.H.

Version Number: *Version 1.5*
16 Aug 2010

WRAIR Institutional Review Board (IRB)
Signature Page for Studies Not Exempt Under 32 CFR 219

Principal Investigator Agreement:

1. I agree to follow this protocol version as approved by the IRBs/ERCs.

2. I will conduct the study in accordance with applicable IRB/ERC requirements, Federal regulations, and state and local laws to maintain the protection of the rights and welfare of study participants.

3. I certify that I, and the study staff, have received the requisite training to conduct this research protocol.

4. I will not modify the protocol without first obtaining an IRB/ERC approved amendment and new protocol version unless it is necessary to protect the health and welfare of study participants.

5. (For Greater than Minimal Risk studies or studies of public interest) In accordance with Command Policy 2008-35, I will ensure that the Commanding General receives a pre-brief (or Executive Summary) and approves the study prior to execution.

6. I will ensure that the data (and/or specimens) are maintained in accordance with the data (and/or specimen) disposition outlined in the protocol. Any modifications to this plan should first be reviewed and approved by the applicable IRBs/ERCs.

7. I will promptly report changes to the research or unanticipated problems to the WRAIR IRB immediately via the WRAIR Division of Human Subjects Protection and submit a written report within 10 working days of knowledge of the event.

8. I will prepare continuing review reports at an interval established by the IRB/ERC, and a study closure report when all research activities are completed.

9. I will immediately report to the WRAIR Division of Human Subjects Protection knowledge of any pending compliance inspection by any outside governmental agency.

10. I agree to maintain adequate and accurate records in accordance with IRB policies, Federal, state and local laws and regulations.

____________________________ __________________

Dr. Chanthap Lon Date

____________________________ __________________

Dr. David Saunders Date

Table of Contents

Page

1. PROTOCOL SUMMARY 8
2. KEY ROLES 13
3. BACKGROUND AND RATIONALE 19
   3.1 Introduction 19
   3.2 Development of Antimalarial Chemoprophylaxis 20
   3.3 Malaria Situation in the Royal Cambodian Armed Forces 24
   3.4 Efficacy and Safety of DHA-piperaquine for treatment of uncomplicated malaria 27
   3.5 Efficacy and Safety of Primaquine for radical cure of P. vivax liver stage malaria 30
4. STUDY OBJECTIVES 31
   4.1 Primary Objectives 31
   4.2 Secondary Objectives 32
5. STUDY DESIGN 33
   5.1 Overview 33
   5.2 Endpoints 33
   5.3 Sample Size 35
   5.4 Duration of Volunteer Participation 36
   5.5 Study Group Descriptions and Determinants of Study Group Design 37
   5.6 Population to be Studied 37
   5.7 Study Sites and Selection of the Study Population 37
   5.8 Description of Test Article/Procedure and Placebo: 38
      5.8.1 Packaging and Labeling of the Test Article/Procedure and Placebo 38
      5.8.2 Storage of Test Article 39
   5.9 Monitoring of Clinical Subject Safety 39
6. METHODS 41
   6.1 Recruitment of Study Volunteers 41
   6.2 Informed Consent Process 41
   6.3 Screening Procedures 42
   6.4 Determination of Eligibility 43
      6.4.1 Inclusion Criteria 43
      6.4.2 Exclusion Criteria 43
   6.5 Randomization and Volunteer Assignment 44
   6.6 Blinding 44
   6.7 Administration of Test Article and Placebo (or other Research Intervention) 44
   6.8 Concomitant Medications 45
   6.9 Clinical Assessments 45
   6.10 Specimen (or Data) Collection and Testing 54
      6.10.1 Specimens to be Collected 54
      6.10.2 Specimen Preparation, Processing, Handling, and Storage 54
      6.10.3 Specimen Labeling and Shipment 57
      6.10.4 Specimen Storage and Donation for Future Use 58
   6.11 Data Management 58
      6.11.1 Source Documents 59
      6.11.2 Overview of Case Report Forms 59
      6.11.3 Data Compilation 60
      6.11.4 Disposition of Data 60
   6.12 Adverse Events 60
      6.12.1 Collecting Adverse Events 61

6.12.2 Documenting Adverse Events 62
6.12.3 Expected Adverse Events 62
6.12.4 Serious Adverse Events and Unexpected (Unanticipated) Adverse Events 63
6.12.5 Adverse Event Reporting 64
6.12.6 Follow-up of Adverse Events 65
6.13 Withdrawal Criteria 65
6.14 Rescue Treatment for Malaria Infection Not Responding to Initial Therapy 66
6.15 Criteria for Study Termination 66
6.16 Quality Control and Quality Assurance 67
7. STATISTICAL METHODS 67
7.1 Statistical Procedures 67
7.2 Sample Size Estimation 68
7.3 Randomization and Stratification 69
7.4 Populations for Analysis 69
7.5 Deviations from the Statistical Plan 69
8 DATA ANALYSIS 70
8.1 General Considerations – Data Analysis 70
8.2 Cohort Study Analysis 70
8.3 Efficacy Study Analysis 71
8.3 Pharmacokinetic Analysis 72
8.4 Parasite drug resistance in vitro 73
8.5 Molecular Markers of Parasite Resistance 73
8.6 Safety Analysis 74
8.7 Interim Analysis 74
9. ETHICAL CONSIDERATIONS 75
9.1 Informed Consent 75
9.2 Volunteer Identification and Confidentiality 75
9.3 Risk to Volunteers and Precautions to Minimize Risk 77
9.4 Alternatives to Test Article (or Research Treatment) 77
9.5 Benefits to Volunteers 78
9.6 Risks to Study Personnel and Precautions to Minimize Risk 78
9.7 Risks to the Environment 78
9.8 Financial Incentives to Volunteers 78
9.9 Medical Care for Injury or Illness 78
9.10 Criteria for Discontinuation of Withdrawal of a Volunteer 79
10. ADMINISTRATIVE PROCEDURES 81
10.1 Institutional Review Board and Volunteer Registry Database 81
10.2 Study Medication Accountability 82
10.3 Disposition of Data 82
10.4 Access to Source Data/Documents 82
10.5 Certification of Translation (where applicable) 82
10.6 Protocol Amendments 82
10.7 Protocol Deviations 83
10.8 Publication Policy 84
10.9 Responsibilities of Study Personnel 84
10.10 Responsibilities of the Medical Monitor 85
10.11 Compliance with Laws and Regulations 86
11. REFERENCES 87

Appendix A. Study Drug – Duo-cotecxin Label 91
Appendix B. Malaria Treatment in the Kingdom of Cambodia, Treatment Guideline for Referral Hospital, Health Centre and Health Post in Cambodia 92
Appendix C. Guidance for Industry - Malaria: Developing Drug and Nonvaccine Biological Products for Treatment and Prophylaxis. DRAFT GUIDANCE 92
Appendix D. Recommendations - Cambodia Drug Policy Workshop, PHNOM PENH, 08-09 APRIL 2010 (submitted as an Attachment) 92
Appendix E. Source Documents / Case Report Forms (submitted as an Attachment) 92

List of Abbreviations

ACT	Artemisinin-based Combination Therapy
AFRIMS	Armed Forces Research Institute of Medical Sciences
AR	Attack Rate
AOR	Area of Responsibility
CDC	Center for Disease Control
CFR	Case Fatality Rate or Common Federal Rule
CNM	National Center for Parasitology, Entomology and Malaria Control
CRF	Case Report Form
CRO	Clinical Research Organization
DHA	Dihydroartemisinin
DHSP	Division of Human Subjects Protection
DNA	Deoxyribonucleic Acid
DOT	Directly Observed Therapy
DP	Dihydroartemisinin-piperaquine
ELISA	Enzyme-Linked Immunosorbent Assay
EMEA	European Medices Agency
FDA	Food and Drug Administration
FWA	Federal-Wide Assurance
GCP	Good Clinical Practice
G6PD	Glucose-6-phosphate dehydrogenase
GMP	Good Manufacturing Practice
HR	Heart Rate
HRPO	Human Research Protection Office
USAMRMC	United States Army Medical Research and Materiel Command
IC	Inhibitory Concentration
ICF	Informed Consent Form
ICH	International Conference on Harmonization
IEC	Independent or Institutional Ethics Committee
IPD	In-patient Department
IRB	Institutional Review Board
ITT	Intention-to-Treat
LD	Linkage Disequilibrium
MMV	Medicines for Malaria Venture
MR4	Malaria Research and Reference Reagent Resource Center
MTF	Military Treatment Facility
OPD	Out-patient Department
ORP	Office of Research Protection
QC	Quality control
RBC	Red Blood Cell
P.	*Plasmodium*
PCR	Polymerase chain reaction
Pf.	*Plasmodium falciparum*
Pv.	*Plasmodium vivax*
RCAF	Royal Cambodian Armed Forces
RDT	Rapid Diagnostic Test

RNA	Ribonucleic Acid
ROC	Receiver-operator characteristics
RR	Respiratory Rate
SME	Sponsors Medical Expert
SOP	Standard Operating Procedure
SP	Sulfadoxine-pyrimethamine
SSP	Study Specific Procedure
TOU	Test of Understanding
US FDA	United State Food and Drug Administration
WBC	White Blood Cell
WHO	World Health Organization

1. PROTOCOL SUMMARY

Summary Statement

This is an active observational **Cohort Study** of malaria epidemiology with a nested two arm, randomized, open label **Treatment Study** comparing the efficacy, safety, tolerability and pharmacokinetics of a two versus three day course of Dihydroartemisinin-Piperaquine (DP) for those developing uncomplicated malaria. A 2 day regimen of DP is the current standard of care for RCAF personnel, but RCAF is seeking to develop antimalarial treatment policy based on rigorous locally gathered clinical evidence. At the conclusion of the Cohort Study, a subset of volunteers with documented exposure to Plasmodium vivax during the study will be treated with primaquine as presumptive anti-relapse therapy directed against the exoerythrocytic malaria stages of *P. vivax*, and followed passively for an additional 6 months.

Background and Rationale

Plasmodium falciparum malaria (*Pf*) continues to be a major cause of global morbidity and mortality with 350-500 million cases per year, and over 1 million deaths. Despite containment and control efforts, *Pf* continues to be endemic in areas of Cambodia near the Thai and Lao borders. Populations of civilian and military personnel in the region continue to suffer from an inordinate burden of malaria, particularly in forward deployed areas. A strategy to prevent malaria infection is regarded as a critical element of appropriate force health protection measures. Most militaries in the region do not use antimalarial chemoprophylaxis on a routine basis, relying instead on personal protective measures to prevent mosquito bites. In the Royal Cambodian Armed Forces, this approach has had limitations with an ongoing burden of malaria in some areas.

The US Army Medical Materiel Development Activity, in cooperation with the Walter Reed Army Institute of Research are currently establishing field sites to evaluate new products for antimalarial chemoprophylaxis which are badly needed. While several viable prophylaxis drug candidates exist, no new studies to evaluate the efficacy for a prophylaxis indication have been conducted in more than a decade. This study is the first step in determining the feasibility of conducting malaria prophylaxis studies at this site, and characterizing the population, malaria epidemiology and effectiveness of currently prescribed antimalarial therapy with 2 days of DHA-piperaquine. The purpose of the study will be three-fold:

1. To estimate the malaria attack rate (AR) and thoroughly characterize the population at risk for malaria with an active observational cohort study in order to design Phase 2 and 3 clinical studies of antimalarial chemoprophylaxis.

2. To compare the efficacy (rates of recurrence at 42 days), safety, tolerability, and pharmacokinetics of currently used 2 versus 3 day regimens of DHA-piperaquine (DP) in Volunteers developing malaria infection. This information will be used to advise the Cambodian government, including the Ministry of National Defense and the national malaria control program (CNM) on the appropriate regimen of DHA-piperaquine. This will also characterize the treatment efficacy of DHA-piperaquine in this setting – an essential baseline data element - prior to undertaking further studies on prophylaxis.

3. To estimate the apparent efficacy of primaquine as a presumptive anti-relapse therapy directed against the exoerythrocytic malaria stages of *P. vivax*, by passively following the subset of P. vivax exposed volunteers through telephone contact for an additional 6 months.

DHA-piperaquine is a safe, well-tolerated drug for the treatment of drug resistant malaria, and has a well documented history of safety and effectiveness, particularly in Southeast Asia. Long term use of piperaquine or a piperaquine combination product as a chemoprophylaxis agent is currently being considered for advanced clinical development by the US Army. Establishing the baseline effectiveness of this medication for malaria treatment in this population is the first step in conducting rigorous, carefully controlled clinical research studies. The data obtained in this study may also be used to design studies of the safety and efficacy of other products in advanced clinical development including other drugs, vaccines and personal protective measures.

Objectives

Primary:

1. To define the epidemiology of malaria infection in the RCAF to include the estimated malaria attack rate (AR), patient demographics and health status, severity of illness, parasite phenotypic and genotypic resistance characteristics, and the spectrum of apparent clinical immune status to malaria.

2. To compare the efficacy (based on rates of recurrence at 42 days) of the currently used 2 day regimen, compared to a 3 day regimen of DHA-piperaquine (DP) in Volunteers developing uncomplicated malaria infection.

Secondary:

1. To compare the safety, tolerability, and pharmacokinetics of the currently used 2 day regimen, compared to a 3 day regimen of DHA-piperaquine (DP) in Volunteers developing uncomplicated malaria infection.

2. To advise the Cambodian government, and national malaria control program (CNM) and Ministry of National Defense on the appropriate regimen of DHA-piperaquine for the treatment of uncomplicated malaria.

3. Assess the predictive ability of clinical history, combined with antibody levels against malaria antigens such as merozoite surface protein-1 (MSP-1) to serve as surrogate endpoints of malaria exposure in this population.

4. Estimate the relapse rates of P. vivax in the population at risk, and estimate the rate of relapse following treatment with primaquine as a presumptive anti-relapse therapy.

5. Estimate rates of glucose-6-phosphate dehydrogenase (G6PD) deficiency in the population at risk.

6. Assess the capability of the joint AFRIMS/RCAF/CNM team to support a regulated clinical trial of antimalarial chemoprophylaxis and/or other future interventions of mutual interest.

7. Assess the degree of antimalarial drug resistance in the parasite populations studied by evaluating 42 day rates of malaria recurrence for those Volunteers who develop malaria, and correlating clinical outcomes with pharmacokinetic drug levels, in-vitro drug susceptibility testing and molecular markers of drug resistance.

8. Establish a database of historical control data of malaria attack rates in this population for comparison with results of future studies where the use of placebo is determined to be otherwise unfeasible. The US Food and Drug Administration has proposed comparison with historical cohorts as an alternative strategy where placebo-controlled studies cannot be conducted.

9. Assess the willingness of volunteers, and the Cambodian military to participate in future clinical trials.

Study Design

This is an active observational **Cohort Study** of malaria epidemiology in healthy service members of the Royal Cambodian Armed Forces and their dependents, with a nested two arm, randomized, open label **Treatment Study** comparing the efficacy, safety, tolerability and pharmacokinetics of a two versus three day course of Dihydroartemisinin-Piperaquine (DP) for those developing uncomplicated malaria.

Cohort Study

This is an observational active epidemiology cohort study to determine the malaria attack rate (AR), G6PD status and malaria immune status (based on clinical history and MSP-1 antibody levels) in service members of the Royal Cambodian Armed Forces and their dependents. The results of the study will be used to design pivotal Phase 3 studies of antimalarial chemoprophylaxis for FDA licensure. This study will be a key element of building capacity, and preparing RCAF sites for conducting Phase 2 and 3 clinical trials. Up to 200 Volunteers will be enrolled and followed weekly for an estimated 3-5 month period, depending on the AR. Volunteers who do not complete the study (drop-outs, withdrawals, loss to follow-up) will be replaced. At the conclusion of the Cohort Study, the subset of volunteers with documented exposure to *Plasmodium vivax* malaria during the study will be treated with primaquine as presumptive anti-relapse therapy directed against the exoerythrocytic malaria stages of *P. vivax*, and followed passively for an additional 6 months.

Treatment Study

Volunteers developing malaria infection during the active Cohort Study period will be treated as part of a two arm, randomized, open label treatment study comparing the efficacy (42 day PCR-corrected malaria recurrence rate), safety, tolerability and pharmacokinetics of a two versus three day course of Dihydroartemisinin-Piperaquine (DP). The exact length of follow-up of the cohort will be determined by the number of Volunteers developing malaria to ensure that there is sufficient statistical power (at least 80%) to compare the relative efficacy of the two treatment groups. Volunteers will be followed until up to 80 Volunteers have been randomized to malaria treatment. Based on preliminary epidemiology data and statistical considerations, this will take approximately 3-5 months, depending on attack rates encountered.

Population: Personnel and dependents of the Royal Cambodian Armed Forces stationed in malarious areas in Cambodia.

Primary Endpoint for Cohort Study: Malaria Attack Rate (AR) - Incidence of malaria infection, including recurrent infection, as defined by positive PCR-corrected microscopy diagnosis.

Primary Endpoint for Treatment Study:

Comparative rates of malaria recurrence between the 2 and 3 day DP regimens at 42 days after treatment of malaria infection, diagnosed by positive PCR-corrected malaria microscopy.

Secondary Endpoints

1. Cumulative incidence of slide-proven total and species-specific parasitemia.
2. Estimate of apparent rates of clinical non-immunity to malaria based on medical history.
3. Accuracy of comparative malaria antibody titers to malaria antigens (such as MSP-1) at baseline, post-malaria exposure and at discharge from the study as a surrogate for endpoint for malaria exposure and immunity level.
4. Comparative rates, duration and intensity of treatment-related adverse drug events, and total adverse events in each treatment group.
5. Duration of detectable piperaquine drug levels following treatment with DHA-piperaquine.
6. Species-specific treatment efficacy.

7. Drug resistance against locally used drugs based on patterns of *in vitro* parasite inhibition as measured by concentration at which 50% of growth is inhibited (IC_{50}).

8. Evaluation of QTc interval prolongation using 12-lead EKG measurements during DP treatment and up to 4 weeks after DP treatment completion.

9. 42 day clinical and parasitological cure rates for Volunteers that develop and are subsequently treated for malaria during the study according to current national guidelines

10. Demographic descriptive statistics of the intended study population.

11. Incidence of qualitative G6PD deficiency in the study population

12. Mean/median time to first slide-proven parasitemia following treatment in cases of malaria recurrence.

13. Rates of occurrence and relapse with *P. vivax* malaria during the Cohort Study, and up to 6 months afterward (assessed by telephone contact) for those Volunteers treated with primaquine at study discharge for documented blood stage *P. vivax* infection during the Cohort study.

Inclusion Criteria:

1. Otherwise healthy volunteer, 18-65 years of age, eligible for care at an RCAF facility, and at risk for contracting malaria
2. Able to provide informed consent
3. Likely to reside in endemic area for the duration of the study
4. Available for follow-up for anticipated study duration, and agrees to participate for the duration of the study
5. Authorized by local commander to participate in the study if on active duty

Exclusion Criteria:

1. History of allergic reaction or contraindication to DHA or piperaquine
2. Significant acute comorbidity requiring urgent medical intervention
3. Pregnant or lactating female, or a female of childbearing age who does not agree to use a highly effective method of birth control during the study
4. Clinically significant abnormal EKG, including a QTc interval $\geq$ 500 ms.
5. Judged by the investigator to be otherwise unsuitable for study participation

Study Site:

Multiple sites authorized by the Ministry of National Defense determined to have high incidence rates of malaria based on current estimates by AFRIMS, CNM and the RCAF health services.

Design and Methodology:

Following documentation of informed consent, study Volunteers will have an initial targeted medical history and physical examination performed by a study physician. Prior clinical history of malaria will be assessed by a directed, volunteer completed-questionnaire with a follow-up interview by a provider. Volunteers will then have a clinical assessment including a directed physical examination. Volunteers will have blood drawn for PCR-corrected malaria blood smears, malaria antigen such as MSP-1 antibody titer, CBC, G6PD activity (in case radical cure of *P. vivax* is required), renal function and liver function testing at baseline.

Volunteers will be followed weekly by study personnel with a brief clinical assessment (to include determination of temperature) for the duration of the specified Cohort Study follow-up period. The assessment will include an open-ended, non-leading assessment of adverse events by an investigator or

designee. Volunteers that remain symptom free will have malaria smears performed every month. Volunteers with a clinical suspicion of malaria at routine follow-up, or at any time during the study will have a blood film examination for malaria. If the smear is negative, alternative diagnoses for febrile illnesses will be described as accurately as possible based on available diagnostic capabilities. The observed malaria attack rate (AR) in the active malaria epidemiology cohort will be used to estimate an appropriate sample size and follow up period for potential future antimalarial chemoprophylaxis studies. Species-specific attack rates, including relapses will also be evaluated to provide a detailed epidemiological picture of malaria in the population at risk. Drop out rates and reasons for dropping out will be reported.

Volunteers developing malaria infection during the active observation follow-up period (including enrollment) will be treated as part of a two arm, randomized, open label Treatment Study comparing the efficacy (42 day PCR-corrected malaria recurrence), safety, tolerability and pharmacokinetics of a two versus three day course of Dihydroartemisinin-Piperaquine (DP). All Volunteers with signs or symptoms of malaria discovered at any time during the Cohort Study will be evaluated with malaria microscopy. This may occur during weekly clinical follow-up, monthly routine blood smear (if asymptomatic), or at any time that the volunteer reports symptoms to the study team directly or through a unit liaison. Active detection, rapid diagnosis of suspected malaria cases and prompt treatment will be the highest priority of the Cohort Study.

Volunteers with positive malaria smears will be referred to the Medical Treatment Facility (MTF) for care by the study team. Female volunteers will have a urine pregnancy test, and if positive, they will not receive Test Article, and will be treated under national guidelines for the treatment of malaria in pregnant patients. Female volunteers will also have a urine pregnancy test at the 42 day post-treatment follow-up visit. They will have blood collected for malaria PCR and malaria antigen such as MSP-1 level and a baseline piperaquine pharmacokinetic drug level (necessary to standardize the bio-analytical chemistry analysis). Blood will also be collected for parasite in vitro drug resistance characterization, and analysis of molecular markers of infection. Following the baseline clinical assessment, patients who present with danger signs indicating severe or complicated malaria infection will be treated under current national guidelines for treatment of severe malaria, which currently include the use of intramuscular artemether or intravenous artesunate. Patients assessed as having uncomplicated malaria will be randomized in open label fashion to treatment with either a 2 or 3 day course of DHA-piperaquine (DP) by directly observed therapy (DOT) at the MTF. Patients diagnosed with their first malaria infection will receive a total of 9 tablets containing 40mg DHA and 320mg of piperaquine in divided doses at 0 and 24 hours (4.5 tablets once per day) for the 2 day course, or at 0, 24 and 48 hours (3 tablets once per day) for the 3 day course. Medication compliance for malaria treatment will be assured by directly observed therapy by study personnel during dosing.

During treatment, patients will have vital signs including temperature, blood pressure, pulse and respirations evaluated, and blood drawn for malaria smears at 4 and 8 hours after the first dose of medication, and then every 8 hours until 2 consecutive negative smears are obtained. In addition, blood will be drawn for pharmacokinetic piperaquine levels at 4, 24, 48 and 72 hours post dose, then weekly for 42 days. Blood will be collected again for CBC, parasite *in vitro* drug resistance characterization, and analysis of molecular markers of resistance at 24, 48 and 72 hours after the initiation of treatment. Volunteers will have a 12-lead EKG performed at the time of diagnosis, and then daily until treatment of uncomplicated malaria is complete. If the QTc interval is prolonged over baseline measurement by more than 10 milliseconds at discharge, then 12 lead EKG will be repeated weekly until normalized, or until week 4 post treatment. Patients will be discharged from the MTF at clinical and parasitological resolution of malaria infection (at least 2 consecutive negative smears), and followed weekly until day 42, resuming their assigned weekly follow-up schedule within the Cohort Study.

All Volunteers with suspected recurrent malaria symptoms during the follow-up period of the Cohort Study will be re-evaluated by microscopy, and if positive for malaria will be treated under directly observed therapy based on current national malaria treatment guidelines for Cambodia. If negative for malaria, the patient will be referred for evaluation and treatment of alternative diagnoses to the appropriate RCAF healthcare service providers. Volunteers found to have recurrent malaria after initial re-treatment with first line therapy (which is currently a 3 day regimen of artesunate and mefloquine) will be treated with rescue therapy following current national guidelines (see Appendix B). At the time of writing, this includes treatment with quinine and doxycycline for 7 days. Blood smears following rescue therapy will be collected daily until resolution by 2 negative smears, at least 1 week apart. An active effort will be made to locate Volunteers lost to follow up for any reason during the Cohort and/or Treatment Study for repeat of PCR-corrected malaria smears, and malaria antigen such as MSP-1 levels. At the conclusion of the Cohort Study (as determined by the Investigators based on the number of malaria cases treated during the study), Volunteers

will have blood drawn for PCR-corrected malaria blood smears, malaria antigen such as MSP-1 titer, CBC, renal, and liver function at discharge. The discharge questionnaire will also assess willingness to participate in future clinical trials.

Volunteers who develop primary *P. vivax* during the course of the study or have an apparent relapse from latent liver-stage disease after being treated for *P. falciparum* will be treated with DHA-piperaquine to clear blood stage infection during the cohort follow-up period of the study (Cohort Study). At the completion of the Cohort Study, all volunteers who have had known exposure to P. vivax infection (documented by malaria microscopy) during the Cohort Study will be treated with primaquine 30mg per day for 14 days to eradicate liver stage infection. Volunteers with documented G6PD-deficiency at baseline will be treated with the US CDC's currently recommended regimen for G6PD-deficient patients (45mg of primaquine weekly for 8 weeks). G6PD deficient Volunteers will receive all doses by directly observed therapy by the study team, and have a CBC drawn before the first dose of primaquine is given. CBC will be repeated on day 3 after the first dose, and then again before the second dose is administered (one week after the first dose). Volunteers will clinically significant declines in hemoglobin values will be discontinued from 8-aminoquinoline therapy, and instructed to seek medical attention in the future if they develop signs or symptoms of malaria. Volunteers treated with post-exposure 8-aminoquinoline therapy to eradicate P. vivax liver stages will have ongoing follow-up by the study team in coordination with the Ministry of National Defense Health Department monthly by telephone for 6 months to estimate rates of malaria recurrence. The study team will seek to obtain medical records from the relevant clinical encounters from all Volunteers who report a malaria recurrence to the study team during the 6 month follow-up period. Cumulative incidence of primary *P. vivax* infection and recurrence will be estimated. Clinical history and molecular parasite analysis will be used to determine whether recurrence represents a relapse, or new infection where possible.

2. KEY ROLES

Principal Investigators:

Chanthap Lon, M.D., M.C.T.M.
Armed Forces Research Institute of Medical Sciences (AFRIMS)
No. 2. Kim YI Sung Boulevard

Khan Tuol Kork, Phnom Penh, Cambodia

David Saunders, M.D., M.P.H.
Dept. of Immunology and Medicine
Armed Forces Research Institute of Medical Sciences (AFRIMS)
315/6 Rajvithi Road, Bangkok 10400, Thailand

Associate Clinical Investigators:

Duong Socheat, M.D. MDM, PhD
National Center for Parasitology, Entomology and Malaria Control
Office: #372, Monivong Blvd.
Phnom Penh, Cambodia
P.O. box 1062

Prom Satharath MD
Department of Health,
Ministry of National Defense
Russian Blvd, Phnom Penh, Cambodia

So Mary, M.D.
Chief of military Health officer of Regional 4
Kandek commune, Srong Basat Bakorng,
Siam Reap province, Cambodia

Youry Se, M.D.
Armed Forces Research Institute of Medical Sciences (AFRIMS)
No. 2. Kim YI Sung Boulevard
Khan Tuol Kork, Phnom Penh, Cambodia

Delia Bethell, B.M. BCh, M.A.
Dept. of Immunology and Medicine
Armed Forces Research Institute of Medical Sciences (AFRIMS)
315/6 Rajvithi Road, Bangkok 10400, Thailand

Douglas Walsh, M.D.
Dept. of Immunology and Medicine
Armed Forces Research Institute of Medical Sciences (AFRIMS)
315/6 Rajvithi Road, Bangkok 10400, Thailand

Darapiseth Sea, M.D.
National Center for Parasitology, Entomology and Malaria Control
Office: #372, Monivong Blvd.
Phnom Penh, Cambodia

MAJ Nhim Vanna, Medical Assisstant
Department of Health,
Ministry of National Defense
Russian Blvd, Phnom Penh, Cambodia

MAJ So Samen, Medical Assisstant
Deputy Chief of Military Health Officer of Regional 4
Kandek commune, Srong Basat Bakorng,
Siam Reap province, Cambodia

Sabaithip Sriwichai, RN, Clinical Research Coordinator
Dept. of Immunology and Medicine
Armed Forces Research Institute of Medical Sciences (AFRIMS)
315/6 Rajvithi Road, Bangkok 10400, Thailand

Nillawan Buathong, RN, Clinical Research Coordinator
Dept. of Immunology and Medicine
Armed Forces Research Institute of Medical Sciences (AFRIMS)
315/6 Rajvithi Road, Bangkok 10400, Thailand

Associate Laboratory Investigators:

Stuart Tyner, PhD.
Dept. of Immunology and Medicine
Armed Forces Research Institute of Medical Sciences (AFRIMS)
315/6 Rajvithi Road, Bangkok 10400, Thailand

Paktiya Teja-isavadharm, PhD.
Dept. of Immunology and Medicine
Armed Forces Research Institute of Medical Sciences (AFRIMS)
315/6 Rajvithi Road, Bangkok 10400, Thailand

Phisit Khemawoot, PhD.
Dept. of Immunology and Medicine
Armed Forces Research Institute of Medical Sciences (AFRIMS)
315/6 Rajvithi Road, Bangkok 10400, Thailand

Sathit Pichyangkul, PhD.
Dept. of Immunology and Medicine
Armed Forces Research Institute of Medical Sciences (AFRIMS)
315/6 Rajvithi Road, Bangkok 10400, Thailand

Panita Gosi, PhD.
Dept. of Immunology and Medicine
Armed Forces Research Institute of Medical Sciences (AFRIMS)
315/6 Rajvithi Road, Bangkok 10400, Thailand

Steven R Meshnik
Jonathan Juliano
Jessica Lin
Department Of Epidemiology, CB#7435
Gillings School of Global Public Health
UNC School of Medicine
Chapel Hill, NC 27599

Sponsor:

United States Army Medical and Material Development Activity
Fort Detrick, MD

Sponsor's Medical Expert:

Kevin Leary, M.D.
Pharmaceutical Systems and Medical Affairs
United States Army Medical and Material Development Activity
Fort Detrick, MD

Medical Monitor:

Vansatha Peou , M.D.
Military Hospital Region 4
Srong Basat Bakorng,
Siam Reap province, Cambodia

Kong Phan, M.A.
ICU, Anlong Veng Referral Hospital
Anlong Veng Commune, Anlong Veng District,
Oddormean Chey Province, Cambodia

Clinical Study Monitor:

Suchada Chinaworapong
Quality Assurance, USAMMDA (Contractor)
Armed Forces Research Institute of Medical Sciences (AFRIMS)
315/6 Rajvithi Road, Bangkok 10400, Thailand

Laboratory Coordinators:

Worachet Kuntawanginn
Laboratory Quality Assurance
Dept. of Immunology and Medicine
Armed Forces Research Institute of Medical Sciences (AFRIMS)
315/6 Rajvithi Road, Bangkok 10400, Thailand

Montri Arsanok
Medical Technologist
Dept. of Immunology and Medicine
Armed Forces Research Institute of Medical Sciences (AFRIMS)
315/6 Rajvithi Road, Bangkok 10400, Thailand

Consultants:

Major General Kong Sali
Major Deth Vantha
Department of Health
Ministry of National Defense
Russian Blvd, Phnom Penh, Cambodia

Douglas Tang, Biostatistics
United States Army Medical and Material Development Activity
Fort Detrick, MD

Colin Ohrt, M.D.
Gregory Deye, M.D.
Brian Smith, M.D.
Louis Macareo, M.D.
Alan Magill, M.D.
Walter Reed Army Institute of Research (WRAIR)
503 Robert Grant Avenue
Silver Spring, MD, USA

Koy Lenin, M.D.
Battambang Referral Hospital
Emergency and ICU unit
Dongkorteap village, Tuol Ta Ek Commun,

Battambang district, Battambang province

Institutions engaged in Human Subjects Research:

National Center for Parasitology, Entomology and Malaria Control
#372, Monivong Blvd., Phnom Penh, Cambodia
(FWA# 00010451)

Ministry of National Defense, Royal Cambodian Armed Forces
Department of Health,
Ministry of National Defense
Russian Blvd, Phnom Penh, Cambodia
(FWA# 00014798)

Armed Forces Research Institute of Medical Sciences (AFRIMS)
Department of Immunology and Medicine
315/6 Rajvithi Road, Bangkok 10400, Thailand
(FWA# 00000015)

Laboratories:

Armed Forces Research Institute of Medical Sciences (AFRIMS)
Dept. of Immunology and Medicine
315/6 Rajvithi Road, Bangkok 10400, Thailand

AFRIMS/CNM Reference Laboratory
Battambang Referral Hospital
Dongkorteap Village, Tuol Ta Ek Commun,
Battambang District, Battambang Province, Cambodia

AFRIMS/CNM Field Laboratory
Anlong Veng Referral Hospital
Anlong Veng, Oddormean Cheay Province, Cambodia

University of North Carolina
c/o Steven Meshnick
3113 Hooker Research Building
135 Dauer Dr.
Chapel Hill, NC 27599

Institutional Review Boards:

WRAIR IRB (FWA# 00000015, IRB#00000794)
Walter Reed Army Institute of Research
Division of Human Subjects Protection
503 Robert Grant Ave., Silver Spring, MD 20910-7500

National Ethics Committee for Health Research (NECHR)
Hlth IRB# 1 (FWA# 00010451, IRB # 00003143)
#2 Kim Il Sung Blvd, Khan Tuol Kok, Phnom Penh. Cambodia

University of North Carolina (NHSR Determination)
Office of Human Research Ethics (FWA# 00004801, IRB # 00002074)
Medical School Building 52

Mason Farm Road, CB 7097
Chapel Hill, NC 27599

3. BACKGROUND AND RATIONALE

3.1 Introduction

This is an active observational **Cohort Study** of malaria epidemiology in healthy service members of the Royal Cambodian Armed Forces and their dependents, with a nested two arm, randomized, open label **Treatment Study** comparing the efficacy, safety, tolerability and pharmacokinetics of a two versus three day course of Dihydroartemisinin-Piperaquine (DP) for those developing uncomplicated malaria. The study will be jointly conducted by USAMC-AFRIMS, RCAF and the CNM (the Cambodian national malaria control center). A 2 day DP regimen is the current standard of care for RCAF personnel, but RCAF is seeking to develop antimalarial treatment policy based on rigorous locally gathered clinical evidence. At the conclusion of the Cohort Study, a subset of volunteers with documented exposure to *Plasmodium vivax* during the study will be treated with primaquine as presumptive anti-relapse therapy directed against the exoerythrocytic malaria stages of *P. vivax*, and followed passively for an additional 6 months to estimate malaria relapse rates following presumptive anti-relapse therapy. An important objective of this study is to prepare for future regulated studies of antimalarial chemoprophylaxis in collaboration with the US Army malaria drug development program.

Plasmodium falciparum malaria (*Pf*) continues to be a major cause of global morbidity and mortality with 350-500 million cases per year, and over 1 million deaths. Despite containment and control efforts, *P.f.* continues to be endemic in areas of Cambodia near the Thai and Lao borders. Populations of both civilian and military personnel in the region continue to suffer from an inordinate burden of malaria, particularly in forward deployed areas. A strategy to prevent malaria infection is regarded as a critical element of appropriate force health protection measures. Most militaries in the region do not use antimalarial chemoprophylaxis on a routine basis, relying instead on personal protective measures to prevent mosquito bites (Saunders, unpublished). In the Royal Cambodian Armed Forces, this approach has had limitations with an ongoing burden of malaria in some areas. In the target study population, a high proportion of malaria naïve soldiers and dependents are likely to benefit from antimalarial chemoprophylaxis.

The US Army Medical Materiel Development Activity, in cooperation with the Walter Reed Army Institute of Research are currently establishing field sites to evaluate new products for antimalarial chemoprophylaxis which are badly needed. While several viable prophylaxis drug candidates exist, no new studies to evaluate the efficacy for a prophylaxis indication have been conducted in more than a decade. This study is the first step in determining the feasibility of conducting malaria prophylaxis studies at this site, and characterizing the population, malaria epidemiology and effectiveness of currently prescribed antimalarial therapy with 2 days of DHA-piperaquine. The purpose of the study will be three-fold:

1. To estimate the malaria attack rate (AR) and thoroughly characterize the population at risk for malaria with an active observational cohort study in order to design Phase 2 and 3 clinical studies of antimalarial chemoprophylaxis.

2. To compare the efficacy (rates of recurrence at 42 days), safety, tolerability, and pharmacokinetics of currently used 2 versus 3 day regimens of DHA-piperaquine (DP) in Volunteers developing malaria infection. This information will be used to advise the Cambodian government, including the Ministry of National Defense and the national malaria control program (CNM) on the appropriate regimen of DHA-piperaquine. This will also characterize the treatment efficacy of DHA-piperaquine in this setting – an essential baseline data element - prior to undertaking further studies on prophylaxis.

3. To estimate the apparent efficacy of primaquine as a presumptive anti-relapse therapy directed against the exoerythrocytic malaria stages of *P. vivax*, by passively following the subset of P. vivax exposed volunteers through telephone contact for an additional 6 months.

DHA-piperaquine is a safe, well-tolerated drug for the treatment of drug resistant malaria, and has a well documented history of safety and effectiveness in clinical trials, particularly in Southeast Asia. Piperaquine or a piperaquine combination product as a chemoprophylaxis agent is currently being considered for advanced clinical development by the US Army, but regulated studies of repeated and/or long term use of this product have yet to be completed. Establishing the baseline effectiveness of this medication for malaria treatment in this population is the first step in conducting rigorous, carefully controlled clinical trials. The data obtained in this study may also be used to design studies of the safety and efficacy of other products in advanced clinical development including other drugs, vaccines and personal protective measures deemed mutually beneficial to the collaborating partners.

3.2 Development of Antimalarial Chemoprophylaxis

The development of new, effective drugs for antimalarial chemoprophylaxis that are safe and well tolerated continues to be an important objective of the US Army's drug development program. The target product profile includes weekly dosing, causal (liver-stage) activity, and a very high level of tolerability. The Army continues to plan for weekly-dosed antimalarial chemoprophylaxis trials over the next several years. For the past 2 years, WRAIR, AFRIMS and USAMRU-K have been conducting a thorough search for potential clinical trials sites with appropriate combinations of key factors including host immunity, intensity and duration of malaria exposure, and acceptability of placebo controls. It has been shown previously that performing a malaria prophylaxis study in a population with high levels of semi-immunity to malaria can distort a drug's apparent efficacy (White, 2002; Taylor, 1999). In addition, identified populations will need to have adequate attack rates of *P. falciparum* and/or *P. vivax* (study objective dependent) so that a study's population exposure requirements can be achieved. Ethical considerations, especially with regards to the acceptability of using a placebo arm, are also important as there is no validated surrogate endpoint or biomarker for malaria exposure, despite many years of effort by WRAIR and other groups.

The goal in the search for prophylaxis study sites has been to identify at least two different acceptable populations (preferably 4) to conduct pivotal trials in different geographic areas with different populations as this is generally required for product licensure. In addition, there is a desire to demonstrate drug effectiveness for both short

and long term prophylaxis, and demonstrating this may require additional populations with varying malaria epidemiology. Short term prophylaxis is defined as therapy designed to protect against high malaria transmission for travel of 2 months duration or less. Long term prophylaxis is that which is safe and effective versus moderate to high malaria transmission over a period of at least 2-3 months. A third strategy, sometimes referred to as 'fire and forget' prophylaxis involves giving a loading dose of a long-acting drug over 2-3 days in order to provide protection in a malaria endemic area for up to 1 month. Despite recent conflicts, short-term deployments of US military and government personnel are often short (1-4 weeks duration), making this approach highly advantageous in this setting.

Conducting malaria prophylaxis studies has been made more challenging in recent years by pronouncements made in the Declaration of Helsinki 2000. Avoidance of placebo-control studies where effective alternatives exist, use of drugs only in populations that would benefit, and fair access to products under development provided by manufacturers once they are licensed while well-intentioned, have had the effect of greatly confounding the traditional approach to developing antimalarial prophylaxis. Historically, malaria prophylaxis studies have been carried out in semi-immune populations in endemic areas. While such studies have proven adequate for the purposes of licensure, questions of fair use and access, and the validity of extending protective efficacy measures to non-immune populations have all been raised. An exhaustive 10 year search for validated biomarkers of malaria exposure has failed to yield surrogate endpoints suitable for clinical trial use. Defining benefit to populations has also been challenging, and in many cases subjective. While the US FDA has attempted to support the work of antimalarial drug developers by issuing a formal Guidance, unfortunately, it remains in draft form at present (See Appendix C). However, in current form, it does address the use of placebo controls and states that they are valid in the absence of other comparators. The use of historical controls is also suggested, but it is not entirely clear how practicable this would be, or how acceptable in making licensing decisions.

An appropriate site would have the following characteristics: a GCP experienced staff, experienced PI's, an experienced laboratory with respect to microscopy diagnosis and speciation of malaria, an experienced trial coordinator and an established infrastructure. In addition, knowing the drug resistance trends for the area is desirable. In order to most expeditiously execute a phase II or III clinical trial with as little duplication of effort as possible and to reduce fixed infrastructure costs, WRAIR, AFRIMS and USAMRU-K have been performing a systematic search of likely government, military, academic, and industry clinical sites to identify potential trial collaborators over the past two years. This proposal represents the culmination of that search in Southeast Asia.

In the search for appropriate sites, investigators from WRAIR and its OCONUS labs have made substantial progress among a dwindling group of potential locations. Sites that have been investigated in one form or another have included university populations and tea plantation workers in Kenya, Indonesian soldiers, the Australian army, the French army in Africa, the Peruvian army working on the Peruvian-Colombian border, the Brazilian military, NGO's in Ghana.

<u>AFRIMS systematically evaluated the following sites in FY 09</u> –

a. AFRIMS field sites in Thailand and Cambodia

b. the Thai, Cambodian, Philippine and Bangladesh militaries, and
c. civilian sites in Thailand, Cambodia Viet Nam and the Philippines.

Unfortunately, as of submission of this protocol, development of antimalarial prophylaxis products has been delayed by safety, efficacy or cost concerns, each of which could threaten further progress toward licensure. Therefore, we are proposing an alternative (DHA-piperaquine) that has been shown to be safe and effective in the treatment of both P. falciparum and P. vivax, is registered and used extensively in Cambodia, and is a combination product with dual mechanisms of action both to overcome and prevent development of resistance.

Malaria continues to be a major threat to the readiness of both Cambodian and US Army Forces. US Forces deployed to Afghanistan, as well as forces on short term deployments in Africa and other malarious areas are required to take malaria prophylaxis. There have been several recent cases of soldiers on deployment in these areas that did not take appropriate malaria prophylaxis, and ended up getting severe malaria with near fatal outcome. Likewise, malaria continues to be a significant burden for RCAF soldiers deployed on the Cambodian frontiers.

There is an urgent need for a malaria prophylaxis drug with fewer side effects that can be taken once per week. Current options to prevent malaria in deployed forces are limited. There is no licensed or effective vaccine, or one approaching licensing. Personal protective measures such as insecticides, bed nets and mosquito repellants have only limited effectiveness, and are sometimes impractical, expensive or unavailable. Oral medicines to prevent malaria are highly effective (up to 99% in some settings) in preventing malaria. One of the key problems in developing and fielding effective antimalarials is tolerability. However, in some soldiers they cause side effects that can lead to non-compliance, and in some cases this can affect medical readiness. Side effects in a deployment or combat setting can have serious consequences well beyond the health of the individual soldier.

Despite the US Army's multi-million dollar drug development program, inexpensive oral doxycycline hyclate remains the drug of choice for malaria chemoprophylaxis in soldiers deploying in support of Operation Enduring Freedom to Afghanistan. This is largely because doxycycline is one of three drugs approved by the FDA for the chemoprophylaxis of chloroquine resistant *Plasmodium falciparum* malaria. Although its daily dosing regimen is not as convenient as that of mefloquine, it is perceived to have a more practical safety and tolerability profile, and less drug resistance has been reported. However, the safety and tolerability of long term use of this drug in a large population has not been well studied. Earlier literature reports describing the use of doxycycline as malaria prophylaxis among relatively small populations in clinical trials and field studies describe an adverse event (AE) profile that is significant for gastrointestinal upset and phototoxicity (Ohrt, 1997; Baudon, 1999; Pages, 2002). In particular, the doxycycline hyclate (DH) salt form has been reported to have significant tolerability issues related to a local drop in pH to 3 upon dissolution (Amendola, 1985). This drop in pH can cause gastrointestinal side effects including nausea, emesis, epigastric pain, and occasionally a more serious chemical esophagitis. Alternate dosage forms of doxycycline such as doxycycline monohydrate (DM) and enteric coated doxycycline hyclate (DHEC) are purported to be better tolerated. However, the respective costs of these dosage forms

are generally much greater than the cost of immediate release DH - up to 70 fold (Bryan, 2006). Additionally, these forms of doxycycline have never been compared directly in a controlled study with DH.

Compliance with the few available antimalarial chemoprophylaxis drugs remains problematic. Comparing existing data from the published literature reveals a wide range of adverse event rates. In a careful comparison of doxycycline and mefloquine use among Australian soldiers on a 6 month deployment to East Timor, Kitchener et al (2005) found comparable rates of adverse events (~56%) for both drugs, and 6.8% withdrawals due to mefloquine. Acceptance rates were high for both medications with 94% of those taking mefloquine reporting they would use it again compared to 89% for doxycycline. In a large study in non-immune travelers to sub-Saharan Africa by Schlagenhauf et al. (2003), the tolerability of 4 regimens including chloroquine-proquanil, atovaquone-proguanil, doxycycline hyclate and mefloquine were compared. Adverse events ranged from 32-45%, with rates of more serious adverse events lowest among doxycycline and atovaquone-proguanil (6-7% vs. 12-13% for MQ and C-P). A clinical trial comparing doxycycline with mefloquine by Ohrt *et al.* (1997) in semi-immune soldiers in Indonesia showed very low rates of reported side effects for either drug using directly observed therapy. Overall, there have been 4 well-controlled studies that directly compared rates of withdrawal from mefloquine and doxycycline (0-6% for both drugs) and 2 well controlled studies that reported non-compliance (20-32% for doxycycline vs. 20-22% for mefloquine).

Multidrug resistance is a significant problem in many regions of the world, where most strains of P. falciparum are no longer susceptible to the available anti-malarial compounds. This problem has been well documented in Southeast Asia, and it is predicted that a similar situation will occur in Africa (Wongsrichanalai, 2001 and 2002; Hyde, 2002). Chloroquine, once the first-line defense against malaria in Kenya, is no longer effective because of the evolution of multidrug resistant parasites (Price, 2001; Bloland, 1993; Shretta, 2000). This introduced sulfadoxine-pyrimethamine as the drug of choice, but parasite resistance to this treatment developed quickly as well (Omar, 2001; Mberu, 2000; Khan, 1997). As a result, new candidates for antimalarial chemoprophylaxis are being developed with a view to preventing the development of resistance.

New studies of drugs for malaria prophylaxis have not been conducted in 10 years. This is because properly controlled studies require the use of a placebo. A major scientific effort has been made by the Walter Reed Army Institute of Research to discover ways of doing studies without the need for a placebo group, but to date, there is no reliable way to determine if someone has been exposed to malaria. Therefore, the only way to do studies of malaria prophylaxis remains the use of a placebo control. Long-term prophylaxis studies are ideally done in non-immune volunteers, in areas of high attack rate, and with a placebo control so that the exposure rate is determined. Such study populations are increasingly difficult to identify anywhere in the world. A long-term objective of the US Army has been to identify and validate biomarkers of malaria exposure with a goal of defining an approved surrogate endpoint for clinical trials. Validated markers of exposure, if available and accepted by the US Food and Drug Administration and the global scientific community, would eliminate the need for the placebo control arms in prophylaxis studies. In addition, validation of such markers would aid malaria vaccine research. The detection of antibodies to the sporozoite stage of malaria parasites represents a serological indicator of infection and has been used to

estimate the intensity of malaria transmission in endemic areas. Additionally, the presence of anti-circumsporozoite antibodies has also been documented in non-immune travelers returning from endemic areas after prophylaxis with anti-malaria drugs (Druilhe, 1986; Webster, 1992; Archibald, 1990; Del Giudice, 1987). These results appeared to indicate that anti-CSP antibodies can be utilized as serological markers of exposure in semi-immune populations. Additional studies have demonstrated that sporozoite antibodies can also be used for assessment of malaria exposure in non-immune populations and travelers. Despite these efforts, a recent P. falciparum human challenge study at WRAIR where volunteers were treated with Mefloquine, allowing infection to occur, did not seroconvert on a key biomarker, throwing the entire approach into question (Deye et al., unpublished).

Malaria microscopy determines the primary endpoint in malaria prevention trials currently. It is an imperfect reference standard, however. In 2001, there were difficulties with two pivotal clinical trials. In one case, a Phase III malaria prophylaxis clinical trial failed due to false positive smears. A diagnostics trial was left with an inconclusive result, requiring a re-read of all malaria smears. Both of the problems have lead to significant delay in bringing new products to the market. The US Army has established a Malaria Center of Excellence in Kisumu, Kenya with a goal of improving diagnostic accuracy with a primary objective funding is to ensure valid clinical trials. AFRIMS has established an expert microscopy training program, and several AFRIMS expert malaria microscopists have served as trainers at this center.

The decisions regarding which candidates to test in any potential future clinical trial in Cambodia would be made **by mutual consent of the US Army, CNM and the RCAF** with approval and oversight by the medical research ethics panels of both organizations. An agreement in principle to engage in antimalarial research has been obtained from the RCAF Health Department. Medical research helps not just those who will benefit from new treatments; it also helps raise the standards of care in the system where the research is being conducted. To support research requires intensive training, capacity building, human resources development, infrastructure and improved care guidelines. All of these elements would be provided as part of US Army-RCAF cooperation supported by this proposal.

3.3 Malaria Situation in the Royal Cambodian Armed Forces

The Royal Cambodian Armed Forces (RCAF) was chosen as a population likely to benefit from antimalarial chemoprophylaxis. The RCAF offers several promising sites based on a high incidence of malaria among personnel in select areas, logistical support, and commitment to participate in malaria prophylaxis studies. Perhaps most importantly, AFRIMS has an existing research team on the ground which has been actively conducting malaria research in Cambodia for the past 5 years in partnership with the Cambodian National Center for Parasitology, Entomology and Malaria Control (CNM). This pairing of an experienced investigative team with RCAF institutional support working with an ideal population will be the key to success of Phase 2 and 3 studies of antimalarial chemoprophylaxis.

Preliminary data gathered by CNM-AFRIMS on malaria burden in civilian and military populations along border areas indicates that there are populations with potential to benefit from prophylaxis studies in Cambodia. Malaria typically occurs in seasonal peaks in Cambodia. Data from the Anlong Veng health center in Oddar Meanchey Province in 2008 indicate approximately when the peak season is likely to occur in this area (June – August, with a second peak from October – December). In 2008, there were 676 cases of uncomplicated malaria reported from this health center alone – 54% P. falciparum, 38% P. vivax, and 8% mixed infection. In addition, there were 284 severe cases requiring admission for parenteral therapy and 7 in-hospital deaths reported. However, preliminary data collected in the military population in this area indicates that malaria is less seasonal due to the rotation of troops (Lon et al., unpublished).

While malaria transmission has declined dramatically in Battambang Province (where AFRIMS conducted field studies in the past), rates of uncomplicated malaria remain high in Oddor Meanchey, suggesting that malaria in this province is less 'seasonal' than in Battambang. CNM-AFRIMS teams in the area collected only 13 total cases of malaria (4 Pf, 9 Pv) from patients presenting to health facilities as part of a drug resistance surveillance study. However, in Oddor Meanchey province from September to December 2009, samples were collected from 214 positive malaria cases presenting to health facilities or by active surveillance (60% P.f., 37% P.v., and 4% mixed infections). More than 50% of cases were detected through active surveillance of fever cases by community outreach – of 531 fever cases evaluated, 24.9% had malaria parasites including 51 P.f. cases and 78 P.v. cases. This suggests a population with mixed immunity levels and subgroups with high rates of subclinical infection, as well as a large number of symptomatic individuals presenting to healthcare facilities. Under malaria drug resistance surveillance protocol (WR 1576), G6PD status was also assessed – nearly 10.5% of all malaria patients were G6PD positive by fluorescence testing. This, not surprisingly, is comparable to rates of G6PD deficiency reported elsewhere in the region. However, it is unclear whether the rates in malaria patients reflect that in the general population.

There has been a substantial burden of severe malaria in the regions of interest in recent years. Even when treated with appropriate antimalarial drugs, severe malaria in austere or resource-limited settings in the developing world may be associated with high mortality rates because of diagnostic uncertainties, comorbidities, and complications for which treatment may not be available, such as acute renal failure and acute respiratory distress syndrome. Little has been reported in the peer reviewed literature about the burden of severe malaria in Cambodia. Recently AFRIMS conducted a study of severe malaria at Battambang Referral Hospital 2006-2009 (unpublished). A total of 537 cases were discharged from BRH with a diagnosis of severe malaria infection over the 3.5 year period. Overall mortality was 14%. Two hundred thirty three cases (43.4%) were documented P. *falciparum* infection; 41 (7.6%) were P. *vivax*; 17 (3.2%) were mixed infection with P. *falciparum* and P. *vivax* and 246 (45.8%) were diagnosed as malaria infection but were slide-negative, or a slide was not read and/or reported. Males were predominant (57.9%). Of the 246 smear negative clinical diagnoses, 126 (51.2%) were determined by the investigators to be otherwise compatible with a severe malaria diagnosis under national guidelines after reviewing co-morbidities and hospital course. This left 120 of 246 clinically diagnosed patients (48% of smear negative, or 22% of the total) in question as to exact causes of morbidity. Among the slide-negative patients, 106 (43.1%) were treated with anti-malarial drugs alone, and another 140 (56.9%) were given

combined treatment with an anti-malarial drug and an antibiotic due to suspicion of bacterial co-infection. Malaria prophylaxis is intended to prevent morbidity and most importantly mortality from severe malaria in affected populations.

Western Cambodia continues to have a significant number of severe malaria cases, suggesting that interventions to improve access to early diagnosis and treatment of malaria remain priorities for this region. Diagnostic dilemmas abound, with multiple confounding co-morbidities. The key to preventing mortality remains early detection, treatment and referral where necessary. Malaria prophylaxis is intended to prevent the development of severe malaria, particularly in patients who are malaria naïve. Several factors appear to have contributed to the relatively higher mortality rates among patients who were referred to government hospitals including delayed presentation, delayed admission referral, cultural beliefs, and difficulty accessing health care facilities in this austere setting.

General characteristics of the intended study populations in Cambodia are listed below:

- Ethnic composition: Khmer 98-99% and Vietnamese 1-2 %
- Typical living condition: relatively poor, majority farmers (corn, bean or peanut plantations) and loggers.
- There are two different population groups living in the area:

  a) Long term residents, living in the area for more than 5 years, most of whom own the land they are working on.

  b) New residents who remain a minority but make up a fast-growing segment of the population in many border areas. The majority have moved to border areas in the past few years, coming from other Eastern provinces in Cambodia, (such as Kampong Cham, Takeo, and Kandal Province). New residents mostly make a living in forestry, hunting or as laborers, and in these trades are thought to be the group most affected by malaria. They also have more limited access the health care system and develop severe malaria more frequently than long term residents.
- The average annual income for an individual in Cambodia is approximately 1,800 USD (CIA, 2008).
- Level of education: Mostly primary and secondary school only, but according to official statistics most of the population over 18 years is literate (95%).

Access, availability and cost of medical care

Exact details regarding the military health care system in Cambodia are not provided for security reasons. However, in general, facilities and treatment are comparable to those available in the civilian government-run health sector in Cambodia. Overall, health outcomes have been gradually improving in Cambodia over the past 5 years. The infant mortality rate decreased from 95 per 1,000 live births in 2000 to 66 per 1,000 live births in 2005, and continues a steady decline. The government expenditure on healthcare per capita is roughly $4 (Ministry of Health, 2006).

Access to medical care has gradually improved for individuals at highest risk for malaria, including those who are living far from the health facility, such as traditional forest inhabitants, temporary forest migrants, and new forest settlers. In general, public health centers provide basic medical care for a small charge. There are small use charges - e.g., $0.12 charge per visit for the out-patient department and a $5 charge for in-patient care. Particularly poor patients may be treated free of charge based on means testing. Neither private nor government health insurance is generally available, particularly in rural areas.

For pregnant women antenatal health checks, with tetanus immunization and iron supplements if indicated, are offered monthly free of charge from 7 months of pregnancy. In Battambang and neighboring provinces the proportion of women delivering at a health care facility is 36%. The standard care for malaria pregnancy for uncomplicated P. falciparum malaria is quinine alone for 7 days (30 mg/kg/day in 3 divided doses, but not more than 1800mg = 6 tablets of 300mg) and the combination of artesunate and mefloquine (A+M5) for 2nd and 3rd trimester.

3.4 Efficacy and Safety of DHA-piperaquine for treatment of uncomplicated malaria

DHA-piperaquine is a combination of a potent, rapid acting artemisinin derivative, combined with a long-acting paired 4-aminoquinoline (bis-quinoline), similar to chloroquine. Piperaquine has a terminal half-life of several weeks (Tarning, 2008). It is highly active against chloroquine-resistant *Plasmodium falciparum*, and *P. vivax* (Hung, 2004). Dihydroartemisinin (DHA) is the active metabolite of artesunate and artemether. Between 2003 and 2006, clinical trials on the safety and efficacy of DP in against *P.falciparum* and *P. vivax* malaria were carried out in several countries (Thailand, Myanmar, Laos and Cambodia, Uganda, Rwanda) (Zwang, 2009). In all trials, follow-up was at least 28 days and new infections were distinguished from recrudescences by PCR correction. These studies were recently summarized in a pooled analysis - DP administered as treatment was well tolerated with 1.7% early vomiting. There were less adverse events with DP in children and adults compared to a 3 day regimen of Mefloquine and artesunate with the exception of diarrhea. DP treatment resulted in a rapid clearance of fever and parasitemia. The cumulative PCR genotype corrected efficacy at Day 28 of DP assessed by survival analysis was 98.7% (95%CI 97.6–99.8). DP was superior to the comparator drugs in protecting against both P. falciparum recurrence and recrudescence. There was no difference between DP and Artesunate + Mefloquine for 3 days in treating P. vivax co-infections and in suppressing the first relapse. In summary, in a pooled analysis of more than 3,547 uncomplicated malaria patients (1,814 on DP), DP was safe and highly effective. The evidence, including a long period of post-treatment prophylaxis reported in several trials suggests that piperaquine alone or in combination with DHA could serve as a highly effective prophylaxis drug, particularly in settings of drug resistance where combination therapy is desirable (Jansenns, 2007; Zwang, 2009).

Current national treatment guidelines for malaria in Cambodia are included in Appendix B. For uncomplicated malaria, a 3 day course of oral artesunate and mefloquine combination therapy is recommended. However, recent results suggest the efficacy of this combination is declining (Wongsrichanala, 2008), and that tolerance to the artemisinin

component may be a factor (Noedl, 2008). A randomized open-label non-inferiority study comparing the efficacy of 3 days of DP vs. 3 days of artesunate and mefloquine (A+M3)in 464 Cambodian patients found that PCR-adjusted cure rates on day 63 were nearly identical at 97.5% for both DP and A+M3 (Janssens, 2007). DP was better tolerated - vomiting, dizziness, palpitations, and sleep disorders were all more commonly reported in the A+M3 group, consistent with the side-effect profile of mefloquine.

The optimal dose for DP as a prophylaxis medication has yet to be defined. Several options for dosing are potentially available. A recent study on the Thai-Burmese border found that a 3 day loading dose of DP (basically a standard treatment dose), was protective for one month, and up to 6 months when repeated (Nosten, unpublished, personal communication). Thus, in this intermittent preventive treatment mode, DHA-piperaquine might indeed serve as a highly useful 'fire and forget' prophylaxis agent for short term deployments. In addition, it is likely to be effective in weekly dosing mode. While the potential for use of DP or piperaquine alone as prophylaxis appears promising, carefully controlled studies to determine the safety of DP in either weekly or monthly dosing for long term use have not been conducted to date. Gathering basic evidence on therapeutic efficacy and safety of treatment doses in this setting is therefore essential before undertaking regulated studies for a malaria prophylaxis indication.

A formulation of DP known as Eurartesim® (dihydroartemisinin-piperaquine), has been submitted to the EMEA for regulatory approval by Sigma-tau Italy and MMV. DP has been found to be highly effective against *P. falciparum* malaria in adults and children, has a simple dosing regimen (only 3 administrations over 3 days) compared to artemether lumefantrine (Coartem) – the current global standard ACT. In addition, DP has been shown to offer greater protection against new infections than other ACTs, for at least 2 months after treatment. The regulatory dossier submitted comprises data from large clinical trials that involved over 2,700 patients in Africa and Asia of whom 1,600 were children under 5 (MMV press release, July 2009).

In addition the Eurartesim product which has not yet been granted EMEA licensing approval as of writing, several non-GMP forms of this combination product are available from manufacturers in China. Duo-cotecxin and Artekin are brand names for two of the products that are available in Cambodia, and used widely by RCAF personnel. In addition, Artequik, a similar combination of artemisinin and piperaquine at the same dosage strength but over 2 days has become widely used among RCAF personnel. There is currently no standardization, despite the weight of evidence from the published literature suggesting that 3 days of therapy is superior to 2 days, even when the same cumulative dose is given (Krudsood, 2007, others). This is likely due to the short half life of the artemisinin component which limits exposure to only one parasite lifecycle when given over 24 hours, compared to 2 life cycles when given over 48 hours. One of the major objectives of this study will be to determine a detailed estimate of therapeutic efficacy of DP and establish the optimal timing of drug administration in this setting.

Drug resistance is a threat to the success of DP as both a prophylaxis and treatment drug, particularly in Southeast Asia. Currently the existence of artemisinin resistance as well as the potential impact and strategies to overcome artemisinin resistance are much-discussed issues. Suspected clinical artemisinin resistance was reported from Thailand, India, and Sierra Leone as early as the late 1990s (Luxemburger et al. 1998; Gogtay NJ,et al. 2000; Sahr et al. 2001). Isolated *in vitro* resistance has been reported from

numerous countries, particularly in and around Southeast Asia, and artemisinin-resistant strains have been obtained in laboratories by intermittently exposing malaria parasites in culture to rising drug concentrations (Meshnick et. al. 2002). However, the significance of many studies reporting artemisinin resistance is questionable as most reports are limited to either clinical or *in vitro* data.

Detecting and quantifying drug resistance in its early stages requires a combined *in vivo* - *in vitro* strategy. The only reasonable approach is a careful analysis of clinical treatment response parameters, including cure rates, combined with *in vitro* drug sensitivity data. The latest generation of *in vitro* assays is highly sensitive and permits the testing of almost any fresh *Plasmodium falciparum* sample directly from the patient, without major selection bias, cryopreservation, or pre-culturing, procedures that could significantly alter the intrinsic drug sensitivity pattern. One of the biggest problems in distinguishing artemisinin resistance from resistance to the partner drugs is the fact that artemisinins are generally used in combination with drugs that have a longer half life, as will be the case in this study. However, detecting parasite resistance to these drugs *in vitro*, and searching for molecular markers of resistance in those isolates with apparent phenotypic resistance will be important to interpreting the apparent clinical efficacy of DP.

The efficacy endpoint for this study is combined for both species, and the primary endpoint efficacy calculation will be for both species. If 38 subjects are treated in each arm, there will be ≥ 80% power to detect a difference of 23% between the 2 and 3 day treatment regimens. While the first line ACT for the civilian population in Cambodia has been artesunate-mefloquine for the past 5 years, DHA-piperaquine was recommended as the first line therapy for Zone 1 (an area spanning both sides of the western Cambodia-Thai border with high levels of drug treatment failure). The Cambodian military has been using DP for several years as the first line ACT of choice.

While much of the literature suggests the superiority of 3 days DP over 2, the military continues to use 2 days of therapy. The purpose of the study is to establish a baseline therapeutic efficacy of DHA-piperaquine in this setting, as it is actually used. Since the military has only very limited ability to distinguish P. falciparum and P. vivax infection, nearly all infections are treated with DHA-piperaquine. In fact, due to limited diagnostic capabilities in Cambodia as a whole and the difficulty in distinguishing P. falciparum, P. vivax and mixed infections by light microscopy, it was recommended at a recent expert panel meeting on malaria treatment guidelines organized by the WHO in April 2010 that all malaria be treated with an artemisinin combination therapy (ACT). This simplified treatment paradigm was advocated not only to obviate the diagnostic dilemma, but also because recent unpublished data shows that P. vivax chloroquine resistance is as high as 20% at selected sentinel sites. Therefore universal treatment of malaria with an ACT has been proposed to ensure adequate treatment of all malaria and prevention of relapse, and prevention of resistance. In addition, based on the panel's recommendations, it is likely that DHA-piperaquine will become the first line ACT for treatment of uncomplicated malaria throughout Cambodia as early as 2011. A summary of the recommendations from the meeting is attached (see Appendix D). This study is needed to provide high quality clinical evidence to support these impending policy changes.

This population currently uses a two day regimen. It is clearly ethical to establish the effectiveness of these two regimens in this setting and population – both regimens are currently used, but high quality data in this population is lacking. While data from

elsewhere is suggestive, it is clearly not definitive, and there have been no head to head clinical trials comparing the two regimens in this population. Emerging multi-drug resistance in Cambodia is a concern and defining adequate regimens a critical and ongoing need in this dynamic setting. Issues of compliance must also be considered and their impact, particularly in a setting of a drug resistance threat should not be underestimated. In general shorter regimens are easier to comply with, and if a two day regimen were found to be adequate, this would be an important tool for malaria control, particularly in austere settings where medical supervision of therapy is not available. It is also important to note that the study has been designed to compare only the number of days of administration as the cumulative dose of medication in both regimens is identical. For all of the above reasons, clinical equipoise in this setting clearly still exists.

3.5 Efficacy and Safety of Primaquine for radical cure of P. vivax liver stage malaria

Primaquine is an 8-aminoquinoline used for the prevention of relapse of P. vivax malaria due to its relatively unique activity against both liver stage merozoite and particularly hypnozoites forms. However, establishing the efficacy of this treatment is confounded by several factors including unsupervised therapy, reports of parasite tolerance and the possibility of reinfection (Goller et al., 2007).

Primaquine failure is defined by the WHO as a confirmed positive blood smear for P. vivax after treatment with an effective blood schizonticide medicine and primaquine therapy during the follow-up phase in a patient in whom reinfection has been prevented. This follow-up phase varies in the literature from 3 to 12 months. Follow-up should be adapted to the regional characteristics of the parasite (WHO, 2009). Relapses may occur as early as 16 days and as late as up to 3 years after the initial infection, even if the blood stage was adequately treated. In tropical strains relapses tend to occur within the first few months after initial blood stage infection (Baird, Hoffman, 2004). There were high rates of apparent P. vivax relapse in patients treated for apparent P. falciparum blood stage mono-infection in recent trials of artesunate monotherapy in Cambodia conducted by AFRIMS (Noedl et al, unpublished).

Because of the potential for hemolytic anemia induced by primaquine in G6PD-deficient patients, current national guidelines in Cambodia provide for the use of primaquine only where laboratory screening tests for G6PD area available (see Appendix B). However, in practice such tests are not available, resulting in a de facto absence of primaquine use. The CNM, Cambodia is currently interested in determining the usefulness of primaquine for both the radical cure of *P. vivax* infections and the prevention of relapse in patients treated for blood stage P. vivax or with potential P. vivax exposure, as part of a campaign for malaria elimination. The results of this study will be an important component of that decision-making process.

The US Centers for Disease Control and Prevention currently recommends primaquine in a dose of 30mg base per day for 14 days for the prevention of relapse of Plasmodium vivax malaria. For persons with borderline G6PD deficiency or as an alternate to the above regimen, primaquine may be given 45 mg orally one time per week for 8 weeks. Primaquine should not be used during pregnancy. (CDC, 2009) Cambodia is currently considering adoption of primaquine therapy for radical cure using US CDC guidelines

(see Appendix D), and this study will provide valuable information regarding the safety and efficacy of this approach.

Weekly doses of 45 mg of primaquine and 300 mg of chloroquine did not cause significant hemolysis in G6PD deficient African-Americans (Alving et al., 1960). A study in Myanmar (n=22), found that primaquine 45 mg single dose to treat P. falciparum gametocytes and 45 mg weekly x 8 weeks for radical cure of P.vivax malaria was safe and effective in G6PD-deficient volunteers (Kyaw et al., 1994). Additional studies on the use of primaquine in malaria-infected patients with G6PD deficiency in Thailand and Japan showed that the drug was safe and effective without evidence of hemolytic anemia (Charoenlarp et al., 1972). Despite this, other studies have reported that serious hemolytic reactions can occur with small doses and also with even single doses of primaquine 45 mg (Ziai et al., 1967; Reeve et al., 1992).

A key objective of this study will be to estimate the relapse rates of P. vivax in the population at risk, and estimate the rate of relapse following treatment with primaquine as a presumptive anti-relapse therapy. In addition, determining if current US CDC guidelines are appropriate for use in the Cambodian population is important, not only in G6PD normal patients where numerous published studies indicate that the risk is extremely low, but also in G6PD-deficient patients using doses that appear to be safe based on the limited evidence available. Unless Cambodia can develop a malaria elimination strategy that includes anti-relapse therapy for G6PD-deficient patients, success cannot be assured.

There is currently no proven laboratory method to distinguish relapse of malaria (which by definition can occur only with P. vivax), recrudescence and reinfection though experimental methods are being developed. In the present study, genetic analysis of parasites obtained from infections during the active period of Cohort and/or Treatment follow-up periods will be undertaken by the AFRIMS molecular parasitology lab and collaborators at UNC in an effort to define parasite populations and attempt to distinguish parasite populations found at recurrence with those found during primary infection. At this early stage, estimation of P. vivax recurrences is exploratory only and will be done by passive solicitation of symptoms, retrieval of medical records and instructions to treating physicians should subjects treated with primaquine under this protocol end up developing malaria recurrence. It is expected that in some cases, subjects will be rotated out of malarious areas, and be geographically isolated from the possibility of reinfection.

4. STUDY OBJECTIVES

4.1 Primary Objectives

1. To define the epidemiology of malaria infection in the RCAF to include the estimated malaria attack rate (AR), patient demographics and health status,

severity of illness, parasite phenotypic and genotypic resistance characteristics, and the spectrum of apparent clinical immune status to malaria.

2. To compare the efficacy (based on rates of recurrence at 42 days) of the currently used 2 day regimen, compared to a 3 day regimen of DHA-piperaquine (DP) in Volunteers developing uncomplicated malaria infection.

4.2 Secondary Objectives

1. To compare the safety, tolerability, and pharmacokinetics of the currently used 2 day regimen, compared to a 3 day regimen of DHA-piperaquine (DP) in Volunteers developing malaria infection.

2. To advise the Cambodian government, and national malaria control program (CNM) and Ministry of National Defense on the appropriate regimen of DHA-piperaquine for the treatment of uncomplicated malaria.

3. Assess the predictive ability of clinical history, combined with antibody levels against malaria antigens such as merozoite surface protein-1 (MSP-1) to serve as surrogate endpoints of malaria exposure in this population.

4. Estimate the relapse rates of *P. vivax* in the population at risk, and estimate the rate of relapse following treatment with primaquine as a presumptive anti-relapse therapy.

5. Estimate rates of glucose-6-phosphate dehydrogenase (G6PD) deficiency in the population at risk.

6. Assess the capability of the joint AFRIMS/RCAF/CNM team to support a regulated clinical trial of antimalarial chemoprophylaxis and/or other future interventions of mutual interest.

7. Assess the degree of antimalarial drug resistance in the parasite populations studied by evaluating 42 day rates of malaria recurrence for those Volunteers who develop malaria, and correlating clinical outcomes with pharmacokinetic drug levels, in-vitro drug susceptibility testing and molecular markers of drug resistance.

8. Establish a database of historical control data of malaria attack rates in this population for comparison with results of future studies where the use of placebo is determined to be otherwise unfeasible. The US Food and Drug Administration has proposed comparison with historical cohorts as an alternative strategy where placebo-controlled studies cannot be conducted.

9. Assess the willingness of volunteers, and the Cambodian military to participate in future clinical trials.

5. STUDY DESIGN

5.1 Overview

This is an active observational **Cohort Study** of malaria epidemiology in healthy service members of civilian and the Royal Cambodian Armed Forces, with a nested two arm, randomized, open label **Treatment Study** comparing the efficacy, safety, tolerability and pharmacokinetics of a two versus three day course of Dihydroartemisinin-Piperaquine (DP) for those developing uncomplicated malaria. The results of the Cohort Study, particularly the malaria attack rate, will be used to design pivotal Phase 3 placebo-controlled studies of antimalarial chemoprophylaxis for FDA licensure, and the exercise will be a key element of building capacity, and preparing RCAF sites for conducting Phase 3 studies. Up to 200 evaluable volunteers will be enrolled and followed weekly for an estimated 3-5 month period in the Cohort Study. Volunteers who do not complete the Cohort Study (drop-outs, withdrawals, loss to follow-up) will be replaced.

Volunteers developing malaria infection during the active observation follow-up period will be treated as part of a two arm, randomized, open label Treatment Study comparing the efficacy (42 day PCR-corrected malaria recurrence), safety, tolerability and pharmacokinetics of a two versus three day course of Dihydroartemisinin-Piperaquine (DP). A 2 day regimen of DP is the current de facto standard of care for RCAF personnel, but RCAF is seeking to develop antimalarial treatment policy based on rigorous locally gathered clinical evidence. Results from the Treatment Study will be used to guide policy decisions on optimal antimalarial treatment for RCAF personnel. The exact length of follow-up of the Cohort Study will be determined by the number of volunteers developing malaria and referred to the Treatment Study to ensure that there is enough power to compare the relative efficacy of the two treatment groups. Volunteers in the Cohort Study will be followed until up to 80 volunteers have been randomized to uncomplicated malaria treatment with a goal of 76 evaluable volunteers randomized to treatment to ensure at least 80% statistical power to detect a difference in treatment outcomes. Based on preliminary epidemiology data and statistical considerations, this will take approximately 3-5 months, depending on the actual attack rates encountered.

At the conclusion of the Cohort Study, a subset of volunteers with documented exposure to Plasmodium vivax during the study will be treated with primaquine as presumptive anti-relapse therapy directed against the exoerythrocytic malaria stages of *P. vivax*, and followed passively for an additional 6 months.

5.2 Endpoints

The study includes two separate components which are interlinked – an observational epidemiology Cohort Study, and a nested randomized open-label comparison of a two-day versus 3 day regimen of DHA-piperaquine (Treatment Study). Volunteers who develop malaria during the Cohort Study will be treated as inpatients in the Treatment Study. Once they have recovered, they will continue to be followed with the remaining observational cohort until discharged. The purpose of continued follow up is to accurately assess rates of malaria recurrence in the overall cohort.

Primary Endpoint for Cohort Study: Malaria Attack Rate (AR) - Incidence of malaria infection, including recurrent infection, as defined by positive PCR-corrected microscopy diagnosis.

Primary Endpoint for Treatment Study:

Comparative rates of malaria recurrence between the 2 and 3 day DP regimens at 42 days after treatment of uncomplicated malaria infection, diagnosed by positive PCR-corrected malaria microscopy.

Secondary Endpoints

1. Cumulative incidence of slide-proven total and species-specific parasitemia.
2. Estimate of apparent rates of clinical non-immunity to malaria based on medical history.
3. Accuracy of comparative malaria antigen such as Merozoite Surface Protein (MSP-1) titers at baseline, post-malaria exposure and at discharge from the study as a surrogate for endpoint for malaria exposure and immunity level.
4. Comparative rates, duration and intensity of treatment-related adverse drug events, and total adverse events in each treatment group.
5. Duration of detectable piperaquine drug levels following treatment with DHA-piperaquine.
6. Species-specific treatment efficacy.
7. Drug resistance against locally used drugs based on patterns of *in vitro* parasite inhibition as measured by concentration at which 50% of growth is inhibited (IC_{50}).
8. Evaluation of QTc interval prolongation using 12-lead EKG measurements during DP treatment and up to 4 weeks after DP treatment completion.
9. 42 day clinical and parasitological cure rates for Volunteers that develop and are subsequently treated for malaria during the study according to current national guidelines
10. Demographic descriptive statistics of the intended study population.
11. Incidence of qualitative G6PD deficiency in the study population
12. Mean/median time to first slide-proven parasitemia following treatment in cases of malaria recurrence.

13. Rates of occurrence and relapse with *P. vivax* malaria during the Cohort Study, and up to 6 months afterward (assessed by telephone contact) for those Volunteers treated with primaquine at study discharge for documented blood stage *P. vivax* infection during the Cohort study.

5.3 Sample Size

Cohort study:

Up to 200 Volunteers will be enrolled and followed weekly for an estimated 3-5 month period. Volunteers who are withdrawn, drop out, or are lost to follow-up may be replaced with additional Volunteers to ensure the goal of 200 subjects completing the cohort. The major purpose of the Cohort Study follow-up is to estimate the malaria attack rate (AR). An evaluation of the effect of the number of persons followed (N) on the precision (margin of error) of the resulting estimated AR was calculated. Based on preliminary data collected on potential sites, the resulting expected AR estimate from a follow-up period of 4 months is 40%. With a cohort N = 200, then the margin of error under the assumption of an attack rate of 40% is 6.79%. In this case the 95% CI (normal distribution approximation) of the AR estimate would be 40% ± 6.79% (33.21% to 46.79%)].

Treatment study:

Volunteers developing malaria infection during the active observation follow-up period will be treated as part of a two arm, randomized, open label treatment study comparing the efficacy (42 day PCR-corrected malaria recurrence), safety, tolerability and pharmacokinetics of a two versus three day course of Dihydroartemisinin-Piperaquine (DP). The primary endpoint in each of the two treatment regimens is the reoccurrence of malaria (42 days). In prior published studies, using DP in Southeast Asia, cure rates of the two regimens are estimated to be approximately 75% (2 day) and 98 % (3 day) respectively. Based on Fisher's exact test [nominal type-one error rate of 5% (two-sided] a sample size 38 persons per arm (76 total) will provide the required nominal power of 80 % to detect the 23 percentage point difference (98% - 75%) in cure rates [exact power = 81%, exact type one-error = 3%, PASS (2005))]. The exact length of follow-up of the prospective epidemiology cohort will be determined by the number of Volunteers developing malaria to insure the required 80% power to compare the relative efficacy of the two treatment groups.

Early epidemiologic data gathered from existing passively collected government sources estimates that it will take up to 4 months for 40% of Volunteers enrolled (n = 0.40 x 200 = 80) to develop clinical malaria, and be treated by the study team using an active case detection and treatment approach. The required 80% power will be reached when 76 Volunteers have been randomized to uncomplicated malaria treatment (38 Volunteers per group). Therefore, Volunteers in the Cohort Study will be followed until up to 80 Volunteers have been randomized to uncomplicated malaria treatment (40 per group), which allows for two dropouts per group. Based on preliminary epidemiology data and statistical considerations, this will take approximately 3-5 months, depending on attack rates encountered. With N = 200 subjects completing the cohort study and assuming an expected four month cumulative attack rate of 40% (50% five month), the probability of enrolling at least 76 cases within 5 months is > 0.99 (based on binomial distribution). If

the attack rates is substantially lower than expected, (assuming at least a 30% cumulative attack rate over 4 months, or 37.5% over five months), the probability of having to follow Volunteers for more than 5 months to enroll at least 76 malaria patients is 0.53. If the total number of cases enrolled in five months is at least 70 then the power will be ≥75%. If the number of cases is at least 66, power will be ≥ 71%.

5.4 Duration of Volunteer Participation

As noted above in section 5.3, study duration for the prospective cohort period will be determined by the malaria attack rate, following all Volunteers initially enrolled until a cumulative attack rate of approximately 40% is observed. In the target study population of Volunteers with little or no malaria immunity, attack rates are thought to be at the high end of this estimate. Variability is uncertain at this time, and difficult to predict without first conducting a prospective cohort study. Therefore, we estimate that it will take approximately 4 months for 40% of Volunteers enrolled (n=80) to develop clinical malaria, and be treated by the study team using an active case detection and treatment approach.

Those Volunteers that remain negative for malaria throughout the study will be discharged from the study as soon as is practicable under field conditions once the desired attack rate has been observed. Volunteers who become positive for malaria will be treated under national guidelines with directly observed therapy (DOT). Volunteers with more than 42 days remaining in the original Cohort Study will resume the follow-up schedule from the Cohort Study once discharged from the MTF. Volunteers with fewer than 42 days remaining in the Cohort Study at MTF discharge will have follow-up extended to the completion of the Treatment Study – a minimum of 42 days after treatment is started. Volunteers that have a malaria recurrence during the 42 day treatment follow-up period will be re-treated under national guidelines as outlined above, and have weekly follow-up extended until they have clinical resolution of symptoms, and 2 negative blood smears at least one week apart.

Volunteers already treated for malaria initially under the protocol who subsequently develop primary blood stage P. vivax or who have an apparent relapse from latent liver-stage disease will be treated according to current Cambodian national malaria treatment guidelines (currently a 3 day course of chloroquine) to clear blood stage infection during the cohort follow-up period of the study (Cohort Study). Once the cohort follow-up period of the study has been completed, all Volunteers who have had known exposure to *P. vivax* infection will be treated with primaquine 30mg per day for 14 days to eradicate liver stage infection. Volunteers with known G6PD-deficiency will be treated with the currently recommended regimen for G6PD-deficient patients with 45mg of primaquine weekly for 8 weeks with ongoing follow-up by the study team. Therefore, clinical follow-up for G6PD-normal patients by the study team will be extended for 14 days after discharge from the Cohort Study, and extended for 8 weeks for the G6PD-deficient patients to complete DOT of primaquine. Once clinical follow-up is completed, all Volunteers treated with primaquine for *P. vivax* radical cure will be followed by the study team in coordination with the Ministry of National Defense Health Department monthly by telephone, clinic visit, and/or home visit as appropriate for 6 months to estimate rates of malaria recurrence with instructions to contact the study team if malaria-like symptoms are encountered. All volunteers in the study will be provided with a malaria alert card at enrollment. Volunteers who report symptoms of malaria will be referred for consultation with an available study

physician, or if logistically unfeasible, will have their medical records reviewed by the study team as soon as practicable, using a structured interview form (see Appendix E. Case Report Forms).

5.5 Study Group Descriptions and Determinants of Study Group Design

Patients assessed as having uncomplicated malaria during the Cohort Study will be randomized in open label fashion to treatment with either a 2 or 3 day course of DHA-piperaquine (DP) by directly observed therapy (DOT) at the MTF. Patients diagnosed with their first malaria malaria infection will receive a total of 9 tablets containing 40mg DHA and 320mg of piperaquine in divided doses at 0 and 24 hours (4.5 tablets once per day) for the 2 day course, or at 0, 24 and 48 hours (3 tablets once per day) for the 3 day course. Medication compliance for malaria treatment will be assured by directly observed therapy by study personnel during dosing.

The rationale and justification for this approach is that the 2 day course of DHA-piperaquine has currently become the *de facto* standard for treatment for RCAF personnel in Cambodia based on opinion of a visiting malaria expert from China. However, the published literature suggests that this regimen is not likely to be as effective as a 3 day regimen (see Background section). The efficacy of both regimens is uncertain in this population, and establishing a baseline treatment efficacy for DHA-piperaquine in this setting is important, particularly for potential future studies. Comparing the two groups most importantly will provide clinical evidence to support further policy decisions by RCAF as to which regimens are most effective in this setting.

5.6 Population to be Studied

The study population will include civilian and military personnel (including civilian beneficiaries) from civilian sites as well as multiple sites authorized by the Ministry of National Defense, Royal Cambodian Armed Forces, determined to have high incidence rates of malaria based on current estimates by AFRIMS, CNM and the RCAF health services. Active duty military personnel will be required to obtain permission from their Commanders prior to enrolling. Although exact numbers of RCAF personnel stationed in the high risk malarious areas is considered sensitive information, it is thought that there are at least several thousand potentially eligible volunteers. It is expected that the majority of enrollees will be young adult males, due to the nature of the intended study population. Pregnant volunteers will be excluded from participation in the study, due to risk of teratogenicity from artemisinin derivatives. Children and adolescents will not be enrolled in this study.

5.7 Study Sites and Selection of the Study Population

Volunteers enrolled into the Cohort Study will be healthy male and non-pregnant female patients (age 18 - 65 years), recruited from multiple RCAF sites in border areas of Cambodia. The exact nature and location of the sites are sensitive and will be determined by the investigators in consultation with RCAF Commanders and liaisons from

the Ministry of National Defense. A central urbanized area will be chosen to base the study team, and this will include a Medical Treatment Facility and appropriate laboratory facilities. Volunteers will be recruited from surrounding units and installations within 1-4 hours drive from the central area determined by the study team in cooperation with the RCAF Health Department as likely to have a high incidence of malaria based on available passively collected data. During the Cohort Study, Volunteers will be visited weekly, and referred to a central MTF by ambulance or study vehicle as appropriate with no extra charge if they are discovered to have malaria. Refer to the Background Section (3) for full details on health status and malaria burden in the intended study population.

5.8 Description of Test Article/Procedure and Placebo:

The test article during the Treatment Study will be the commercially available product Duo-Cotecxin, manufactured by Zhejiang Holley Nanhu Pharmaceutical Co., Ltd (see Appendix A for Package insert). This is the current first-line ACT recommended for in WHO containment Zone 1 in Cambodia (along the western border with Thailand). This is a combination tablet containing 40 mg of dihydroartemisinin and 320mg of piperaquine phosphate in each tablet. There are 8 tablets per package in the formulation provided to the Cambodian government. For consistency with current dosing guidelines following the advice of WHO, CNM recommends that clinics retain spare blister packages and provide each patient one additional tablet to take under DOT with two tablets from the package that the patient is given (see Appendix B, page 52). There will be no placebos used in this study.

Volunteers who develop malaria will receive either of two regimens depending on which group they are enrolled to at the time of initial diagnosis during the Cohort Study:

a. 2 day treatment group – 4.5 tablets on day 0 (at the time of diagnosis), and 4.5 tablets 24 hours later (+/- 1 hour).

b. 3 day treatment group – 3 tablets on day 0 (at the time of diagnosis), and at 24 and 48 hours later (+/- 1 hour).

Volunteers will be randomized to one of the two dosing regimens as described in section 5.3.

Volunteers with documented P. vivax exposure during the Cohort Study will be treated with primaquine phosphate obtained from a commercial source. Medication will be supplied in 15 and/or 30mg tablets to ensure the correct dose is administered.

All other antimalarial medications used during the study, to include Rescue Therapy (see Section 6.9.1) will be supplied by the National Center for Parasitology, Entomology and Malaria Control (CNM) of Cambodia, and administered by the Study Team (which includes CNM Physicians) according to current national guidelines for antimalarial treatment.

5.8.1 Packaging and Labeling of the Test Article/Procedure and Placebo

The test article will be used in its original commercial packaging, but administered by study personnel as described in section 5.7.

5.8.2 Storage of Test Article

The test article will be stored in a cool, dry place below 30° C in a light-proof container. The test article will remain under secure custody of the study team at all times.

5.9 Monitoring of Clinical Subject Safety

See Section 6.9 for full details regarding clinical assessments of Volunteer safety. Briefly, volunteers will be monitored during all phases of the study for adverse events. The most important component of monitoring will include the active malaria case detection and treatment which is the focus of the study. Treatment related adverse events are relatively rare at therapeutic doses with DHA-piperaquine, but include disturbances in cardiac potassium channel conductance which can prolong the QT interval on the EKG for piperaquine. This will be monitored by 12 lead EKG during treatment and up to 4 weeks post treatment for any QTc prolongation greater than 10 milliseconds over baseline persisting at MTF discharge.

To date there is not good evidence of clinically significant QT prolongation at therapeutic doses of piperaquine to be used in this study. Mytton et al (2007), published the results of two clinical trials evaluating QT prolongation following DHA-piperaquine therapy that found minimal QT prolongation indistinguishable from that attributable to malaria itself during and shortly after dosing over 48 hours. Further, the QT prolongations observed could not be distinguished from previously documented QT interval changes reported for other antimalarials without QT-prolonging properties. Inclusion or exclusion of subjects will be the responsibility of the investigators based on clinical judgment and expert consultation where necessary, and approval by both the local Medical Monitor and Sponsor's Medical Expert for inclusion of any subject with clinically significant abnormal EKG findings.

Neurological toxicity is rare at therapeutic dihydroartemisinin doses but has been reported, and will be monitored as part of routine clinical assessments to include a directed physical exam to further investigate neurological complaints as appropriate. Volunteers with G6PD deficiency who are treated with primaquine using the special G6PD-deficiency regimen (45 mg once per week for 8 weeks) will be evaluated with complete blood counts to detect hemolysis as outlined in Table 3.

Subjects in the study are enrolled at the beginning of the cohort study and will be treated as appropriate following national guidelines for malaria treatment (Section 5, Appendix B). Subjects with an initial presentation of *P. falciparum* at any time after enrollment will be randomized to 2 vs. 3 day DP treatment. However, subjects with signs of severe malaria at presentation may require treatment for severe malaria with parenteral therapy (see Section 3.1.3, Appendix B). While uncomplicated malaria often presents with mild hepatic and/or renal insufficiency, the criteria defining severe malaria with regard to renal and hepatic insufficiency are based on clinical evidence of organ dysfunction (oliguria and/or jaundice). Therefore, subjects with mild subclinical renal and/or hepatic insufficiency as

evidenced by clinical lab value abnormalities alone do not require parenteral therapy under the national treatment guidelines. To date there is not evidence in the literature that the pharmacokinetics of either drug is altered substantially in subclinical hepatic or renal insufficiency, and this study may in fact add evidence in this regard with careful measurement of both pharmacokinetics and renal and hepatic laboratory monitoring as outlined in the protocol.

The Medical Monitor (as required by AR 40-7) will be a licensed physician based at a Military Referral Hospital in Cambodia. His duties include monitoring the study volunteers from a medical perspective, reviewing and reporting all serious and unexpected adverse events, ensuring medical care for any such events should they occur, and reporting serious events to higher headquarters. The Sponsor's Medical Expert will also review all reports from the Medical Monitor including unexpected and Serious Adverse Events, and state concurrence or non-concurrence with the report and request additional information as necessary.

6. METHODS

6.1 Recruitment of Study Volunteers

The study team will identify potential volunteers in cooperation and with permission from local RCAF Commanders based on logistical considerations, mission constraints, and likely burden of malaria. The study population of civilian and military personnel (including their dependents) will be recruited from areas identified through existing data as having high risk of malaria transmission. There will be no written advertising or recruiting materials. Any questions asked of volunteers during the recruitment process (prior to informed consent) will respect the privacy of their protected health information.

The study team includes key members of the RCAF military medical department. Permission from the Commander of military facilities or units in which recruitment will occur or the study will be conducted will be requested. Special consideration will be given to the recruitment process for military personnel. The Chain of Command will not be involved in the recruitment of military personnel and will not encourage or order soldiers to participate in a research study. Per DOD Directive 3216.2, an ombudsman will be employed when conducting group briefings with active duty personnel to ensure that volunteers understand that participation is voluntary. The ombudsman will be present in other situations as appropriate, and will be available to Volunteers to answer questions.

6.2 Informed Consent Process

Title 10 United States Code 980 requires that "Funds appropriated to the Department of Defense may not be used for research involving a human being as an experimental volunteer unless- (1) the informed consent of the volunteer is obtained in advance; or (2) in the case of research intended to be beneficial to the volunteer, the informed consent may be obtained from a legal representative of the volunteer."

NOTE: Furthermore and consistent with the Common Federal Policy for the Protection of Human Volunteers, if an individual cannot give his or her own consent to participate in a research study, consent of the individual's legally authorized representative must be obtained prior to the individual's participation in the research. Moreover, an individual not legally competent to consent (e.g., incapacitated individuals, incompetents, minors) may not be enrolled in DOD sponsored research unless the research is intended to benefit each volunteer enrolled in the study. For example, a volunteer may benefit directly from medical treatment or surveillance beyond the standard of care. Volunteers will benefit in this study by enhanced surveillance for active case detection and treatment afforded by the research team.

Informational briefings as part of the informed consent process may be done individually or in groups. In either case, an ombudsman will be present during the consent process for all military volunteers, and available to answer questions or represent the volunteers for any concerns that arise in the course of study participation. Volunteers will be given the opportunity to discuss questions of a personal nature privately with the investigators and/or the ombudsman if desired following the briefing. Potential volunteers will be instructed that they have the option to refuse to participate in the study, and to withdraw

at any time. No study procedures will occur prior to the volunteer giving informed consent as indicated by a signed, witnessed informed consent document.

Informed consent is a process that is initiated prior to the individual's agreeing to participate in the study and continuing throughout the individual's study participation. Extensive discussion of risks and possible benefits of participation in this study will be provided to the volunteers and their families. Consent forms in the local language (Khmer) describing in detail the study procedures and risks will be given to the volunteer and written documentation of informed consent will be obtained prior to enrollment in the study. Consent forms will be IRB approved and the volunteer will be asked to read and review the document. If the volunteer cannot read the content of the consent form, it will be read and explained to him by the designated unit Ombudsman, assisted by study personnel. Upon reviewing the document, the study personnel will explain the research study to the volunteer and answer any questions that may arise. Volunteers will then be asked to complete a 10 question test of understanding on which they will need to score at least 80% (3 attempts maximum). After successful completion and the volunteer acknowledges that they have had adequate time to review of the ICF, the volunteer will be asked individually to sign and date the informed consent document prior to being enrolled in the study.

One witness will sign and date the consent form in the presence of the participant attesting that the requirements for informed consent have been satisfied and that consent is voluntary and freely given by the volunteer without any element of force, fraud, deceit, duress, coercion, or undue influence. Volunteers will have the opportunity to discuss the study with the assigned unit ombudsmen if military personnel or think about it prior to agreeing to participate. Participation in the study will be voluntary and volunteers will be informed that they may withdraw consent at any time throughout the course of the study. Assigned ombudsman may sign and date the ICF as the witness. Following ICH guidelines a signed copy of the informed consent document will be given to the volunteers for their records. The rights and welfare of the volunteers will be protected by emphasizing to them that the quality of their medical care will not be adversely affected if they decline to participate in this study.

6.3 Screening Procedures

Following documentation of informed consent, study Volunteers will have an initial targeted medical history and physical examination performed by a study physician. Female Volunteers will have a urine pregnancy test. Prior clinical history of malaria will be assessed by a directed, volunteer completed-questionnaire with a follow-up interview by a provider. Volunteers will then have a clinical assessment including a medical history and a directed physical examination (with assessment for hepato-splenomegaly, and neurologic abnormalities). Volunteers will have a 12-lead EKG. Volunteers will have blood drawn for PCR-corrected malaria blood smears, an MSP-1 titer, CBC, G6PD activity (in case radical cure of *P. vivax* is required), blood renal function and liver function testing at baseline. Volunteers not meeting all study inclusion and/or exclusion criteria will not be enrolled into the study. A log will be kept of all who were evaluated for participation to document who was and was not enrolled to include ID number for each screened person, the individuals' name, age, gender, screening date, and eligibility status.

6.4 Determination of Eligibility

6.4.1 Inclusion Criteria

Volunteers meeting all of the following criteria will be considered eligible for enrollment in the study:

1. Otherwise healthy volunteer, 18-65 years of age, eligible for care at an RCAF facility, or otherwise eligible Cambodian civilians at risk for contracting malaria
2. Able to give informed consent
3. Likely to reside in endemic area for the duration of the study
4. Available for follow-up for anticipated study duration, and agrees to participate for the duration of the study
5. Authorized by local commander to participate in the study if on active duty

6.4.2 Exclusion Criteria

Volunteers meeting any of the following criteria will be excluded from the study:

1. History of allergic reaction or contraindication to DHA or piperaquine
2. Significant acute co-morbidity requiring urgent medical intervention
3. Pregnant or lactating female, or a female of childbearing age who does not agree to use a highly effective method of birth control during the study
4. Clinically significant abnormal EKG, including a QTc interval > 500 ms
5. Judged by the investigator to be otherwise unsuitable for study participation

WHO guidelines state that in uncomplicated malaria, artemisinin combination treatment should only be used starting in the second trimester, with use in the first trimester only if no other effective treatment is available. Therefore, avoidance of conception during the potential period of treatment in the study is warranted. The guidelines on contraceptive use are based on FDA guidance M3 "Nonclinical Safety Studies for the Conduct of Human Clinical Trials for Pharmaceuticals" which describes safety considerations for the inclusion of women of childbearing age in studies of healthy volunteers. This guidance is nearly identical to that of ICH M3. The guidance requires that a highly effective method of birth control be used by women of childbearing age in healthy volunteer studies. According to the published guidance, "A highly effective method of birth control is defined as one that results in a low failure rate (i.e., less than 1 percent per year) when used consistently and correctly, such as implants, injectables, combined oral contraceptives, some intrauterine contraceptive devices (IUDs), sexual abstinence, or a vasectomized partner. For subjects using a hormonal contraceptive method, information regarding the product under evaluation and its potential effect on the contraceptive should be addressed." The guidelines are noteworthy for omitting mention of women who have undergone surgical sterilization (these individuals would be included in the study) in addition to vasectomized partners. Clinically significant drug-drug interaction with hormonal contraceptives appears unlikely. Piperaquine undergoes very little metabolic transformation in humans and as a result is unlikely to affect the level of hormonal contraceptives (Liu et al, 2007). Artesunate and dihydroartemisinin are not extensively

metabolized in liver, and there is no significant effect on the cytochrome P450 enzyme system (in vitro data) (Bangchang et al, 1992; Barradell & Fitton, 1995a).

At study entry, females will be counseled to agree to avoid becoming pregnant during their entire participation in the study, and for at least one month after the last dose of any study medication that may be administered. Female volunteers who suspect that they may be pregnant will be instructed to inform study personnel as soon as possible.

All females between the age of 18 and 50 will be screened with a urine pregnancy test at baseline. Pregnant or lactating females, and females of childbearing age who do not agree to use a highly effective method of birth control will be excluded from participation in the study. Exclusion from study participation will be determined by the Principal Investigator, when a volunteer is found to be pregnant at baseline, or prior to treatment for malaria if contracted during the study. For females who are diagnosed with malaria, this will include an additional urine pregnancy test prior to malaria treatment, and a follow-up pregnancy test at 42 day post-treatment follow-up. Females found to be pregnant prior to treatment will be treated according to current Cambodian national malaria treatment guidelines for the treatment of malaria in pregnancy published by the Ministry of Health.

In the highly unlikely event that a female volunteer becomes pregnant during the 3 days of malaria treatment following an initial negative urine pregnancy test at the initiation of treatment, she will be discontinued from study medication and will be treated according to current Cambodian national malaria treatment guidelines for the treatment of malaria in pregnancy published by the Ministry of Health. If a woman is found to be pregnant less than 1 month after completing a course of study medication, she will be followed for safety at 3, 6 and 9 months at which time the health and birth weight of the child will be assessed. All such pregnancies temporally associated with study drug administration will be reported to the IRBs urgently.

6.5 Randomization and Volunteer Assignment

Volunteers developing malaria infection during the active Cohort Study follow-up period will be treated as part of a two arm, randomized, open label treatment study comparing the efficacy (42 day PCR-corrected malaria recurrence), safety, tolerability and pharmacokinetics of a two versus three day course of Dihydroartemisinin-Piperaquine (DP). The primary end-point will be comparative 42 day malaria cure rates. Volunteers developing malaria will randomized into the two treatment arms using time-blocked randomization with a block size of two.

6.6 Blinding

Although open label (unblinded), microscopists are blinded to each others' readings and to regimen. There is otherwise no blinding during the study.

6.7 Administration of Test Article and Placebo (or other Research Intervention)

The test article will be obtained from a commercial supplier. The schedule for administration is detailed in Table 2, (Section 6.9) below for test article, and under Section 6.14 – Rescue Therapy for all medications used as rescue therapy. Only those members of the Study Team designated by the Principal Investigators will be authorized to prescribe and/or administer the test article, based on their qualifications as appropriate. Any unused medications remaining at the end of the study will be provided to the RCAF for clinical use.

Volunteers with documented P. vivax exposure during the Cohort Study will be treated with primaquine phosphate obtained from a commercial source. Medication will be supplied in 15 and/or 30mg tablets to ensure the correct dose is administered.

All other antimalarial medications used during the study, to include Rescue Therapy (see Section 6.14) will be supplied by the National Center for Parasitology, Entomology and Malaria Control (CNM) of Cambodia, and administered by the Study Team (which includes CNM Physicians) according to current national guidelines for antimalarial treatment.

6.8 Concomitant Medications

Use of concomitant medications will be evaluated by the investigator at each clinical encounter with the volunteer. Use of antimalarials or drugs with known antimalarial activity other than those prescribed by an investigator during the study will not be permitted. While drugs that interact with or otherwise have a known unfavorable impact on the outcomes of interest in the study will be avoided by investigators during the malaria treatment phase, there are no other explicitly restricted concomitant medications during this study.

6.9 Clinical Assessments

Following documentation of informed consent, study Volunteers will have an initial targeted medical history and physical examination including a 12 lead electrocardiogram performed by a study physician. Female volunteers will have a urine pregnancy test. Prior clinical history of malaria will be assessed by a directed questionnaire with a follow-up interview by a provider. Volunteers will then have a clinical assessment including a physical examination with vital signs. Volunteers will have blood drawn for PCR-corrected malaria blood smears, an MSP-1 antibody titer, CBC, G6PD activity (in case radical cure of *P. vivax* is required), renal function and liver function testing at baseline (see Table 1).

Volunteers will be followed weekly by study personnel with a brief clinical assessment (to include determination of temperature) for the duration of the specified Cohort Study follow-up period. The assessment will include an open-ended, non-leading assessment of adverse events by an investigator or designee. Volunteers that remain symptom free will have malaria smears performed every month. Volunteers with a clinical suspicion of malaria at routine follow-up, or at any time during the study will have a blood film examination for malaria. If the smear is negative, alternative diagnoses for febrile

illnesses will be described as accurately as possible based on available diagnostic capabilities.

Volunteers developing malaria infection during the active observation follow-up period (including enrollment) will be treated as part of a two arm, randomized, open label Treatment Study comparing the efficacy (42 day PCR-corrected malaria recurrence), safety, tolerability and pharmacokinetics of a two versus three day course of Dihydroartemisinin-Piperaquine (DP). All Volunteers with signs or symptoms of malaria discovered at any time during the Cohort Study will be evaluated with malaria microscopy. This may occur during weekly clinical follow-up, monthly routine blood smear (if asymptomatic), or at any time that the volunteer reports symptoms to the study team directly or through a unit liaison. Active detection, rapid diagnosis of suspected malaria cases and prompt treatment will be the highest priority of the Cohort Study.

Volunteers with positive malaria smears will be referred to the Medical Treatment Facility (MTF) for care by the study team. Volunteers will be first assessed for signs or symptoms of Severe Malaria as outlined in Appendix B. Table 2 lists the time-event schedule for volunteers diagnosed for the first time with uncomplicated malaria. Female volunteers will have a urine pregnancy test, and if positive, they will not receive Test Article, and will be treated under national guidelines for the treatment of malaria in pregnant patients (see Appendix B). Volunteers will have blood collected for malaria PCR, MSP-1 level and a baseline piperaquine drug level (necessary to standardize the bio-analytical chemistry analysis). Blood will also be collected for parasite culture and *in vitro* drug resistance characterization, and analysis of molecular markers of infection. Following the baseline clinical assessment, patients who present with danger signs indicating severe or complicated malaria infection will be treated under national guidelines for treatment of severe malaria, which currently include the use of intramuscular artemether or intravenous artesunate. Patients assessed as having uncomplicated malaria will be randomized in open label fashion to treatment with either a 2 or 3 day course of DHA-piperaquine (DP) by directly observed therapy (DOT) at the MTF. Patients will receive a total of 9 tablets containing 40mg DHA and 320mg of piperaquine in divided doses at 0 and 24 hours (4.5 tablets each) for the 2 day course , or at 0, 24 and 48 hours (3 tablets each dose) for the 3 day course. Medication compliance for malaria treatment will be assured by directly observed therapy by study personnel during dosing.

During treatment, patients will have vital signs including temperature, blood pressure, pulse and respirations evaluated, and blood drawn for malaria smears at 4 and 8 hours after the first dose of medication, and then every 8 hours until 2 consecutive negative smears are obtained. In addition, blood will be drawn for pharmacokinetic piperaquine levels at 4, 24, 48 and 72 hours post dose, then weekly for 42 days. Blood will be collected again for CBC, parasite *in vitro* drug resistance characterization, and analysis of molecular markers of resistance at 24, 48 and 72 hours after the initiation of treatment. Volunteers will have a 12-lead EKG performed at the time of diagnosis, and then daily until treatment of uncomplicated malaria is complete. If the QTc interval is prolonged over baseline measurement by more than 10 milliseconds at discharge, then 12 lead EKG will be repeated weekly until normalized, or until week 4 post treatment. Patients will be discharged from the MTF at clinical and parasitological resolution of malaria infection (at least 2 consecutive negative smears), and followed weekly until day 42, resuming their assigned weekly follow-up schedule within the Cohort Study. The flow of volunteers and procedures during the Cohort Study and Treatment Study are outlined in Figure 1.

Table 1. Time-event diagram of routine follow-up for the malaria epidemiology Cohort Study.

Event	Screening / Enrollment	Week 01	Week 02	Week 03	Week 04	Weekly from Week 05 to Cohort Study Discharge	Cohort Study Discharge[6]	Suspected malaria?
a. Informed consent	x							
b. Medical history	x							
c. Physical exam with EKG	x							
d. Brief clinical evaluation[1]		X	x	X	x	X	x	x
e. Monthly malaria blood smear[2]	~0.25mL				fs	fs[2]	fs	fs[2]
f. Malaria antibody titer (e.g. MSP-1)[5]	2 ml						2 mL	2 mL
g. G6PD screen[5]	0.5 mL							
h. Liver Function, Renal Function Test	2 mL						2 mL	2 mL
i. CBC	2 mL						2 mL	2 mL
j. Parasite culture *in vitro* resistance								x
k. Malaria treatment[3]	(x[3])							x[3]
l. Radical cure treatment (PQ)[4]							x[4]	
Cumulative phlebotomy	~7 mL				~7.5 mL	Up to 10.5 mL	~16 mL	~22 mL

Notes:
1. Brief clinical evaluation includes a weekly interval medical history with adverse event inquiry, measurement of temperature and a directed physical exam as clinically indicated.
2. Malaria smears will be collected by Fingerstick (approximately 0.25 mL of blood) monthly in the absence of symptoms, or at any time the Volunteer reports symptoms suggestive of malaria.
3. Patients will be referred to a medical treatment facility (MTF) for treatment. See Figure 1. Patients diagnosed with their first malaria malaria infection will receive a total of 9 tablets containing 40mg DHA and 320mg of piperaquine in divided doses at 0 and 24 hours (4.5 tablets once per day) for the 2 day course, or at 0, 24 and 48 hours (3 tablets once per day) for the 3 day course. Patients with severe malaria, or with a malaria recurrence will be treated according to National Guidelines. Refer to Table 2. While the study will attempt to enroll healthy subjects, patients discovered incidentally to have malaria at the time of screening (eg asymptomatic parasitemia) will still be enrolled, and treated immediately as outlined in Table 2 and Figure 2
4. Volunteers who develop primary *P. vivax* during the course of the study (including at enrollment) or have an apparent relapse from latent liver-stage disease will be treated with DHA-piperaquine to clear blood stage infection during the cohort follow-up period of the study. Volunteers who develop *P. vivax* blood stage malaria recurrence during the Cohort Study will be treated with chloroquine 25mg/kg per day for 3 days up to a maximum of 1500mg total according to the current National Treatment Guidelines (see Appendix B). All Volunteers who have had known exposure to *P. vivax* infection will be treated at the **end of the cohort follow-up period (discharge visit)** with primaquine 30mg per day for 14 days to eradicate liver stage infection once all blood stage treatment has been completed. Volunteers with known G6PD-deficiency will be treated with the current US CDC-recommended regimen for G6PD-deficient patients with 45mg of primaquine weekly for 8 weeks with ongoing follow-up by the study team. Volunteers who receive treatment with primaquine will be followed passively for 6 months to estimate rates of malaria recurrence. See Table 3.
5. Blood for malaria antibody titers to antigens such as MSP-1 will be collected in a serum separator tube (portion spun and separated as plasma) and G6PD activity testing collected in EDTA.
6. Cohort Study discharge is expected to occur after roughly 12-20 weeks, but will be dependent on the malaria attack rate, and the number of subjects randomized to malaria treatment (minimum of 80 subject enrolled) – see Study Duration section for full details.

Table 2. Table of times and events for Volunteers diagnosed with malaria and randomized to the Treatment Study.

Event	Malaria Diagnosis	4 hr	q8 hr[9]	24 hr	48 hr	72 hr	MTF Dis[10]	Week 01[12]	Wk 02	Wk 03	Wk 04	Wk 05	Wk 06	Malaria Recurrence
a. Medical Treatment Facility Referral	x													x
b. Interval medical history	x													x
c. Physical exam	x													x
d. Brief clinical evaluation[1]		X	x	x	x	x	X	x	x	x	x	x	x	x
e. Malaria smear (with vital signs)[2]	~0.25 mL	Fs	fs	fs	fs	fs	Fs	fs	fs	fs	fs	fs	fs	fs
f. Piperaquine drug level	2 mL	2mL		2mL	2mL	2mL		2mL	2mL	2mL	2mL	2mL	2mL	2mL
g. Malaria antibody titer (e.g. MSP-1)[3]	2 ml												2mL	2mL
h. EKG[4]	x			x	x	x	X	(x)[4]	(x)[4]	(x)[4]	(x)[4]			
i. Renal function and Liver Function[5]	2 mL					2mL								2mL
j. CBC[6]	2 mL			2mL	2mL	2mL								2mL
k. Parasite culture *in vitro* resistance[7]	8 mL			8mL	8mL	8mL								8mL
l. Molecular resistance markers[7]	6 mL			6mL	6mL	6mL								6mL
m. Randomization and Malaria treatment[8]	x			x	(x)									x[11]
Cumulative Total Phlebotomy (mL)	~22.5 mL	~24.5	~25	~44	~62.5	~84	~84.5	~87	~89.5	~92	~94.5	~97	~101.5	~123

1. Brief clinical evaluation includes an interval medical history, vital signs and a directed physical exam as clinically indicated.
2. Malaria smears (PCR-corrected) and vital signs to include temperature, blood pressure, pulse and respiratory rate will be collected at 0, 4 and 8 hours, and then every 8 hours thereafter until the volunteer has had 2 negative blood smears at least 8 hours apart.
3. Blood for malaria antibody titers to antigens such as MSP-1 will be collected at baseline and at any malaria recurrence.
4. Volunteers will have a 12 lead EKG prior to antimalarial dosing, and daily as part of directly observed therapy until discharge. Volunteers that have a QTc prolongation greater than 10 milliseconds over baseline at discharge will have the EKG repeated weekly for 4 weeks or until normalized to baseline level.
5. 2mL of blood for liver function tests, and renal function will be collected in serum separator or plain tubes.
6. 2mL of blood will be collected in EDTA tubes for CBC.
7. Volunteers will have 8 mL blood in sodium heparin tube drawn for malaria parasite culture and in vitro drug resistance testing before medication dosing, and at 24 hours after the first dose. Volunteers will have 6 mL of blood drawn in EDTA tubes (including a filter paper blood spot) for analysis of molecular markers of malaria parasite drug resistance before medication dosing, and at 24 hours after the first dose.
8. Patients diagnosed with their first malaria infection will receive a total of 9 tablets containing 40mg DHA and 320mg of piperaquine in divided doses at 0 and 24 hours (4.5 tablets once per day) for the 2 day course, or at 0, 24 and 48 hours (3 tablets once per day) for the 3 day course.
9. Malaria microscopy smears will be performed at baseline and every 8 hours until the patient has had two negative smears at least 8 hours apart.
10. Volunteers will be discharged from the MTF once they are afebrile and have had 2 consecutive negative malaria smears at least 8 hours apart.
11. For Volunteers that have recurrent malaria following treatment with DHA-piperaquine, current national treatment guidelines for Cambodia will be adhered to as clinically appropriate at all times. Current national malaria treatment guidelines for adults with uncomplicated *P. falciparum* in Cambodia include 200mg oral artesunate for 3 days, and 500mg mefloquine on days 1 and 2, with 250mg on day 3. Rescue therapy for those experiencing recurrence following the above regimen include 7 days of daily combination oral quinine (30mg/kg/d) + tetracycline (25mg/kg/d) therapy. Current standard of care rescue therapy for recurrent *P. vivax* blood stage infection in Cambodia is repeat treatment with chloroquine 25mg/kg per day for 3 days up to a maximum of 1500mg total (see Appendix B).
12. Volunteer follow-up will resume Cohort Study with previously established weekly follow-up schedule.

Figure 1. Schematic of study flow for volunteers enrolled in the Cohort Study and referred to the Treatment Study (at first occurrence of uncomplicated malaria).

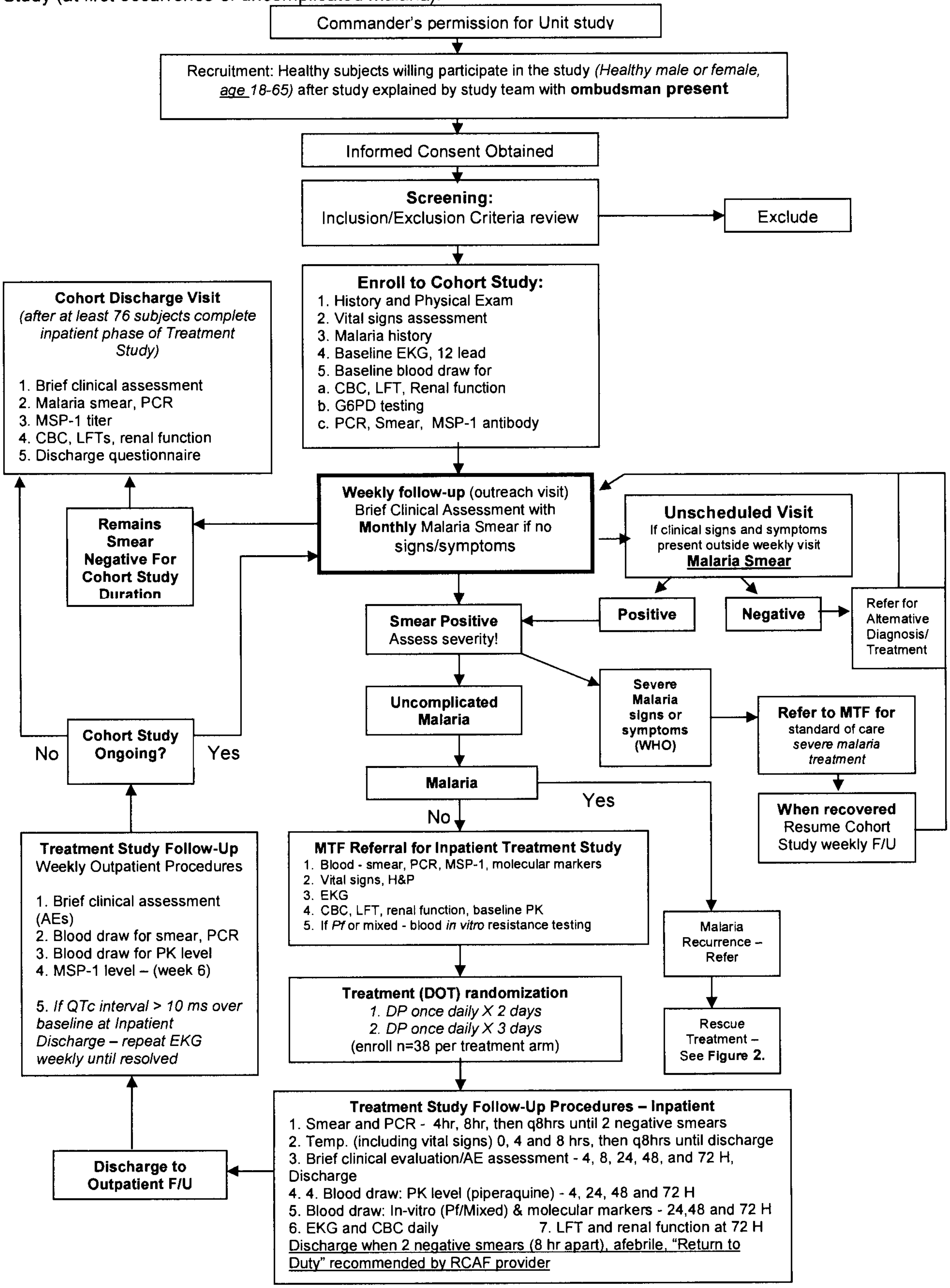

All Volunteers with suspected recurrent malaria symptoms during the follow-up period of the Cohort Study will be re-evaluated by microscopy, and if positive for malaria will be treated under directly observed therapy based on current national malaria treatment guidelines for Cambodia (see Appendix B). If negative for malaria, the patient will be referred for evaluation and treatment of alternative diagnoses to the appropriate RCAF healthcare service providers. Volunteers found to have recurrent *P. falciparum* malaria after initial re-treatment with first line therapy (which is currently a 3 day regimen of artesunate and mefloquine) will be treated with rescue therapy following current national guidelines. At the time of writing, this includes treatment with quinine and doxycycline for 7 days. Blood smears following rescue therapy will be collected daily until resolution by 2 negative smears, at least 1 week apart. An active effort will be made to locate Volunteers lost to follow up for any reason during the Cohort and/or Treatment Study for repeat of PCR-corrected malaria smears, and MSP-1 levels. At the conclusion of the Cohort Study (as determined by the Investigators based on the number of malaria cases treated during the study), Volunteers will have blood drawn for PCR-corrected malaria blood smears, an MSP-1 titer, CBC, renal, and liver function at discharge. The discharge questionnaire will also assess willingness to participate in future clinical trials. Figure 2. illustrates the treatment of volunteers with recurrent malaria, and use of primaquine for volunteers with documented *P. vivax* blood stage infection during the study.

Volunteers already treated for malaria initially under the protocol who subsequently develop primary blood stage P. vivax or who have an apparent relapse from latent liver-stage disease will be treated according to current Cambodian national malaria treatment guidelines (currently a 3 day course of chloroquine) to clear blood stage infection during the cohort follow-up period of the study (Cohort Study). At the completion of the Cohort Study, all volunteers who have had known exposure to P. vivax infection (documented by malaria microscopy) during the Cohort Study will be treated with primaquine 30mg per day for 14 days to eradicate liver stage infection (see Table 3). Volunteers with documented G6PD-deficiency at baseline will be treated with the US CDC's currently recommended regimen for G6PD-deficient patients (45mg of primaquine weekly for 8 weeks). G6PD deficient Volunteers will receive all doses by directly observed therapy by the study team, and have a CBC drawn before the first dose of primaquine is given. CBC will be repeated on day 3 after the first dose, and then again before the second dose is administered (one week after the first dose). Volunteers will clinically significant declines in hemoglobin values will be discontinued from 8-aminoquinoline therapy, and instructed to seek medical attention in the future if they develop signs or symptoms of malaria (see Figure 2). Volunteers treated with post-exposure 8-aminoquinoline therapy to eradicate P. vivax liver stages will have ongoing follow-up by the study team in coordination with the Ministry of National Defense Health Department monthly with questionnaires (see Appendix E – Source Documents / Case Report Forms) for 6 months to estimate rates of malaria recurrence. The study team will seek to obtain medical records from the relevant clinical encounters from all Volunteers who report a malaria recurrence to the study team during the 6 month follow-up period. Cumulative incidence of primary P. vivax infection and recurrence will be estimated. Clinical history and molecular parasite analysis will be used to determine whether recurrence represents a relapse, or new infection where possible.

Therefore, clinical follow-up for P.v.-exposed G6PD-normal patients by the study team will be extended for 14 days after the Cohort Study is completed, and extended for 8 weeks for G6PD-deficient patients to complete DOT of primaquine. Once clinical follow-up is completed, all Volunteers treated with primaquine for *P. vivax* radical cure will be followed

by the study team in coordination with the Ministry of National Defense Health Department monthly for 6 months to estimate rates of malaria recurrence with instructions to contact the study team by telephone if malaria-like symptoms are encountered. The study team will attempt to contact volunteers monthly in the absence of contact by the volunteer. Volunteers who report symptoms of malaria will be referred for consultation at an RCAF treatment facility, and will have their medical records reviewed by the study team as soon as practicable.

Figure 2. Rescue treatment and follow-up procedures for volunteers found to have recurrent malaria during Cohort Study follow-up, including P. vivax antirelapse therapy.

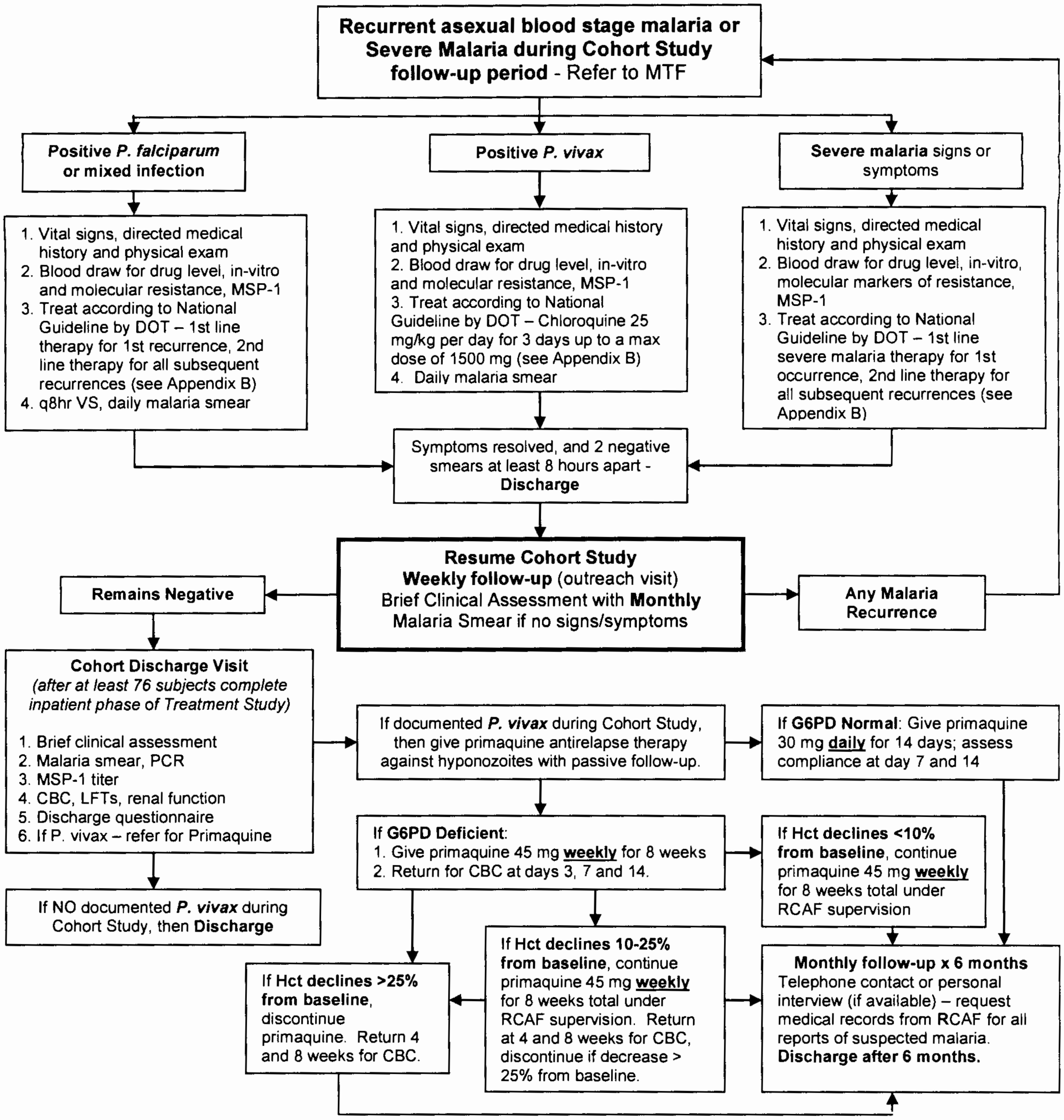

Table 3. Additional table of times and events for Volunteers treated for radical cure of P. vivax at study discharge.

Event	Cohort Discharge Visit	Day3 post study	Day 7	Day 14	Month 1	Month 2	Month 3	Month 4	Month 5	Month 6
a. Review G6PD status	x									
b. Brief clinical evaluation[1]	x	(x)[5]	(x)[5]	X	(x)[5]	(x)[5]				
c. Telephone follow-up[2]					x[2]	x[2]	x	x	X	x
e. Radical cure treatment if G6PD normal (PQ 30mg per day for 14 days)[3]	x	x	x	X						
f. Radical cure treatment if G6PD-deficient (PQ 45mg once weekly for 8 weeks)[4]	x		x	X	xx	xxx				
g. CBC if G6PD-deficient[5]	2 mL	2 mL	2 mL	2 mL	(2 mL)[5]	(2 mL)[5]				

1. Only Volunteers with documented P. vivax exposure defined as evidence of P. vivax blood stage infection occurring during the epidemiology cohort study will be referred for radical cure treatment for eradication of exoerythrocytic stage malaria forms using primaquine. Brief clinical evaluation of includes an interval medical history and a directed physical exam as clinically indicated. This will be performed on day of discharge from the Cohort Study and at day 14 on completion of radical cure therapy for G6PD-normal Volunteers. Medication compliance with primaquine will be assessed on Day 7 and 14 for all G6PD-normal Volunteers. Volunteers who are G6PD deficient will have additional assessments (see item 5 below).
2. Volunteers will be followed up monthly for 6 months after completing primaquine treatment. Volunteers will be asked if they have had any symptoms of and/or been treated for malaria. Volunteers responding in the affirmative will be asked to provide their medical records from the dates of treatment to the study team for review. Record transmittal will be coordinated through the Health Department, Ministry of National Defense, Royal Cambodian Armed Forces. Volunteers will be treated by RCAF providers at RCAF facilities - the study team will not be involved directly in the care of patients during the telephone follow-up phase.
3. Volunteers who develop primary *P. vivax* during the course of the study (including at enrollment) or have an apparent relapse from latent liver-stage disease will be treated with DHA-piperaquine to clear blood stage infection during the Cohort Study follow-up period and until the day of discharge from the Cohort Study. Volunteers who develop *P. vivax* blood stage malaria recurrence will be treated with chloroquine 25mg/kg per day for 3 days up to a maximum of 1500mg total (see Appendix B) during the period of the cohort study. Once this has been completed, all Volunteers who have had known exposure to *P. vivax* infection will be treated at the **end of the cohort follow-up period (discharge visit)** with primaquine 30mg per day for 14 days to eradicate liver stage infection.
4. Volunteers with known G6PD-deficiency will be treated with the currently recommended regimen for G6PD-deficient patients by the US Centers for Disease Control and Prevention with 45mg of primaquine weekly for 8 weeks with ongoing follow-up by the study team. Volunteers will return for clinical follow-up visits on days 3, 7, and 14, and at 1 and 2 months post dose for directly observed therapy. On days 28 and 56, Volunteers will receive additional doses of medication to complete an 8 week course under supervision by the unit liaison. Volunteers will be followed for an additional 4 months to estimate rates of malaria recurrence.
5. Up to 2 mL of blood will be collected in EDTA tubes for Complete Blood Count for G6PD-deficient Volunteers only (45mg once per week for 8 week regimen). This will be performed at day 0, 3, 7 and 14 following the first dose of primaquine. If hematocrit decreases by less than 10% from baseline on all 3 measurements, no further CBCs will be performed. If hematocrit drops between 10 and 25%, CBC will be repeated on days 28 and 56. If hematocrit drops below the baseline value by more than 25% at any time, primaquine will be discontinued.

6.10 Specimen (or Data) Collection and Testing

6.10.1 Specimens to be Collected

The following specimens will be collected as outlined in the schedule in Tables 1, 2 and 3, and Figures 1 and 2.

- Fingerstick capillary or venous blood will be collected for PCR-corrected microscopic blood smears to determine parasite species, and to quantify parasitemia (approximately 250μL of blood per sample). PCR correction will be performed on parasite DNA extracted either from a blood spot filter paper, or approximately 200μL of whole blood from venipuncture.

- **Hematology** to include hemoglobin, hematocrit, WBC count and differential, platelet count, and cell indices (see section 6.10.2 F). Approximately 2 ml per blood draw will be collected in an EDTA (anticoagulant) tube.

- **Renal Function** (creatinine, urea) and **Liver Function Tests** (aspartate aminotransferase, alanine aminotransferase, alkaline phosphatase, total bilirubin). Approximately 2 ml per blood draw will be collected in a serum separator blood tube.

- **Glucose-6-phosphatase deficiency** (by fluorescence testing) with single nucleotide polymorphism analysis if positive at enrollment. Approximately 0.5 mL per blood draw will be collected in an EDTA (anticoagulant) tube.

- Malaria antibody titers to antigens such as MSP-1(approximately 2 ml) in a serum separator tube.

- Patients will have 8 mL blood in sodium heparin tube drawn for malaria parasite culture and **in vitro drug resistance** testing before medication dosing, and at 24, 48 and 72 hours after the first dose. Patients will have 6 mL of blood drawn in EDTA tubes for analysis of **molecular markers** of malaria parasite drug resistance before medication dosing, and at 24, 48 and 72 hours after the first dose. Both tests will be repeated at the time of diagnosis for any malaria recurrence. From this 6ml of blood, 300μl of blood will be spotted on filter paper and dried for stabilization of DNA and RNA. At each weekly follow up thereafter, blood obtained by finger stick at the same time a peripheral blood smear is done will also be spotted on filter paper and dried for stabilization of DNA and RNA.

- Urine pregnancy test: Urine beta-HCG test. All female subjects age 18-50 will undergo a pregnancy test at baseline, prior to malaria treatment, and at 42 day post-treatment follow-up. Pregnant women will not be eligible for entry into the study.

6.10.2 Specimen Preparation, Processing, Handling, and Storage

A. Malaria Microscopy - Stained thick and thin blood smears will be examined by two microscopists who are blinded to each other's results and to the treatment status of the study volunteer. Two blood smears will be made for every enrolled patient. Slide 1 will be

stained immediately and examination of giemsa stained thick and thin smears. This slide will then be stored in a different box from slide 2, which will only be read there is a problem with the first slide.

Parasite densities will be calculated based on a count of parasites per 200 WBCs (thick film) or per 5000 RBCs (thin film). A total of 200 oil immersion fields will be examined on the thick film before a blood smear is considered negative. The final count will be determined by taking the geometric mean of the two counts. In case of a difference in results (positive/negative; species diagnosis) between the two microscopists, the blood smear will be re-examined by a third microscopist blinded to the results of the first two readers and the treatment regimen, and the third reading will be accepted as the final result.

B. PCR Correction - Malaria microscopy results will be confirmed using real-time PCR correction to genotype parasite DNA collected and isolated from 250 up to 500 μL of venous or capillary blood (collected on filter paper and in EDTA tube). Parasite densities will be quantified using PCR results and compared with microscopy.

C. G6PD Deficiency Testing - Venous blood will be tested for qualitative G6PD activity using the fluorescent spot test method, as is recommended by the International Committee for Standardization in Hematology using commercially available kits. This method detects fluorescence of NADPH under long-wave (365 nm) UV light. Reduction of NADP to NADPH occurs in the presence of G6PD. The rate and extent of NADPH formation is proportional to G6PD activity. Normal samples fluoresce brightly, whereas deficient samples show little or no fluorescence. DNA will be extracted from ~ 0.5 ml of blood collected in EDTA, and the G6PD gene will be genotyped according to established methods (Fujii et al., 1984). **No human genetic studies will be performed other than to assess G6PD genotypes.** Genotype data may be compared against existing databases, such as sequence data from other samples located in publicly accessible database(s) for example GENBANK

D. In vitro drug sensitivity. For P.f. monoinfection and mixed P.f. and other parasite species infections, ~ 8 mL of heparinized blood will undergo in vitro drug sensitivity testing using established methods (Noedl et al., 2004 and Noedl, 2005). Other methods for evaluating in vitro drug sensitivity may also be employed. A portion of the specimen will be cryopreserved according to established procedures. Culture adaptation will be performed either at AFRIMS or other collaborating laboratories according to established methods (Trager and Jensen, 1997).

Heparinized blood (~ 4 ml) will be cryopreserved with an appropriate cryopreservative solution according to a protocol developed at AFRIMS adapted from MR4 and stored in liquid nitrogen for culture adaptation. Blood samples for in vitro drug sensitivity testing will be stored under assay-appropriate conditions. In vitro drug sensitivity assays will be performed directly at the field sites. The remaining blood will be stored in liquid nitrogen for future drug sensitivity analysis.

For volunteers with P. vivax mono-infections, heparinized blood will be collected and used fresh onsite or stored under conditions yet to be determined for development of in vitro

culture and/or drug sensitivity methods, or transported to a reference lab covered by this protocol for further method development.

E. Parasite genomics. Anticoagulated blood will be cryopreserved and stored in liquid nitrogen for culture adaptation. Blood samples for in vitro drug sensitivity testing will be stored in low-temperature freezers or liquid nitrogen for drug sensitivity analysis. Blood samples for parasite genomic characterization will be depleted of human white blood cells, and stored frozen. Filter paper blood spots will be dried and stored at room temperature.

To investigate the population genetics of malaria parasites in Cambodia, DNA will be extracted from all filter paper blood spots using a QIAGEN 96 DNA Blood kit (QIAGEN, Valencia, CA). Hemi-nested PCR, using fluorescently end-labeled primers, may be performed to amplify microsatellite loci, unlinked loci distributed throughout the genome (Anderson *et al.,* 1999) and loci from neutral regions of chromosomes 2 and 3 (Su *et al.*, 1999). PCR products will be separated and scored using an automated capillary sequencer.

For genome-wide association analyses and detection of signatures of selection, DNA will be extracted from ~6 mL of WBC-depleted blood collected in EDTA and stored at -20ºC or below using a QiaAmp DNA Blood Midi Kit (QIAGEN, Valencia, CA). Genome-wide screens may be performed using Affymetrix chips containing single nucleotide polymorphisms (SNPs) distributed throughout the *P. falciparum* genome. Copy number variations will also be evaluated using a Nimblegen tiling array. As new genotyping and sequencing technologies become available, they may also be employed for parasite genomic characterization. In addition, culture-adapted parasites will provide a renewable source of DNA, RNA, and protein for genome / proteome-wide screens and expression analysis of parasites with different drug sensitivities.

Parasite diversity will also be determined using both heteroduplex tracking assays (*P. vivax*) and a multi-locus genotyping technique using massively parallel pyrosequencing (*P. falciparum and vivax*). Genotyping of both asexual and sexual parasite stages from DNA stabilized on blood spots will be done to compare transmissibility of specific parasite variants. RNA stabilized on blood spots will be used to assess stage-specific asexual and sexual parasite gene expression. Samples will be analyzed by the University of North Carolina under Cooperative Research and Development Agreement with WRAIR (on file).

Heparinized blood (~ 4 ml) will be cryopreserved with an appropriate cryopreservative solution according to a protocol developed at AFRIMS adapted from MR4 and stored in liquid nitrogen for culture adaptation. Blood samples for in vitro drug sensitivity testing will be stored under assay-appropriate conditions. In vitro drug sensitivity assays will be performed directly at the field sites. The remaining blood will be stored in liquid nitrogen for future drug sensitivity analysis. Blood samples for parasite genomic characterization will be collected in EDTA tubes, depleted of white blood cells, and stored frozen at approximately -20ºC or below. Filter paper blood spots will be dried and stored at room temperature with desiccant packs. For subjects with P. vivax mono-infections, heparinized blood will be collected and used fresh onsite or stored under conditions yet to be determined for development of in vitro culture and/or drug sensitivity methods, or transported to a reference lab covered by this protocol for further method development.

F. Hematology for safety assessment will include the following:

- hemoglobin
- hematocrit
- red blood cell (RBC) count
- cell indices
- platelet count
- white blood cell (WBC) count and differential count
- polymorphonuclear leukocytes (neutrophils)
- lymphocytes
- eosinophils
- monocytes
- basophils

G. Liver and Renal Function Tests for safety assessment will include the following:

- blood urea nitrogen
- creatinine
- total bilirubin
- aspartate aminotransferase
- alanine aminotransferase
- alkaline phosphatase

H. "MSP-1" - Malaria Antibody Analysis – This will include testing for antibodies such as Pf MSP-1 (Merozoite Surface Protein 1) antigen to P. falciparum and/or MSP-3a antigen to P. vivax. Analysis will be conducted at baseline, at study end, and at any time the patient develops blood stage malaria (first infection or a recurrence). Antimalarial antibody levels including Pf (MSP-1) and Pv will be measured in order to assess pre-existing and/or exposure-related antimalarial immunity. Samples will be analyzed by Enzyme-Linked ImmunoSorbent Assay (ELISA) and/or a chemiluminescence-based assay(s). Note that 'MSP-1' is used as an abbreviated synonym for 'malaria antibody analysis' throughout the text of this protocol. However, antibodies to multiple malaria antigens may be assessed under this protocol.

I. Pharmacokinetics (PK) - Plasma samples for determining antimalarial drug levels of piperaquine (2 ml of whole blood per blood draw) will be collected from all volunteers at the specified time points for analysis by high performance liquid chromatography with mass spectrometry (LC-MS) using departmental SOPs for bioanalytical chemistry analysis by LC-MS.

6.10.3 Specimen Labeling and Shipment

All specimens collected during the study will be labeled with the participants study ID number, date, time collected, and test to be performed. Clinical testing including diagnostics and patient safety labs will be performed at the study site (G6PD, hematology, renal and liver function, and microscopy testing). Parasitology testing including parasite culture, *in vitro* resistance, and/or molecular characterization will be performed at AFRIMS

reference lab in Cambodia or Thailand (parasite DNA and additional testing for volunteer safety assessment). Parasite genetic analysis on coded, de-identified samples will be performed at the University of North Carolina under an approved Cooperative Research and Development Agreement with AFRIMS and the WRAIR. The documents are currently on file with the WRAIR Office of Research and Technology Administration Office. A permit to ship samples outside Cambodia under this protocol will be obtained from the National Institute of Public Health, Cambodia (the responsible authority for shipping permits).

Specimens that cannot be analyzed on-site will regularly be shipped from the field to AFRIMS in Bangkok, and/or the University of North Carolina laboratories for further processing and analysis. In the event of remaining specimens, The University of North Carolina laboratories will return all specimens to AFRIMS after all analytic methods have been performed and / or no more than three years after data analysis is complete.

6.10.4 Specimen Storage and Donation for Future Use

Following the completion of laboratory analyses as described in this protocol, any remaining specimens will be stored in a secure AFRIMS or AFRIMS contract facility. During the course of the study, they will be regularly transferred to a secure facility. Only study investigators will have access to the specimens without the explicit permission of the PI. Samples that are unstable may be disposed of after consultation with the Sponsor. The remaining specimens will be stored for approximately 20 years at the Armed Forces Research Institute of Medical Sciences in Bangkok. After the study is completed, residual specimens will only used for purposes outlined in the consent form unless permission for other analyses are granted by the respective IRBs. Volunteers will indicate on the consent form whether or not their samples may be stored and permission granted for future use.

6.11 Data Management

Source data are all information, original records of clinical findings, observations, or other activities in a study necessary for the reconstruction and evaluation of the trial. Examples of these original documents and data records include, but are not limited to, hospital records, clinical and office charts, laboratory notes, memoranda, or evaluation checklists, pharmacy dispensing records, recorded data from automated instruments, copies or transcriptions certified after verification as being accurate and complete, microfiches, photographic negatives, microfilm or magnetic media, x-rays, and volunteer files and records kept at the pharmacy, at the laboratories, and medico-technical departments involved in the clinical study. The study site will maintain appropriate medical and research records for this trial until completion of the study, in compliance with Section 4.9 of ICH E6 GCP, and regulatory and institutional requirements for the protection of confidentiality of volunteers. Source data will be maintained under supervision of the principal investigator for at least 5 years after formal completion of the trial.

All data on the CRF will be entered into a validated database using double data entry. Edit checks will be implemented in the data entry panel to ensure data quality and accuracy. Responses to requests for further clarification of data recorded on the CRF will be answered, dated, and signed by the investigator. Changes will be implemented in the database and the data review and validation procedures will be repeated as needed. All

medication and adverse event information and textual comments will be proofread for consistency between the database and the CRF; the database will be corrected appropriately. In the case when data management is outsourced, the contract organization will be responsible for database quality assurance including, but not limited to, all medication and adverse event terms as described above.

All data and medical information obtained about screened study volunteers will be considered privileged and confidential. Volunteers enrolling in the study will be issued a unique identification code (UIC), which will be used on all study files and clinical sample labels. Individually identifiable volunteer information other than the UIC will not be transcribed on other study documents to include laboratory sample labels, CRFs, nor will it be included in the presentation of study results. The key to the code and documents containing personal information will be kept in a secure location with access restricted to named AFRIMS, CNM and/or RCAF study personnel under control of the Principal Investigator. All personal study volunteer data collected and processed for the purposes of this study will be managed by the investigators and those listed on the delegation of authority log with adequate precautions to ensure the confidentiality of those data, and in accordance with US law and/or applicable local laws and regulations where the requirements exceed those of US law. The study database will be maintained by the Principal Investigators with password-protected access limited to listed investigators. Data management service providers will be provided only coded, de-identified data, which will be processed, analyzed and returned to the investigators in electronic format.

Monitors, auditors and other authorized agents, the United States Army Medical Research and Materiel Command, and the ethics committees approving this research will be granted direct access to the study volunteers' original medical records for verification of clinical trial procedures and/or data, without violating the confidentiality of the volunteers, to the extent permitted by the law and regulations. In any presentations of the results of this study at meetings or in publications, the volunteers' identity will not be revealed.

6.11.1 Source Documents

See section 6.11 above. Source documentation supporting the CRF will indicate the volunteer's participation in the study and will document the dates and details of study procedures, adverse events and volunteer status. Volunteer pre-existing conditions will be recorded in the appropriate sections of the source documentation if they are reported by the volunteer after participation has begun, and the Investigator notified immediately. Pre-existing conditions not reported by the volunteer at the time of enrollment may be grounds for terminating further study participation by the Investigator. Source documents are attached in Appendix E.

6.11.2 Overview of Case Report Forms

Appropriate data will be extracted from the source documents in this study onto case report forms (see Appendix E). The AFRIMS study team will provide the source documents to the site, and will be responsible for completing the CRFs as data is collected. The investigator will ensure the accuracy, completeness, and timeliness of the data reported in the

volunteer's CRF. CRFs will be submitted and approved by the IRBs of Record prior to initiation of the research.

All research data will be collected by the investigator or designee on source documents specifically designed for the purposes of conducting the study. Volunteer clinical and laboratory data for the purpose of providing medical care will be recorded in the appropriate clinic or hospital record using existing forms. Volunteer data necessary for analysis and reporting will be extracted on Case Report Forms specifically designed for that purpose, and entered from the CRFs into a validated database using the services of a commercial data management provider.

6.11.3 Data Compilation

See section 6.11 above.

6.11.4 Disposition of Data

The case report forms, study documentation and a copy of the final report will be stored in an access-controlled place in the contracted archives of AFRIMS. All data will be retained according to ICH guidelines by the Investigators for 5 years at AFRIMS office in Cambodia. After 5 years the Sponsor will be consulted regarding data disposition or continued storage of raw data, which will be at additional cost to the Sponsor.

Volunteer names will also be added to the Volunteer Registry Database as required by the US Army Medical Research and Materiel Command (USAMRMC) whenever human volunteers are used in research studies. This database is maintained only for patient safety and will be kept in a secure location at USAMRMC in Fort Detrick, MD. The purpose of the database is to allow the investigators and/or MRMC regulatory officials to contact volunteers who have participated in US Army biomedical research studies in the event that new information becomes available that could potentially affect volunteer health and/or safety. It is the policy of USAMRMC that data sheets are to be completed on all volunteers participating in research for entry into the U.S. Army Medical Research and Materiel Command Volunteer Registry Database. The information to be entered into this confidential database includes name, address, social security number (where applicable), study name, and dates. The information will be stored at the AFRIMS for a minimum of 75 years. No individually identifiable subject information will otherwise be transported, transmitted or otherwise removed from Cambodia with the exception of information required for the VRD which will be stored at USAMRMC HQ as outlined above.

6.12 Adverse Events

An adverse event is any untoward medical occurrence in a patient or clinical investigation subject administered a pharmaceutical product and which does not necessarily have a causal relationship with this treatment. An AE can therefore be any unfavorable and unintended sign (including an abnormal laboratory finding), symptom, or disease temporally associated with the use of a medicinal (investigational) product, whether or not related to the medicinal (investigational) product.

6.12.1 Collecting Adverse Events

Volunteers treated for malaria with study drug will be carefully monitored for the development of adverse events. For the purposes of this study, adverse events will be assessed and documented from the beginning of study drug administration until discharged from the study, and relationship to study drug assessed. This information will be obtained in the form of open-ended (non-leading) inquiries and from signs and symptoms noted during clinical encounters, observations by study staff, spontaneous reports from volunteers and other sources as appropriate. Specific adverse events will not be solicited in this study. Volunteers will be able to contact study staff through assigned unit liaisons in the event of an emergency.

Study investigators will attempt to establish a diagnosis of the event based on signs, symptoms, and/or other clinical information. Where possible, the clinical diagnosis will be documented as the AE/SAE rather than the individual signs/symptoms. Each adverse event will also be described by its duration (start date, time and duration), an assessment of its cause (e.g. coexisting disease, concomitant medication, or others), its relationship to investigational product (not related, unlikely, possibly, probably, definitely), and whether it required specific therapy.

The investigator will also make an assessment of severity for each AE reported during the study. The assessment will be based on the investigator's clinical judgment. An AE that is graded as severe should not be confused with a serious adverse event (SAE). The severity of each adverse event must be recorded as 1 of the choices on the following scale:

- Mild - No limitation of usual activities
- Moderate - Some limitation of usual activities
- Severe - Inability to carry out usual activities

For each adverse event, the relationship to the study drug must be recorded as 1 of the choices on the following scale:

- Definite - Causal relationship is certain (ie, the temporal relationship between drug exposure and the adverse event onset/course is reasonable, there is a clinically compatible response to dechallenge [a rechallenge procedure may be used, if necessary], other causes have been eliminated, and the event is definitive pharmacologically or phenomenologically)

- Probable - High degree of certainty for causal relationship (ie, the temporal relationship between drug exposure and the adverse event onset/course is reasonable, there is a clinically compatible response to dechallenge [rechallenge is not required], and other causes have been eliminated or are unlikely)

- Possible - Causal relationship is uncertain (ie, the temporal relationship between drug exposure and the adverse event onset/course is reasonable or unknown, dechallenge/rechallenge information is either unknown or equivocal, and while other potential causes may or may not exist, a causal relationship to the study drug does not appear probable)

- Unlikely - Not reasonably related (ie, while the temporal relationship between drug exposure and the adverse event onset/course does not preclude causality, there is a clear alternate cause that is more likely than the study drug to have caused the adverse event)

- Not Related - No possible relationship (ie, the temporal relationship between drug exposure and the adverse event onset/course is unreasonable or incompatible, or a causal relationship to study drug is implausible)

6.12.2 Documenting Adverse Events

An adverse event (AE) temporally related to participation in the study will be documented whether or not considered to be related to the test article. This definition includes intercurrent illnesses and injuries and exacerbations of preexisting conditions. All adverse events will be recorded based on their frequency, severity, and relationship to study medication in accordance with current AFRIMS/WRAIR SOP. These indices of safety and tolerability among treatment groups will be compared using each patient as the unit of analysis. Adverse events will be documented in the volunteer source documents and case report forms.

Adverse events will be classified by the severity (intensity) of an adverse event as mild, moderate or severe. Causality of an event will be classified as definitely related, probably related, possibly related, unlikely related, or unrelated based on its apparent the relationship to the research intervention. Adverse events will be assessed and recorded by study investigators or their designees. An Investigator will review all causality and severity assessments, with final review and determination by the Principal Investigator if uncertainty remains. The Medical Monitor will review all Serious and Unexpected AE determinations.

6.12.3 Expected Adverse Events

All AEs occurring during the course of the clinical trial, defined as from the moment of first antimalarial treatment administration until discharge from the study, will be collected, documented, and graded by study investigators. Symptoms present at enrollment will not be classified as AEs, but any new symptoms or signs occurring after this time would constitute adverse events.

For this study, AEs will include events reported by the volunteer, as well as clinically significant abnormal findings on physical examination or laboratory evaluation. A new illness, symptom, sign or clinically significant clinical laboratory abnormality or worsening of a pre-existing condition or abnormality is considered an AE. Stable chronic conditions, such as arthritis, which are present prior to clinical trial entry and do not worsen are not considered AEs.

Adverse events that occur will be treated as clinically indicated where appropriate. The most likely adverse event that will occur during the study is malaria infection. Malaria infection or reoccurrence will be actively sought by the study team, and all suspected cases will be referred immediately for further evaluation and treatment as described in the

protocol. It is expected that active detection and treatment by a dedicated team will lead to earlier diagnosis and initiation of appropriate therapy, potentially reducing the rate of more severe illness.

6.12.4 Serious Adverse Events and Unexpected (Unanticipated) Adverse Events

A serious adverse event (SAE) is defined as any adverse experience occurring during study participation that results in any of the following outcomes: death, a life-threatening adverse drug experience, inpatient hospitalization or prolongation of existing hospitalization, a persistent or significant disability/incapacity, or a congenital anomaly/birth defect. Important medical events that may not result in death, be life-threatening, or require hospitalization may be considered a serious adverse drug experience when, based upon appropriate medical judgment, they may jeopardize the patient or volunteer and may require medical or surgical intervention to prevent one of the outcomes listed in this definition.

An SAE will be defined in this study as any untoward medical occurrence regardless of cause or relationship to study drug that:

- Results in death.
- Is life-threatening. Any adverse experience that places the volunteer, in the view of the investigator, at immediate risk of death from the reaction as it occurred (i.e., it does not include a reaction that, had it occurred in a more serious form, might have caused death).
- Requires in-patient hospitalization or prolongation of existing hospitalization (excluding the hospitalization required by the study for the period required to treat malaria and any associated co-morbidities).
- Results in persistent or significant disability or incapacity.
- Is a congenital anomaly/birth defect.
- An event that requires urgent medical intervention to prevent permanent impairment or damage.
- Important medical events that do not result in death, are not life-threatening, or do not require hospitalization may be considered serious adverse events when, based upon appropriate medical judgment, they might jeopardize the volunteer and might require medical or surgical intervention to prevent one of the outcomes listed above.

An unexpected or unanticipated event involving risks to volunteers or others is one that is not described as a risk with respect to nature, severity, or frequency in the protocol and/or informed consent form. An unexpected adverse event is further defined as any adverse drug effect, the specificity or severity of which is not consistent with that which has been previously reported in the current published literature, or described in the study documents.

6.12.5 Adverse Event Reporting

Expected adverse events will be reported on a routine basis to the responsible IRBs and the Sponsor by the investigator as part of scheduled Continuing Review Reports as stipulated by the IRB.

ICH GCP 6, Section 4.11 require that an investigator notifies the sponsor, regulatory authority(ies) and the local IRB immediately of any serious adverse event, deaths, or life-threatening problems that occur in the study. Unanticipated problems involving risk to volunteers or others, serious adverse events related to participation in the study and all volunteer deaths will be promptly reported within 48 hours after the PI/study team has knowledge of the event to the NEHCR, Cambodia National Ethics Committee for Health Research, as well as by phone or by facsimile to the Walter Reed Army Institute of Research Division of Human Volunteers Protection. A complete written report will be sent within 10 business days to the Director, Office of Research Management, Walter Reed Army Institute of Research Division of Human Volunteers Protection. In addition, the complete report will be sent by WRAIR DHSP to the U.S. Army Medical Research and Materiel Command, and also to the National Ethics Committee for Health Research, Cambodia.

All safety reports for events that are both serious and unexpected at a minimum will include Volunteer identification number and initials, associate investigator's name and name of MTF, volunteer's ages, gender and ethnicity, test article and dates of administration, signs/symptoms and severity, date of onset, date of resolution or death, relationship to the study drug, action taken, concomitant medication(s) including dose, route and duration of treatment, and date of last dose. Reports will be submitted according to current human volunteer's protections SOPs of AFRIMS and the WRAIR.

The medical monitor will review all unanticipated problems involving risk to volunteers or others, serious adverse events, and all volunteer deaths associated with the protocol and provide an unbiased written report of the event. At a minimum, the medical monitor will comment on the outcomes of the event or problem, and in the case of a serious adverse event or death, comment on the relationship to participation in the study. The medical monitor will also indicate whether he/she concurs with the details of the report provided by the study investigator. Reports for events determined by either the investigator or medical monitor to be possibly or definitely related to participation and reports of events resulting in death should be promptly forwarded to the WRAIR IRB.

All serious adverse events regardless of treatment group or suspected relationship to study drug must be reported within 24 hours of the event by telephone to the named local Medical Monitor for the study, as well as the Sponsor's Medical Expert (SME). The Medical Monitor and SME will review all unanticipated problems involving risk to volunteers or others, serious adverse events and all volunteer deaths associated with the protocol and provide an unbiased written report of the event to the USAMRMC Office of Research Protections (ORP) Human Research Protection Office (HRPO). At a minimum the Medical Monitor and SME will comment on the outcomes of the event or problem and in the case of a serious adverse event or death comment on the relationship to participation in the study. The Medical Monitor and SME will also indicate whether he/she concurs with the details of the report provided by the study investigator. Reports for SAEs and unexpected adverse events determined by either the investigator or medical monitor to be probably or definitely

related to participation and reports of events resulting in death should be promptly forwarded to the WRAIR IRB, the USAMRMC ORP HRPO, and to the National Ethical Committee for Health Research, Cambodia.

The investigator will complete a Serious Adverse Event Form within the following timelines:

- All deaths, whether associated or not associated, will be recorded on the Serious Event Form and sent by fax within 24 hours of site awareness of the death.
- Serious adverse events other than death, regardless of relationship, will be reported via fax by the site within 48 hours of becoming aware of the event.

6.12.6 Follow-up of Adverse Events

All AEs regardless of severity will be followed by study investigators until satisfactory resolution. Resolution could include a classification of ongoing if the event is stabilized with no further change expected. The investigator will ensure that follow-up includes any supplemental investigations as may be indicated to elucidate the nature and/or causality of the AE or SAE. This may include additional laboratory tests or investigations, histopathological examinations, or consultation with other health care professionals.

If a study volunteer withdraws from the study or if an investigator decides to discontinue the volunteer from the study because of a SAE, effort will be made to ensure the volunteer has appropriate medical follow-up. Monitoring will continue where possible and appropriate in order to determine whether the problem prompting hospitalization has resolved or stabilized with no further change expected, or is discovered to be clearly unrelated to study drug, or progresses to death. The Investigator/clinical staff will report the follow-up for serious adverse events as noted above.

After discharge from the Cohort Study, any treatment-related adverse events classified as "probable" or "definite" in relation to that are likely to will be followed to resolution at 30 day intervals where possible. All SAEs will be followed until satisfactory resolution or until the Principal Investigator (with agreement of the Medical Monitor) deems the event to be chronic or the patient to be stable.

A post-study AE/SAE is defined as any event that occurs after the volunteer has been discharged from the Cohort Study. Investigators are not obligated to actively seek AEs or SAEs in former study participants. However, if the investigator learns of any SAE, including a death, at any time after a volunteer has been discharged from the study, and he/she considers the event reasonably related to the study, the investigator will promptly notify any IRB.

6.13 Withdrawal Criteria

Any volunteer may be discontinued from the study at any time at the discretion of the Principle Investigator or designee, medical monitor, a consulting clinical physician, or a

responsible IRB if he/she feels it is in the best interest of the volunteer or if in the judgment of the investigator continuing in the study would be harmful and/or inappropriate for the volunteer (e.g. patients not tolerating the study treatment, development of SAEs or if a patient cannot be followed thereby not permitting adequate safety assessment). Any patient who is terminated due to an SAE or determined to have an unexpected AE will be reported to the medical monitor for review. There are no pre-defined criteria for study withdrawal as this study is using interventions that have been widely tested and reported in the published literature.

Volunteers who withdraw from the study or are terminated will be asked to undergo a final safety assessment, and complete discharge procedures and assessments. Volunteers will be allowed to withdraw from the study at any time without prejudice or loss of benefits to which they are entitled. Volunteers may be removed from the study by a study investigator or the medical monitor should their continued participation be injurious to their health and well-being at any time. The NEHCR and the WRAIR IRB will be notified when a volunteer is withdrawn from the study as part of the continuing review report, unless withdrawl is the result of an SAE.

6.14 Rescue Treatment for Malaria Infection Not Responding to Initial Therapy

The requirement for rescue treatment will be based on investigator clinical judgment. However, failure to respond adequately to DP will include the following: development of danger signs (e.g. impaired consciousness, convulsions, respiratory distress) or severe malaria in the presence of parasitemia; and parasitemia on Day 2 higher than Day 0. The presence of both asexual parasitemia and fever (tympanic temperature > 38°C) on Day 3 is not necessarily an indication for rescue treatment as long as parasite counts continue to trend downward, and the patient is without danger signs as outlined in the National Guidelines, (see Section 3.1.3, Appendix B). Per protocol, subjects will remain under direct observation until both parasitemia and fever have cleared.

Recent AFRIMS clinical studies in this region have demonstrated that the majority of malaria patients with both fever and parasitemia at 72 hours who continue their antimalarial treatment will go on to clear parasites and fever and remain free from recurrence up to 42 days (ARC1 – Noedl *et al*, in press; and ARC2 studies – Fukuda *et al*, unpublished), and it is important to capture this outcome. Treatment of subjects who fail the primary treatment regimen with worsening symptoms, fever, and/or parasitemia after day 4 will be given malaria rescue therapy in accordance with current National Treatment Guidelines (Appendix B, Section 4.3.1). In this case, recommendations for the use of A+M5 will be replaced with DP as the first line ACT. Patients who develop worsening or unexplained symptoms not otherwise attributable to malaria at any time will be evaluated for alternative diagnoses.

6.15 Criteria for Study Termination

See section 6.13 above. There are no pre-defined criteria for study termination as this study will use interventions that have been widely tested and reported in the published

literature. Volunteers who withdraw from the study or are terminated will be asked to undergo a final safety assessment, and complete discharge procedures and assessments.

6.16 Quality Control and Quality Assurance

AFRIMS maintains approved SOPs/SSPs that govern QC/QA procedures that will be followed during the course of this study. The study will be monitored by an external contracted clinical monitor with extensive experience in monitoring AFRIMS studies in Southeast Asia, contracted to USAMMDA. This study does not involve an IND.

7. STATISTICAL METHODS

7.1 Statistical Procedures

The following describes the analyses (primary) for the cohort and treatment studies.

Cohort Study (Primary Analysis)

The malaria attack rate (AR) (with exact 95% confidence intervals) will be reported as the number of Volunteers developing a confirmed malaria infection divided by the number of Volunteers that either remain enrolled to study completion or develop malaria and are treated. To take into account any loss to follow-up, Kaplan-Meier estimates (plots) of cumulative malaria risk will also be presented. Crude ARs (95% CI) and Kaplan-Meier estimates of cumulative malaria risk will be reported for individual species, as well as for all species combined.

Treatment Study (Primary Analysis)

Volunteers developing malaria infection during the active observation follow-up period will be treated as part of a two arm, randomized, open label treatment study comparing the efficacy (42 day PCR-corrected malaria recurrence), safety, tolerability and pharmacokinetics of a two versus three day course of Dihydroartemisinin-Piperaquine (DP). The primary end-point will be comparative 42 day malaria cure rates. Estimates of treatment cure rates and 95% CIs (exact) will be reported. Fisher's exact test (two-sided) will be used to test the null hypothesis of no difference in cure rates. A p-value < 0.05 (two-sided) will be considered statistically significant.

7.2 Sample Size Estimation

Cohort study

Up to 200 Volunteers will be enrolled and followed weekly for an estimated 3-5 month period. A major purpose of the cohort follow-up study is to estimate the malaria attack rate (AR). An evaluation of the effect of the number of persons followed (N) on the precision (margin of error) of the resulting estimated AR was calculated. Based on preliminary data collected on potential sites, the resulting expected AR estimate from a follow-up period of 4 months is 40%. With a cohort N = 200, then the margin of error under the assumption of an attack rate of 40% is 6.79%. In this case the 95% CI (normal distribution approximation) of the AR estimate would be 40% ± 6.79% (33.21% to 46.79%)].

Treatment study

The exact length of follow-up of the prospective epidemiology cohort will be determined by the number of Volunteers developing malaria to ensure that there is adequate power (80% nominal) to compare the relative efficacy of the two treatment groups. In prior published studies using DP in Southeast Asia, efficacies of the two regimens are estimated to be roughly 75% for a 2 day regimen and 98% for a 3 day regimen respectively (Krudsood, 2007). Based on Fisher's exact test and a type-one error rate of 5% (two-sided), a total sample size of 76 (2 arms with 38 Volunteers each) is required to achieve the required nominal 80% power [actual power = 81%; PASS (2005)] to distinguish the expected difference in efficacy of 23 percentage points (98% - 75%). Attack rates are difficult to predict without first conducting a prospective cohort study. Therefore, we estimate that it will take up to 4 months for 40% of Volunteers enrolled (n = 0.40 x 200 = 80) to develop clinical malaria, and be treated by the study team using an active case detection and

treatment approach. Adequate power will be reached when 76 Volunteers have been randomized to uncomplicated malaria treatment (38 Volunteers per group). Therefore, Volunteers in the Cohort Study will be followed until up to 80 Volunteers have been randomized to uncomplicated malaria treatment (40 per group), which allows for two dropouts per group. Based on preliminary epidemiology data and statistical considerations, this will take approximately 3-5 months, depending on attack rates encountered. With N = 200 and assuming an expected four month cumulative attack rate of 40% (50% five month), the probability of enrolling at least 76 cases within 5 months is > 0.99 (based on binomial distribution). If the attack rates is substantially lower than expected, (assuming at least a 30% cumulative attack rate over 4 months, or 37.5% over five months), the probability of having to follow Volunteers for more than 5 months to enroll at least 76 malaria patients is 0.53. If the total number of cases enrolled in five months is at least 70 then the power will be ≥75%. If the number of cases is at least 66, power will be ≥ 71%.

7.3 Randomization and Stratification

During the Treatment Study, volunteers developing malaria will randomized into the two open label treatment arms using block randomization with a block size of two. There will not otherwise be randomization used in this study. Data may be stratified post-hoc based on demographic and/or other variables, but stratification is not part of the primary analysis.

7.4 Populations for Analysis

Cohort Study – All volunteers enrolled and completing at least one follow-up visit will be included in the Primary Endpoint analysis of the malaria attack rate.

Treatment Study - All volunteers with a diagnosis of uncomplicated malaria who receive at least one dose of test article during the Treatment Study will be included in the efficacy database for Primary Endpoint analysis (Intention to Treat). The per protocol analysis population will include all those volunteers who completed the full prescribed treatment course of DHA-piperaquine. However, volunteers completely lost to follow-up that do not complete 42 days worth of assessments will be excluded from the per protocol efficacy analysis. All volunteers with at least one follow-up assessment will be included in the safety analysis, pharmacokinetic analysis, and MSP-1 antibody titer analysis. All parasitologic data will be included in the parasitologic analysis. The safety analysis database will include those volunteers in the set of randomized volunteers who receive at least 1 dose of study drug.

7.5 Deviations from the Statistical Plan

Deviations from the statistical plan, along with the reasons for the deviations, will be described in protocol amendments, the complete statistical plan, the clinical study report, or any combination of these, as appropriate.

8 DATA ANALYSIS

8.1 General Considerations – Data Analysis

The general purposes of the data analyses for the Cohort and Treatment study are as follows.

Cohort Study:

(1) Estimate the malaria attack rates (AR) to help guide future studies (Primary).

(2) Describe the demographic and epidemiological characteristics of the cohort at baseline and during follow-up. Describe baseline/follow-up laboratory safety variables (hematology, liver, renal, etc.)

Treatment Study (efficacy of 2 versus 3 day regimens of DP):

(1) Assess/test the efficacy (recurrence rates at 42 days) of the two regimens (Primary)

(2)Describe/compare the safety, tolerability and pharmacokinetic data in the 2 versus 3 day treatment regimens

(3) In patients with malaria, investigate parasite genomics in different regions of Cambodia and define genomic regions associated with resistance

8.2 Cohort Study Analysis

Demographic, epidemiological, and laboratory data will be summarized at baseline and for any data collected at follow-up. Appropriate univariate statistics will be calculated to summarize and compare the range, location (means, medians, etc), and variability of numerical data. Geometric means will be calculated for antibody titer data, and all non-normally distributed data. Graphical summaries (e.g. box plots, histograms) will be used to describe and compare distributions of numeric variables. Frequency tables will be used to summarize distributions for discrete data. Confidence limits (95%) for means, geometric means and proportions will be calculated.

The primary endpoint for the Cohort Study is the all species malaria attack rate (AR) based on the first occurrence of any confirmed malaria infection during the period of follow-up (4 months). The malaria attack rate (AR) from the Cohort Study will be calculated as the number of Volunteers developing a confirmed malaria infection divided by the number of Volunteers that either remain enrolled to study completion, or develop malaria and are treated.

Cumulative risk (AR) estimates (95% CIs) will also be calculated for individual species, as well as all species combined. Exact (binomial) 95% confidence intervals will be reported for all AR estimates. To take into account any loss to follow-up, Kaplan-Meier estimates (plots) of cumulative malaria risk will also be presented (individual species and all species).

8.3 Efficacy Study Analysis

Primary endpoint treatment efficacy is defined as PCR-corrected parasitological cure of malaria at 42 days after starting therapy. Efficacy against all blood stage malaria infection will also be classified according to WHO malaria treatment outcome classifications adapted for the purposes of clinical research (WHO 2009) as a secondary endpoint. Note however that clinical management of these individuals will be based on the best clinical judgment of the investigators, informed by current National Treatment Guidelines in Cambodia (see Appendix B).

Treatment Outcome	Symptoms and Signs
Early treatment failure	- Development of danger signs or severe malaria on days 1-3 in the presence of parasitemia.<br>- Parasitemia on day 2 higher than the Day 0 count irrespective of temperature.<br>- Parasitemia on day 3 with tympanic temperature ≥38.0°C.<br>- Parasitemia on Day 3 that is ≥25% of the count on day 0.
Late treatment failure:<br>- Late clinical failure	- Development of danger signs or severe malaria after day 3 in the presence of parasitemia, without previously meeting any of the criteria of ETF.<br>- Presence of parasitemia and tympanic temperature ≥38.0°C (or history of fever) on any day from days 4-42, without previously meeting any of the criteria of ETF.
- Late parasitological failure	- Presence of parasitemia on any day from days 7-42 and tympanic temperature <38.0°C, without previously meeting any of the criteria of ETF or LCF.
Adequate clinical and parasitological response	- Absence of parasitemia on day 42 irrespective of tympanic temperature without previously meeting any of the criteria of ETF, LCF or LPF.

Efficacy at Day 42 will be based on microscopy of thick/thin blood films by expert microscopists blinded as to treatment allocation and clinical data of each volunteer following AFRIMS SOP. The basic statistical methods cited above for the Cohort Study will be used to summarize and compare the demographic, epidemiological, and laboratory data (hematology, hepatic, renal, etc) for the two treatment groups (baseline and follow-up).

Primary Analysis – Treatment Study

The primary efficacy analysis for comparing 2 vs. 3 day treatment with DHA-piperaquine will be by intention to treat. The primary endpoint (cure) for the treatment portion of the study is defined as non-recurrence of malaria (within 42 days) following treatment as detected by PCR-corrected microscopy. Cure rates (exact 95% CIs) will be calculated. Fisher's exact test (two-sided) will be used to test the null hypothesis of no difference in cure (fail) rates. A p-value < 0.05 (two-sided) will be considered statistically significant. As supporting analysis, a log- rank test will be performed to compare time to treatment failure in the two treatment regimens.

Secondary Analyses –Treatment Study

The above primary (and supporting) efficacy analyses will be done for the per protocol population.

All clinical and laboratory data related to secondary endpoints (Section 5.2) will be summarized and compared in the two regimens. T-tests and chi-square tests will be used to assess the statistical significance of differences in two means (possibly log-transformed) or proportions. Confidence limits (95%) for means, geometric means and proportions will be calculated. Time to event data (e.g. fever and parasite clearance time) will be summarized using Kaplan-Meier plots. The log-rank test will be used to assess the statistical significance of difference between treatment groups. The time required to achieve a reduction in parasite density of 50%, 90%, and to undetectable levels will be analyzed using Cox proportional hazard modeling. Curve fitting will be used to interpolate results for calculation of parasite clearance and parasite density reductions

Nominal data, including occurrence of adverse events (AE), will be expressed as percentages and compared with chi-square tests. Ordinal data will be compared using rank (non-parametric) methods. With the exception of AEs (see Section 8.7), nominal significance levels of $p < 0.05$ will be considered statistically significant for pre-planned comparisons. Correlations between clinical and laboratory parameters will be explored and represented graphically. Other exploratory analyses may be carried out.

Cumulative incidence of primary *P. vivax* infection, relapse and recurrence will be calculated. Since there is little recent experience with 8-aminoquinoline treatment for radical cure of *P. vivax* in Cambodia, this portion of the study is purely exploratory for the purposes of further defining the epidemiology of *P. vivax* in this setting, and not intended to calculate the true efficacy of either primaquine regimen.

8.3 Pharmacokinetic Analysis

Drug levels (piperaqunie) at each of the time points will be expressed as means and 95% confidence intervals if the data are normally distributed, and compared using parametric tests. If not normally distributed they will be expressed as medians and the range and interquartile range given. Comparison will be by non-parametric tests. Standard pharmacokinetic parameters including C_{max}, T_{max}, $T_{1/2}$ and AUC will be calculated using WinNonLin and/or other appropriate microcomputer software packages. Exploratory

analyses will be undertaken to describe the relationship between plasma concentration and the effect of DP treatment on pharmacodynamic variables over time. If the data allow, an attempt will be made to characterize the relationship using modeling.

8.4 Parasite drug resistance in vitro

For in vitro data from patients with P. falciparum infections, inhibitory concentrations at 50% (IC50) and 90% (IC90), the principal measures of drug sensitivity, will be estimated by non-linear regression analysis of the raw data obtained from the ELISA plate reader or the liquid scintillation counter (Noedl 2002). ICs and other continuous variables will be summarized using geometric means with 95% confidence intervals. Comparison of activity will be done by comparing individual ICs by Mann Whitney U-test analysis. Comparison of results obtained using different methods will be done by correlation analysis and Bland-Altman plots (Bland and Altman 1995).

8.5 Molecular Markers of Parasite Resistance

Molecular analysis will be performed in order to determine the extent of genetic diversity in parasites collected from different regions of Cambodia. Cryopreserved blood will undergo culture adaptation and in vitro drug sensitivity testing using established methods. Parasite DNA will be extracted from human white blood cell -depleted blood samples and from filter paper blood spots and subjected to genotyping using various platforms including but not limited to direct DNA sequencing, next-generation DNA sequencing, and DNA and RNA chip technologies, to identify specific parasite genes or genetic loci associated with resistance.

Molecular analyses, to include genome-wide DNA sequencing, PCR, multi-locus genotyping using massively parallel pyrosequencing (MPP), and other molecular analyses as developed, will be performed on isolated parasites. We will compare the level of diversity in each geographic location using ANOVA or non-parametric tests. Samples will be analyzed by the University of North Carolina under Cooperative Research and Development Agreement with WRAIR (on file).

In particular, we will conduct a GWAS of up-selected parasites in order to identify genetic loci associated with antimalarial drug resistance. Parasites from patients may be culture adapted. Using the multi-locus genotyping, specific variants (both resistant and non-resistant) will be isolated and have their entire genome sequenced using a Solexa sequencer or interrogated by Affymetrix chips.

We will identify specific up-selected variants due to dihydroartemisinin (DHA) exposure by conducting multi-locus genotyping using MPP. We will use patient samples collected over the first 72 hours of treatment with DHA/piperaquine with parallel drug resistance phenotypic characterization *in vitro*. This will allow us to measure molecular markers of selection. We will define selection coefficients for variants up-selected by DHA and/or piperaquine based on in vitro resistance profiles. Using data on parasite density and change in parasite frequency, selection coefficients will be determined. This will be the first

time these will be determined *in vivo*. We will also model the in-host dynamics of selection of DHA resistant parasites *in vitro* using culture adapted parasites.

8.6 Safety Analysis

The safety analysis set will be used for all safety analyses. The overall safety and tolerability of DHA-piperaquine treatments will be assessed throughout the study by evaluating adverse events and the following additional safety variables:

- Clinical laboratory tests (liver function, renal function, and hematology)
- Vital signs
- ECG findings
- Physical exam findings

For continuous variables, descriptive statistics (n, mean, standard deviation, median, minimum and maximum) will be provided. For categorical variables, volunteer count and percentage will be provided. Descriptive summaries of serious adverse events, volunteer discontinuations due to adverse events, and potentially clinically significant abnormal values (clinical laboratory or vital signs) will also be provided. Adverse events will be attributed to the treatment regimen corresponding to the last dose administered.

Adverse events (AE), will be expressed as percentages and compared with chi-square tests. AE rates are expected to be small and the study is not powered to detect differences in AE rates. Because of the large number of statistical tests, p-values will not be used to assess "statistical significance, but to flag differences in AE rates. Differences in AE rates will be flagged if $p < 0.05$ and identified as possibly clinically important if $p < 0.01$.

8.7 Interim Analysis

No interim analysis is planned for this study.

9. ETHICAL CONSIDERATIONS

The investigator will ensure that this study is conducted in full conformity with the current revision of the Declaration of Helsinki (Fifth revision October 2000), or with the International Conference for Harmonization Good Clinical Practice (ICH-GCP) regulations and guidelines, whichever affords the greater protection to the volunteer.

9.1 Informed Consent

Freely given informed consent will be obtained from every volunteer prior to study participation. Informed consent will take place before any study specific procedure, prior to the initiation of non-routine study-related tests, and prior to administration of study drug. Signed and dated, informed consent will be obtained from each volunteer in accordance with GCP and with local regulatory and legal requirements. The completed informed consent form must be retained by the investigator as part of the study records and a copy will be provided to study volunteers. The investigators, or a person designated by the investigators, will fully inform the volunteer of all pertinent aspects of the trial including the written information given approval/favorable opinion by the IRB/IEC. Neither the investigator, nor the trial staff, will coerce or unduly influence a volunteer to participate or to continue to participate in the study.

In obtaining and documenting informed consent, the investigators will comply with the applicable regulatory requirement(s), and will adhere to GCP and to the ethical principles that have their origin in the Declaration of Helsinki (5th revision, 2000). Prior to the beginning of the trial, the investigators will have the IRB/IEC's written approval/favorable opinion of the written informed consent form and any other written information to be provided to volunteers.

The written informed consent form and any other written information to be provided to volunteers will be revised whenever important new information becomes available that may be relevant to the volunteer's consent. Any revised written informed consent form, and written information will receive the IRB/IEC's approval/favorable opinion in advance of use. The volunteer will be informed in a timely manner if new information becomes available that may be relevant to the volunteer's willingness to continue participation in the trial. The communication of this information will be documented.

9.2 Volunteer Identification and Confidentiality

All personal study volunteer data collected and processed for the purposes of this study will be managed by the investigators and his/her staff with adequate precautions to ensure the confidentiality of those data, and in accordance with applicable national and/or local laws and regulations on personal data protection.

Volunteers will not be identified in any presentation of the results. Note that complete confidentiality cannot be promised to military volunteers because information bearing on the military volunteer's health may be required to be reported to the volunteer's appropriate medical or command authority within Cambodia. Confidentiality risk assessment for military personnel requires serious consideration of the potential to affect the military

career. In military personnel, it is possible that medical and psychological diagnoses can lead to limitation of duties or discharge. Information regarding alcohol or drug abuse, drunk driving, sexual or spousal abuse and sexual orientation may lead to adverse personnel actions or possible legal action. For aviators, loosing flight status due to a physical or psychological concern is an issue, and can lead to reduction in pay. For military volunteers, the consent form will contain a statement that complete confidentiality cannot be guaranteed because information bearing on a soldier's health may be required to be reported to appropriate medical or command authorities.

This study will not involve the collection of data on sensitive matters such as sexual behavior or criminal activities. No HIV or human genetic testing will be performed on any samples collected during this study. This protocol does not involve audio or videotaping of research volunteers. All volunteer records and CRFs will be carefully designed to limit the personal information to be acquired to that which is essential. Data that could reveal a volunteer's identity will be stored in files accessible only to authorized staff. As early as feasible, the data will be coded to remove identifying information.

The database(s) generated by this study will contain only information collected through CRFs and laboratory data. The database will be password protected to limit access to the data. No individually identifiable information will be included in the database. The patient enrollment log containing individually identifiable information will be kept separately in a secure place under control of the RCAF and will not be accessible for the purposes of data analysis. The Department of Immunology and Medicine may access the enrollment log on an as-needed basis for the purposes of auditing, verification and/or quality control only. Geographic locations will be coded and codes maintained by RCAF to preserve operational security. AFRIMS and CNM investigators will use this information only for the purpose of data analysis.

The database will be constructed by a commercial data management provider, and will include double data entry, edits checks, and automated query generation to ensure the integrity of data. The study database will be maintained at AFRIMS once complete. Data will be shared with the investigators in Cambodia. The site will permit authorized representatives of AFRIMS and regulatory agencies, to include the Cambodian NEHCR, the MRMC, Human Research Protection Office (HRPO), and WRAIR IRB to examine (and when required by applicable law, to copy) clinical records for the purposes of quality assurance reviews, audits and evaluation of the study safety and progress.

Volunteer names will also be added to the Volunteer Registry Database as required by the US Army Medical Research and Materiel Command (USAMRMC) whenever human volunteers are used in research studies. This database is maintained only for patient safety and will be maintained in a secure facility at AFRIMS. It is the policy of USAMRMC that data sheets are to be completed on all volunteers participating in research for entry into the U.S. Army Medical Research and Materiel Command Volunteer Registry Database. The purpose of this secure database is to notify volunteers of any safety concerns that may arise at any time in the future as a result of their participation in the research study. The information to be entered into this confidential database includes name, address, social security number, study name, and dates. The information will be stored for a minimum of 75 years.

9.3 Risk to Volunteers and Precautions to Minimize Risk

The main risks to individual volunteers as a result of study participation beyond those related to the clinical diagnosis and treatment of malaria include:

- Adverse effects from treatment with antimalarials. DHA-piperaquine is currently the standard of care of RCAF beneficiaries who will be participating in the study. The study is designed in part to evaluate the safety and tolerability of this regimen. The other potential antimalarials used in this study will all be prescribed in accordance with the study protocol and current national treatment guidelines. In general, potential medications used to treat malaria are well tolerated. Volunteers will be followed by a trained team of clinical malaria researchers with particular attention to potential side effects, and study treatment will be directly observed. Volunteers in this study will be followed up more closely and for longer duration by a dedicated study team, and will have an enhanced level of care compared to malaria patients receiving standard of care in Cambodia (non-DOT and more limited follow-up visits).

Phlebotomy can cause discomfort and pain at venipuncture sites. Volunteers will be counseled to return to the clinic if local infection is suspected. Healthy volunteers in the cohort study will have approximately ~22 mL drawn over the course of the study. The total volume of blood drawn in this study for patients who are admitted and treated for malaria as part of the Treatment Study will be up to approximately ~123 ml over 42 days. The maximum draw on any day will be on Days 0 of treatment when ~23 ml will be drawn. Subjects treated for recurrences of malaria, or treated for severe malaria will have ~20-25 mL drawn for study-mandated laboratory procedures, plus additional blood for clinical management if warranted based on the judgment of the treating physician. Volunteers with G6PD deficiency who are referred for primaquine therapy will have an additional ~12-16 mL drawn during treatment with primaquine therapy to monitor for potential hemolysis.

Piperaquine can cause prolonged QT interval. While other research studies have already shown that DP at the doses used in this study does not cause a significant prolongation of QT interval, daily EKGs during treatment with piperaquine, and weekly for 4 weeks after completion will be performed to minimize risk. There is also a minimal risk of skin irritation from the adhesive pads used for EKG testing which is typically self-limited.

9.4 Alternatives to Test Article (or Research Treatment)

Volunteers may elect not to participate in the study, and receive standard medical care for malaria which currently includes 2-3 days of non-DOT therapy with an artemisinin-piperaquine or an artesunate-mefloquine combination. Volunteers with access to civilian healthcare may also be able to receive national standard of care therapy from the government sector with artesunate and mefloquine (see Background), or other regimens (some unlicensed and unregulated) from the private sector.

9.5 Benefits to Volunteers

Volunteers will benefit from the increased vigilance provided by the study team to ensure active malaria detection and prompt treatment. Data provided by RCAF indicates that the vast majority of adverse disease outcomes to its deployed personnel result from delays in diagnosis and treatment. Volunteers will also benefit from directly observed malaria treatment and careful follow-up by a trained study team. There are no other direct benefits to volunteers from participating in this study. The collective benefit of improved malaria control in the areas studies, and throughout RCAF AORs is the purpose of conducting this study. The study will benefit the community as a whole by providing up-to-date information on drug resistance and treatment regimen efficacy which will be provided to the RCAF Health Department, and other appropriate Cambodian government authorities.

9.6 Risks to Study Personnel and Precautions to Minimize Risk

There are no additional anticipated risks to study personnel as a result of study participation. AFRIMS SOPs on occupational health and safety will be adhered to at all times, and all staff certified at the appropriate level. Universal precautions will be observed at all times when handling biological specimens.

9.7 Risks to the Environment

None.

9.8 Financial Incentives to Volunteers

Compensation will be provided throughout the study, and volunteers will be compensated for all study visits completed if they leave the study prior to completion. The estimated compensation for completion of the trial will be approximately 10,000 Cambodian Riel (approximately US $2.50 depending on current exchange rates) per completed study visit including screening and enrollment, and unscheduled visits. Volunteers will also receive this same amount of compensation on a daily basis while hospitalized, should they develop malaria. This compensation takes into consideration lost earnings (for civilian dependent beneficiaries), meals and incidentals arising from participation, and discomfort from phlebotomy. Compensation provided in the study will be outlined in the Informed Consent Document which will be the definitive document detailing volunteer compensation throughout the study. All changes in compensation made to the current IRB-approved Informed Consent Document will be definitive, and will supersede the details provided in this section.

9.9 Medical Care for Injury or Illness

Medical care in case of research-related injury (including long-term medical care) will be provided free of charge according to local standard of care at Cambodian military

healthcare facilities or government referral hospitals as appropriate. Volunteers are authorized all necessary medical care for injury or disease which is the proximate result of participation in this research study. Volunteers will otherwise not receive any injury compensation, only medical care. Volunteers will be encouraged to discuss this issue thoroughly with the principal investigator before they enroll in this study. This medical care does not constitute a waiver or release of volunteer's legal rights.

Emergency Medical Care

If a volunteer needs emergency medical care while at the research site, he/she is entitled to receive such care at the research site and/or elsewhere if necessary. If the injury or illness requiring emergency care was caused by the research, any medical care provided by the RCAF and/or AFRIMS will be at no cost.

Non-Emergency Care

Any volunteer who believes he/she has an injury or illness caused by the research should inform the Principal Investigator. If out of pocket for medical care are paid for injuries caused by this research study, the volunteer may contact the Principal Investigator to request reimbursement. Reimbursement cannot be guaranteed, however. The Principal Investigator will coordinate with the US Army Medical Research and Materiel Command (USAMRMC) Office of the Staff Judge Advocate (legal office), in Fort Detrick, Frederick, Maryland, to process a request for reimbursement. Volunteers may also contact the legal office directly.

The informed consent document will state, "If you are hurt or get sick because of this research study, you can receive medical care at an RCAF or Cambodian government hospital or clinic free of charge. You will be treated for injuries that are directly caused by the research study. The Army will not pay for your transportation to and from the hospital or clinic. If you have questions about this medical care, talk to the principal investigator for this study. If you pay out-of-pocket for medical care elsewhere for injuries caused by this research study, contact the Principal Investigator. If the issue cannot be resolved, contact the U.S. Army Medical Research and Materiel Command (USAMRMC) Office of the Staff Judge Advocate (legal office)."

9.10 Criteria for Discontinuation of Withdrawal of a Volunteer

Any volunteer may be discontinued from the study at any time at the discretion of the investigator if he/she feels it is in the best interest of the volunteer or if in the judgment of the investigator continuing in the study would be harmful and/or inappropriate for the volunteer or if a patient changes his/her mind after signing the consent form. Volunteers may withdraw consent at any time throughout the course of the study. If a volunteer wishes to withdraw from the study he/she will be asked whether the samples already collected can be used. If the volunteer does not wish his/her samples to be analyzed they will be removed.

While the study team will make every effort to follow subjects with weekly visits to avoid absenteeism, 100% follow-up can of course not be guaranteed. The fact that many

subjects will be on active duty and have accountability to their units will reduce this risk substantially. Further, patients that become febrile at any time during the study will be evaluated for malaria - this is the purpose of unscheduled visits for all patients with fever. Thus it is unlikely that malaria cases will be missed, unless the patient seeks care from the private sector. Volunteers will be counseled extensively on this at enrollment, and the team of investigators and unit liaisons will ensure that volunteers do not seek care at outside facilities. In the event that volunteers seek care outside the study, every effort will be made to capture the information at regularly scheduled follow-ups, and also through medical record review (if available) from any care received without the knowledge of the study team.

10. ADMINISTRATIVE PROCEDURES

10.1 Institutional Review Board and Volunteer Registry Database

The protocol and informed consent documents will be provided for the review and approval. The protocol will require scientific review and approval by the committee at AFRIMS. The protocol will undergo ethical review and require approval by the U.S. Army Medical Research and Materiel Command's Human Research Protection Office (HRPO), WRAIR IRB, the National Ethics Committee for Health Research Health IRB# 1 (FWA# 00010451, IRB # 00003143). Any modifications that could potentially increase risk to volunteers must be submitted to WRAIR DHSP and the USAMRMC ORP HRPO for approval prior to implementation. All amendments must be submitted for review and approval by all institutional review boards. Amendments will be submitted to all institutional review boards at the time of the needed change.

If modifications are required, they will be submitted in writing to the USAMRMC Office of Research Protections, the WRAIR Division of Human Volunteers Protection and the National Ethics Committee Health Research, Phnom Penh, Cambodia. A revised consent form will accompany any request for modification that changes any issue addressed in the currently approved consent form. All amendments to the protocol and informed consent form, which require regulatory and/or IRB/IEC approval/favorable opinion, must be reviewed and approved by IRB/IEC and/or local authorities before being implemented. Amendments should not be implemented until all necessary approvals have been obtained, except where necessary to eliminate an immediate hazard(s) to study volunteers. All amendments to the protocol must be submitted to the Human Research Protection Office (HRPO) and the WRAIR IRB after review and approval at the local level, but before implementation. This amendment will receive either full Human Research Protection Office (HRPO) review and WRAIR IRB review or expedited review by the Acting Chair, as appropriate. The amendment may be implemented after WRAIR Commander final authorization.

A copy of the continuing review report (at least annually) of this research protocol will be completed, upon request of the WRAIR IRB and the local IRB approval notification will be submitted to the WRAIR IRB. After the continuing review report is approved by the WRAIR IRB, the approved CRR, as well as the local IRB approval notification will be submitted to the USAMRMC ORP HRPO. The final study report and local IRB approval notification will be submitted to the WRAIR IRB. Once approved, a copy of the final study report and local IRB approval notification will be submitted to the USAMRMC ORP HRPO. The knowledge of any pending compliance inspection/visit by the OHRP, or other government agency concerning clinical investigation or research, the issuance of Inspection Reports, warning letters or actions taken by any Regulatory Agencies including legal or medical actions and any instances of serious or continuing noncompliance with the regulations or requirements will be reported immediately to WRAIR DHSP and the USAMRMC ORP HRPO.

It is the policy of USAMRMC that data sheets are to be completed on all volunteers participating in research for entry into the U.S. Army Medical Research and Materiel Command Volunteer Registry Database. The information to be entered into this confidential database includes name, address, social security number, study name, and

dates. The purpose of the database is twofold: first, to readily answer questions concerning an individual's participation in research sponsored by the USAMRMC; and second, to ensure that the USAMRMC can exercise its obligation to ensure research volunteers are adequately warned (duty to warn) of risks and to provide new information as it becomes available. The information will be stored at the USAMRMC for a minimum of 75 years.

10.2 Study Medication Accountability

Test article will be purchased commercially, and allocated to treatment groups by the AFRIMS Research Pharmacist who will label them as “2 Day” or “3 Day” accordingly. Test article will be maintained securely in a locked cabinet by the study team at all times until administered. The study team will maintain a log documenting test article administration.

10.3 Disposition of Data

See section 6.11 above.

10.4 Access to Source Data/Documents

The investigators, medical monitor, and other study personnel assigned from The Royal Cambodian Armed Forces and the National Center for Parasitology, Entomology and Malaria Control (CNM) and their respective representatives are authorized access to the study data as part of their duties and part of their responsibility to protect human volunteers in research. The investigators, medical monitor, members of the WRAIR IRB, representatives of the U.S. Army, MRMC, US Army Medical Materiel Development Activity (USAMMDA), representatives of regulatory agencies, and other government agencies are authorized access to the study data as part of their duties and part of their responsibility to protect human volunteers in research.

10.5 Certification of Translation (where applicable)

Investigators will provide documentation that the foreign language version of the consent form is an accurate translation. Documentation of translation will be provided along with the English and foreign language version of the consent forms. The documentation of translation will include the following statement, *“I certify that this is an accurate and true translation”* as well as the signature, name, address, phone number and, if available, fax number of the translator or following AFRIMS'SOP.

10.6 Protocol Amendments

Any change or amendment to the protocol affecting study volunteers, study objectives, study design, study procedures, or significant administrative aspects will require a formal amendment to the protocol. The protocol must be revised to concur with the amendment.

Such amendment will be submitted to the WRAIR IRB for review and approval and then forwarded to the USAMRMC ORP HRPO.

Any amendments to the protocol, consent form and/or questionnaires, including a change to the PI, will be submitted to the WRAIR IRB, and local IRB for review and approval and then the USAMRMC ORP HRPO for second level review (This applies to Greater than minimal risk studies; for Not greater than minimal risk studies, WRAIR IRB and the local IRB will review and provided written notification to the USAMRMC ORP HRPO). The protocol will be fully revised to concur with the amendment and submitted along with all other revised documents. Approval will be obtained prior to implementation, unless required to eliminate apparent immediate hazards.

The Informed Consent Form will be revised to concur with any significant amendment that directly affects volunteers, and will also be reviewed and approved with the amendment. New volunteers enrolled in the study will be consented with the most recent approved consent form. Volunteers already enrolled in the study will be informed about the revision and, depending on the impact of the amendment, may be asked to re consent. This may be accomplished by repeating the consent process with the revised consent form with attention given to the changes, or it may be done using an addendum consent that states the revision or new information. The new document will be signed, placed in the study record, and a copy given to the volunteer.

Administrative changes to the protocol are corrections and/or clarifications that have no effect on the way the study is to be conducted. Such administrative changes will be submitted to the WRAIR IRB for review and approval prior to implementation.

10.7 Protocol Deviations

The USAMRMC ORP HRPO requires that deviations that involve risk to volunteers or affect the scientific integrity of the study be promptly reported. This study will be conducted in accordance with this protocol unless modified in writing. The protocol will not be modified or abandoned without consultation with the sponsor and notification of the principal investigators, volunteer to the terms outlined below.

- **Protocol Amendment:** A written description of a change(s) to or formal clarification of a protocol. This includes the addition of investigators.

- **Protocol violations** occur when: there is non-adherence to the protocol that results in a significant added risk to the study volunteer; or, the study volunteer or investigator has failed to adhere to significant protocol requirements; however, the study volunteer is enrolled and/or continuing in the study without prior sponsor approval; or there is non-adherence to GCP.

- **Protocol deviations** occur when: there is non-adherence to study procedures or schedules, as specified by the protocol, which do not involve inclusion/exclusion or primary endpoint criteria. The significance of the deviation is based on the frequency of the deviation and/or impact on study objectives. Deviations include: events that are outside of the control of the investigator (e.g. study volunteer missed visit

window) or study parameters that are not part of primary endpoint (e.g. respiratory rate) not performed at a visit.

Protocol deviations will be reported to the IRBs as part of the annual report / continuing review report. Any violation or deviations to the protocol that may have an effect on the safety or rights of the volunteer or the integrity of the study must be reported to the Cambodia NEHCR and the WRAIR IRB (who will report to the USAMRMC ORP HRPO) as soon as the deviation affecting volunteer safety, and/or violation is identified.

10.8 Publication Policy

Results of this study will be presented in scientific forums orally and in written publications in scientific journals. No identifying information for any of the volunteers in the study will be included in any presentation of data or photographs. Publications will be submitted as per Command review policy.

10.9 Responsibilities of Study Personnel

All named personnel are fully qualified to perform the following assigned roles. The Principal Investigators will ensure that all assigned personnel maintain required trainings, licensures and certifications throughout the study. All duties will be performed in accordance with GCP Guidelines.

The Principal Investigators will be responsible for all aspects of the study to include: Protocol design to include all related documents (such as the consent form, case report form, standard operating procedures, etc); supervision and monitoring of research staff; protocol compliance and QA/QC plan execution; timely and accurate reporting of AEs (including SAEs) to IRBs and management of the respective organizations as outlined in the protocol. PIs will also be responsible for clinical and scientific aspects of the study to include patient care, data analysis, interpretation and manuscript preparation; continuing review and final study reports and publication. PIs will liaise with study personnel from the different organizations listed as well as local authorities. All duties will be performed in accordance with GCP Guidelines.

The Associate Clinical Investigators will be responsible for multiple aspects of the study to include: Protocol design to include all related documents (such as the consent form, case report form, standard operating procedures, etc); supervision and monitoring of research staff; protocol compliance and QA/QC plan execution; timely and accurate reporting of AEs (including SAEs) to IRBs and management of the respective organizations as outlined in the protocol. AIs will also be responsible for clinical and scientific aspects of the study to include patient care, data analysis, interpretation and manuscript preparation; continuing review and final study reports and publication. AIs will liaise with study personnel from the different organizations listed as well as local authorities.

The Associate Laboratory Investigators will be responsible for multiple aspects of laboratory analysis during or arising from the study to include depending on their respective disciplines: method development; assay design; development of standard operating procedures; storage and shipment of samples (where required); data analysis and interpretation; manuscript preparation; supervision and monitoring technical staff in the conduct of procedures based on levels of established training and expertise.

Ombudsmen in this study will serve as independent advocates for subject welfare, and be present during informed consent sessions. Ombudsmen will also serve as witnesses during the informed consent process. They will also be available to subjects by telephone and/or on request to communicate questions or concerns to the investigative team. One or more ombudsmen will be selected from the civilian community, or from the RCAF as long as they are outside the chain of command of subjects being recruited. In cases of military ombudsmen, they will have sufficient rank and authority to permit an independent unbiased determination of subject welfare. For issues that cannot be resolved by the investigators, the ombudsmen will report the matter to the medical monitor and/or sponsor.

Clinical and Laboratory Research Coordinators will be responsible for coordinating procedures in the field and laboratory to include informed consent, screening and enrollment; study procedures including SOP/SSP instruction and adherence; coordinating the conduct of laboratory procedures; in addition to other duties as assigned by the investigators for which they are qualified.

The Medical Monitor and Sponsor's Medical Expert will be responsible for monitoring study volunteers from a medical perspective, reviewing and reporting all serious and unexpected adverse events, ensuring medical care for any such events should they occur, and reporting serious events to higher headquarters and/or the Sponsor.

The Clinical Study Monitor will be responsible for regular monitoring of data collection and procedures to ensure that the human volunteer protections, study procedures, laboratory, and data collection processes are of high quality and meet GCP/ICH and regulatory guidelines; and correspond with IRBs and the Sponsor as required.

Consultants may assist the study team with protocol design, data analysis, interpretation and manuscript review and preparation. Consultants will not have contact with volunteers or their individually identifiable information. Consultant laboratory investigators from outside institutions may analyze study specimens that have been stripped of all individually identifiable information, and designated with a study code to remain under control of the Principal Investigators that consulting labs will not have access to. In each case, performing laboratory consultants will obtain a determination of non-human subjects research from their respective IRBs and provide this to the IRBs of record.

10.10 Responsibilities of the Medical Monitor

Per DOD Directive 3216.2, all greater than minimal risk studies require a medical monitor. The name of the medical monitor is included in the protocol and the curriculum vitae has been provided. Note that the DOD definition of a medical monitor differs from the industry definition.

The Medical Monitor for this study is a qualified physician, other than the Principal Investigator, not associated with the protocol, who is able to provide medical care to research volunteers for conditions that may arise during the conduct of the study, and who will monitor the volunteers during the conduct of the study. The medical monitor will review serious adverse events and unanticipated problems, and forward a report to the IRB for further action.

10.11 Compliance with Laws and Regulations

The undersigned Principal and Associated Investigators have reviewed this protocol and will conduct the study in full compliance with current Good Clinical Practice Guidelines, FDA regulations, and Army regulations, as well as applicable local law.

11. REFERENCES

White, N. J.2002. The assessment of antimalarial drug efficacy. Trends in Parasitology. 18:458-64.

Taylor, et al. 1999. Malaria prophylaxis using azithromycin: a double-blind, placebo-controlled trial in Irian Jaya, Indonesia. Clinical Infectious Diseases. 28:74-81.

Ohrt, C., T. L. Richie, et al. (1997). "Mefloquine compared with doxycycline for the prophylaxis of malaria in Indonesian soldiers. A randomized, double-blind, placebo-controlled trial." Ann Intern Med **126**(12): 963-72.

Baudon, D., G. Martet, et al. (1999). "Efficacy of daily antimalarial chemoprophylaxis in tropical Africa using either doxycycline or chloroquine-proguanil; a study conducted in 1996 in the French Army." Trans R Soc Trop Med Hyg **93**(3): 302-3.

Pages, F., J. P. Boutin, et al. (2002). "Tolerability of doxycycline monohydrate salt vs. chloroquine-proguanil in malaria chemoprophylaxis." Trop Med Int Health **7**(11): 919-24.

Amendola, M. A. and T. D. Spera (1985). "Doxycycline-induced esophagitis." Jama **253**(7): 1009-11.

Bryan, J. P. (2006). "Cost considerations of malaria chemoprophylaxis including use of primaquine for primary or terminal chemoprophylaxis." Am J Trop Med Hyg **75**(3): 416-20.

Kitchener, S. J., P. E. Nasveld, et al. (2005). "Mefloquine and doxycycline malaria prophylaxis in Australian soldiers in East Timor." MJA **182**(4): 168-171.

Schlagenhauf, P., A. Tschopp, et al. (2003). "Tolerability of malaria chemoprophylaxis in non-immune travellers to sub-Saharan Africa: multicentre, randomised, double blind, four arm study." Bmj **327**(7423): 1078.

Wongsrichanalai, C., Sirichaisinthop, J., Karwacki, J. J., Congpuong, K., Miller, R. S., Pang, L., and Thimasarn, K. Drug resistant malaria on the Thai-Myanmar and Thai-Cambodian borders. Southeast Asian J Trop Med Public Health 2001;(32): 41-49.

Wongsrichanalai, C., Pickard, A. L., Wernsdorfer, W. H., and Meshnick, S. R. Epidemiology of drug-resistant malaria. Lancet Infect Dis 2002;(2): 209-218.

Hyde, J. E. Mechanisms of resistance of Plasmodium falciparum to antimalarial drugs. Microbes Infect 2002;(4): 165-174.

Price, R. N., and Nosten, F. Drug resistant falciparum malaria: clinical consequences and strategies for prevention. Drug Resist Update 2001;(4): 187-196.

Bloland, P. B., Lackritz, E. M., Kazembe, P. N., Were, J. B., Steketee, R., and Campbell, C. C. Beyond chloroquine: implications of drug resistance for evaluating malaria therapy efficacy and treatment policy in Africa. J Infect Dis 1993;(167): 932-937.

Shretta, R., Omumbo, J., Rapuoda, B., and Snow, R. W. Using evidence to change antimalarial drug policy in Kenya. Trop Med Int Health 2000;(5): 755-764.

Omar, S. A., Adagu, I. S., Gump, D. W., Ndaru, N. P., and Warhurst, D. C. Plasmodium falciparum in Kenya: high prevalence of drug-resistance-associated polymorphisms in hospital admissions with severe malaria in an epidemic area. Ann Trop Med Parasitol 2001;(95): 661-669.

Mberu, E. K., Mosobo, M. K., Nzila, A. M., Kokwaro, G. O., Sibley, C. H., and Watkins, W. M. The changing in vitro susceptibility pattern to pyrimethamine/sulfadoxine in Plasmodium falciparum field isolates from Kilifi, Kenya. Am J Trop Med Hyg 2000;(62): 396-401.

Khan, B., Omar, S., Kanyara, J. N., Warren-Perry, M., Nyalwidhe, J., Peterson, D. S., Wellems, T., Kaniaru, S., Gitonga, J., Mulaa, F. J., and Koech, D. K. Antifolate drug resistance and point mutations in Plasmodium falciparum in Kenya. Trans R Soc Trop Med Hyg 1997;(91): 456-460.

Druilhe, P., Pradier, O., Marc, J. P., Miltgen, F., Mazier, D. and Parent, G. Levels of antibodies to Plasmodium falciparum sporozoite surface antigens reflect malaria transmission rates and are persistent in the absence of reinfection. Infect Immun 1986. (53): 393-397.

Webster, H. K., Gingrich, J. B., Wongsrichanalai, C., Tulyayon, S., Suvarnamani, A., Sookto, P. and Permpanich, B. Circumsporozoite antibody as a serologic marker of Plasmodium falciparum transmission. Am J Trop Med Hyg 1992. (47): 489-497.

Archibald, C. P., Mak, J. W., Mathias, R. G. and Selvajothi, S. Antibodies to Plasmodium falciparum in an indigenous population from a malaria endemic area of Malaysia. Acta Trop 1990. (48): 149-157.

Del Giudice, G., Engers, H. D., Tougne, C., Biro, S. S., Weiss, N., Verdini, A. S., Pessi, A., Degremont, A. A., Freyvogel, T. A., Lambert, P. H. et al. Antibodies to the repetitive epitope of Plasmodium falciparum circumsporozoite protein in a rural Tanzanian community: a longitudinal study of 132 children. Am J Trop Med Hyg 1987. (36): 203-212.

Tarning J, Ashley EA, Lindegardh N, Stepniewska K, Phaiphun L, et al. Population pharmacokinetics of piperaquine after two different treatment regimens of dihydroartemisinin-piperaquine in patients with acute uncomplicated Plasmodium falciparum malaria in Thailand. Antimicrob Agents Chemother. 2008;52:1052–61.

Hung TY, Davis TM, Ilett KF, Karunajeewa H, Hewitt S, et al. Population pharmacokinetics of piperaquine in adults and children with uncomplicated falciparum or vivax malaria. Br J Clin Pharmacol. 2004;57:253–262.

Julien Zwang et al. Safety and Efficacy of Dihydroartemisinin-Piperaquine in Falciparum Malaria: A Prospective Multi-Centre Individual Patient Data Analysis. PLoS One. 2009; 4(7): e6358.

Wongsrichanala C, Meshnick SR. Declining Artesunate-Mefloquine Efficacy against Falciparum Malaria on the Cambodia–Thailand Border. Emerg Infect Dis. 2008 May; 14(5): 716–719.

Krudsood S *et al.* Dose ranging studies of new artemisinin-piperaquine fixed combinations compared to standard regimens of artemisinin combination therapies for acute, uncomplicated falciparum malaria. 2007; 38 (6): 971-978.

Luxemburger C, Brockman A, Silamut K, Nosten F, van Vugt M, Gimenez F, Chongsuphajaisiddhi T, White NJ. Two patients with falciparum malaria and poor in vivo responses to artesunate. Trans R Soc Trop Med Hyg. 1998 Nov-Dec;92(6):668-9.

Gogtay NJ, Kadam VS, Karnad DR, Kanbur A, Kamtekar KD, Kshirsagar NA. Probable resistance to parenteral artemether in Plasmodium falciparum: case reports from Mumbai (Bombay), India. Ann Trop Med Parasitol. 2000 Jul;94(5):519-20.

Sahr F, Willoughby VR, Gbakima AA, Bockarie MJ. Apparent drug failure following artesunate treatment of Plasmodium falciparum malaria in Freetown, Sierra Leone: four case reports. Ann Trop Med Parasitol. 2001 Jul;95(5):445-9.

Meshnick SR. Artemisinin: mechanisms of action, resistance and toxicity. Int J Parasitol. 2002 Dec 4;32(13):1655-60.

Noedl H, Se Y, Schaecher K, et al. Artemisinin Resistance in Cambodia 1 (ARC1) Study Consortium. Evidence of artemisinin-resistant malaria in western Cambodia. N Engl J Med. 2008 Dec 11;359(24):2619-20.

Baird JK, Hoffman SL (2004). Primaquine therapy for malaria. Clinical and Infectious Diseases, 39:1336–1345.

Goller JL et al. (2007). Regional differences in the response of Plasmodium vivax malaria to primaquine as anti-relapse therapy. American Journal of Tropical Medicine and Hygiene, 76:203–207.

WHO (2007). Methods for Surveillance of Antimalarial Drug Efficacy.

Alving, A. S., C. F. Johnson, et al. (1960). "Mitigation of the haemolytic effect of primaquine and enhancement of its action against exoerythrocytic forms of the Chesson strain of Plasmodium vivax by intermittent regimens of drug administration: a preliminary report." Bull World Health Organ 22: 621-31.

Kyaw, M. P., O. Myint, et al. (1994). "The use of primaquine in malaria infected patients with red cell glucose 6-phosphate dehydrogenase (G6PD) deficiency in Myanmar." Southeast Asian J Trop Med Public Health 25(4): 710-3.

Charoenlarp, P., S. Areekul, et al. (1972). "The haemolytic effect of a single dose of 45 mg of primaquine in G-6-PD deficient Thais." J Med Assoc Thai 55(11): 631-8.

Reeve, P. A., H. Toaliu, et al. (1992). "Acute intravascular haemolysis in Vanuatu following a single dose of primaquine in individuals with glucose-6-phosphate dehydrogenase deficiency." J Trop Med Hyg 95(5): 349-51.

Ziai, M., G. H. Amirhakimi, et al. (1967). "Malaria prophylaxis and treatment in G-6-PD deficiency. An observation on the toxicity of primaquine and chloroquine." Clin Pediatr (Phila) 6(4): 242-3.

US Centers for Disease Control and Prevention, Guidelines for Treatment of Malaria in the United States, Updated May 18, 2009. http://www.cdc.gov/malaria/pdf/treatmenttable.pdf

Sabeti PC, Reich DE, Higgins JM, Levine HZ, Richter DJ, Schaffner SF, Gabriel SB, Platko JV, Patterson NJ, McDonald GJ, Ackerman HC, Campbell SJ, Altshuler D, Cooper R, Kwiatkowski D, Ward R, Lander ES. Detecting recent positive selection in the human genome from haplotype structure. Nature. 2002 Oct 24; 419(6909):832-7.

Cummings KC, Mohle-Boetani J, Royce SE, Chin DP. Movement of tuberculosis patients and the failure to complete antituberculosis treatment. Am J Respir Crit Care Med. 1998 Apr;157(4 Pt 1):1249-52.

Noedl H, Wernsdorfer WH, Miller RS and Wongsrichanalai C. Histidine rich protein II, a novel approach to anti-malarial drug susceptibility testing. Antimicrob Agent Chemother Antimicrob Agents Chemother. 2002 Jun; 46(6): 1658-64.

Bland JM, Altman DG. Comparing methods of measurement: why plotting difference against standard method is misleading. Lancet. 1995 Oct 21;346(8982):1085-7.

Liu C et al. “Pharmacokintetics of Piperaquine after single and multiple oral Administrations in Healthy Volunteers” Yakugaku Zasshi 2007; 127 (10) 1709-1714

Bangchang KN, Karbwang J, & Back DJ: Primaquine metabolism by human liver microsomes: effect of other antimalarial drugs. Biochem Pharmacol 1992; 44:587-590.

Barradell LB & Fitton A: Artesunate: a review of its pharmacology and therapeutic efficacy in the treatment of malaria. Drugs 1995a; 50:714-771.

Mytton OT, Ashley EA, et al. Short Report: Electrocardiographic Safety Evaluation of Dihydroartemisinin–Piperaquine in the Treatment of Uncomplicated falciparum Malaria. Am. J. Trop. Med. Hyg., 77(3), 2007, pp. 447–450

Janssens B , van Herp M et al., A randomized open study to assess the efficacy and tolerability of dihydroartemisinin–piperaquine for the treatment of uncomplicated falciparum malaria in Cambodia. Tropical Medicine & International Health, 12 (2), 2007, pp. 251 – 259.

MMV Press Release 2009 - http://www.asia-lifesciences.com/press-174-sigmatau-industriefarmaceuticheriunite-emea-novelantimalarial-press6.html

12. APPENDICES

Appendix A. Study Drug – Duo-cotecxin Label

DUO-COTECXIN®

DUO-COTECXIN®

Antimalarial

PRESENTATION

DUO-COTECXIN® TABLETS

For Adult

(Dihydroartemisinin 40mg + Piperaquine Phosphate 320mg)

8 Tablets/Packet, 480Pkts/Carton

PHARMACOLOGY

DUO-COTECXIN® is a synergistic combination of an artemisinin derivative and a new bisquinoline compound; it is active against the asexual forms of plasmodium, schizonts and gametocytes. Its action on schizonts insures a rapid decrease of pathogenic parasitaemia which leads to a quick disappearance of clinical signs; its action on gametocytes prevents contamination.

The absorption of DUO-COTECXIN® after oral intake is rapid and complete; it is extensively distributed in all tissues. Half life of DHA is very short (around 2 hours) whereas PPQ is long (around 9 days).

INDICATIONS

DUO-COTECXIN® is indicated for the treatment of all kinds of malaria, as long as patients can take oral medications. It is particularly recommended in the case of multi-resistant plasmodium falciparum malaria.

ADMINISTRATION AND DOSAGE

Oral administration.

Patient should follow doctor's instruction. The recommended dosage is in the following table:

	Adults	Children 16 - 11	Children 11 – 6
Day 1	3 tabs	2 tabs	1.5 tab
Day 2	3 tabs	2 tabs	1.5 tab
Day 3	2 tabs	2 tabs	1 tab
Total	8 tabs	6 tabs	4 tabs

ADVERSE EFFECTS

Few cases of adverse effects have been reported after administration of DUO-COTECXIN®. Most of them are related to PPQ affecting the digestive tract (nausea, diarrhea, loss of appetite, etc.). Rare allergic reactions have also been reported (rash, pruritus, etc.).

CONTRAINDICATIONS

As all new drugs, DUO-COTECXIN® is not recommended during the first trimester of pregnancy unless your doctor considers the risk of the disease to be greater.

A new course of DUO-COTECXIN® should not be taken within four weeks after the first treatment.

STORAGE

Keep all medicines out of the reach of children.

Preserve in tight and lightproof containers.

Store in a cool and dry place below 30□.

SHELF LIFE

2 years.

Manufactured by Zhejiang Holley Nanhu Pharmaceutical Co., Ltd
205, Yunhai Road, Economy Development Zone, Jiaxing City, P. R. China
Under license of Holleypharm

Appendix B. Malaria Treatment in the Kingdom of Cambodia, Treatment Guideline for Referral Hospital, Health Centre and Health Post in Cambodia

Jointly prepared by
National Malaria Centre, Phnom Penh
& World Health Organization
Revised Edition, December 2008, Supplement

(submitted as an Attachment – "Current Malaria Treatment Guidelines - Cambodia, Dec 2008.DOC")

Appendix C. Guidance for Industry - Malaria: Developing Drug and Nonvaccine Biological Products for Treatment and Prophylaxis. DRAFT GUIDANCE (submitted as an Attachment)

Appendix D. Recommendations - Cambodia Drug Policy Workshop, PHNOM PENH, 08-09 APRIL 2010 (submitted as an Attachment)

Appendix E. Source Documents / Case Report Forms (submitted as an Attachment)
